# Synthesis of novel amidines *via* one-pot three component reactions: Selective topoisomerase I inhibitors with antiproliferative properties

**DOI:** 10.3389/fchem.2022.1039176

**Published:** 2022-11-18

**Authors:** Essmat M. El-Sheref, Hendawy N. Tawfeek, Alaa A. Hassan, S. Bräse, Mohammed A. I. Elbastawesy, Hesham A. M. Gomaa, Yaser A. Mostafa, Bahaa G. M. Youssif

**Affiliations:** ^1^ Chemistry Department, Faculty of Science, Minia University, El Minia, Egypt; ^2^ Institute of Biological and Chemical Systems, IBCS-FMS, Karlsruhe Institute of Technology, Karlsruhe, Germany; ^3^ Department of Pharmaceutical Organic Chemistry, Faculty of Pharmacy, Al-Azhar University, Assiut, Egypt; ^4^ Pharmacology Department, College of Pharmacy, Jouf University, Sakaka, Saudi Arabia; ^5^ Pharmaceutical Organic Chemistry Department, Faculty of Pharmacy, Assiut University, Assiut, Egypt

**Keywords:** one-pot 3CR, 4-azido-quinolin-2(1*H*)-ones, topo, antiproliferative, viability, heterocycles, cancer

## Abstract

Novel series of amidines were synthesized *via* the interaction between alicyclic amines, cyclic ketones, and a highly electrophilic 4-azidoquinolin-2(1*H*)-ones without any catalyst or additive. All the obtained products were elucidated based on NMR spectroscopy, mass spectrometry, and elemental analysis. The reaction conditions were optimized using cyclohexanone (2), piperidine (3a), and 4-azido-quinolin-2(1*H*)-one (1a) under an air atmosphere. The new compounds 4a-l and 5a-c were tested for antiproliferative activity against four cancer cell lines using doxorubicin as a reference drug. The most potent derivatives were compounds 4b, 4d, 4e, 4i, and 5c, with GI_50_ ranging from 1.00 µM to 1.50 µM. Compound 5c was the most effective derivative against the four cancer cell lines, outperforming doxorubicin. The compounds 4b, 4d, 4e, 4i, and 5c were studied further as topoisomerase I and IIα inhibitors. The compounds tested showed selective inhibition of topo I over topo IIα. Finally, docking studies explain why these compounds prefer topo I over topo IIα.

## Graphical Abstract

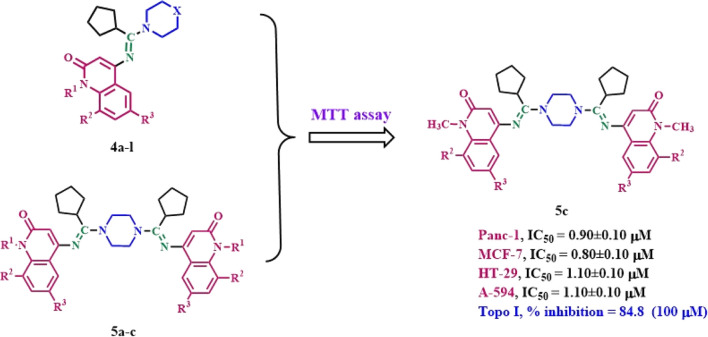

## 1 Introduction

Heterocyclic scaffolds, especially nitrogen-containing heterocyclic compounds, play an important role in the design and synthesis of novel drugs because of their utility for various biological receptors with a high degree of binding affinity (Kerru et al., 2020), among various heterocyclic compounds. Quinolines have gained the largest attention. Quinolines benzo[*b*]pyridines are naturally plant-derived, and many of their derivatives can also be lab synthesized (Ajani et al., 2022). Throughout the 20th century, quinolines’ chemistry and biological applications and their derivatives have been subjected to intense studies from different research groups (Matada et al., 2021).

The quinolone (oxo-quinoline) ring system is one of the most preferential heterocycles in drug research (Dhiman et al., 2019). Quinolones and quinolone analogs are the core structural elements of many pharmaceutical agents (Aly et al., 2021). Also, quinolones are one of the most commonly prescribed classes of antibacterial in the world and are used to treat various human diseases (Sadowski et al., 2022). Quinolone derivatives have been extensively subjected to medicinal chemistry research due to their privileged structure that shows various pharmacological activities such as anticancer (Elbastawesy et al., 2019; Elbastawesy et al., 2020), antibacterial (Elbastawesy et al., 2021), anti-tubercular (Liu et al., 2021), antimalarial (Nilsen et al., 2013), anti-HIV (Wang et al., 2019), anti-HCV, and many other miscellaneous biological activities (Daneshtalab and Ahmed 2012).

DNA topoisomerases (Topos I, II) are nuclear enzymes that help restore DNA topology by alleviating torsional strains produced during replication, transcription, segregation, and recombination processes (Vos et al., 2011). Among two types, and in the absence of ATP and Mg elements, human type I topoisomerase (topo I) may split and rejoins a single DNA strand. On the other hand, and in the presence of ATP and Mg, type II (topo II), which behaves as a homodimer, divides and rejoins the double DNA strand. Based on their cellular activities, topo II could be further subdivided into two isoforms: topo IIα, which is frequently linked to proliferating cells, and topo IIβ, which is not (Goftar et al., 2014). Even though topoisomerase inhibitors such as camptothecin, etoposide, and irinotecan have been used as anticancer drugs for decades, they have well-defined imperfections, such as dose-limiting toxicities with many major side effects (Briasoulis et al., 2005). In this vain, topos have remained a promising target in medicinal chemistry due to high antitumor selectivity when compared with other DNA damaging agents(Dehshahri et al., 2020).

**FIGURE 1:**
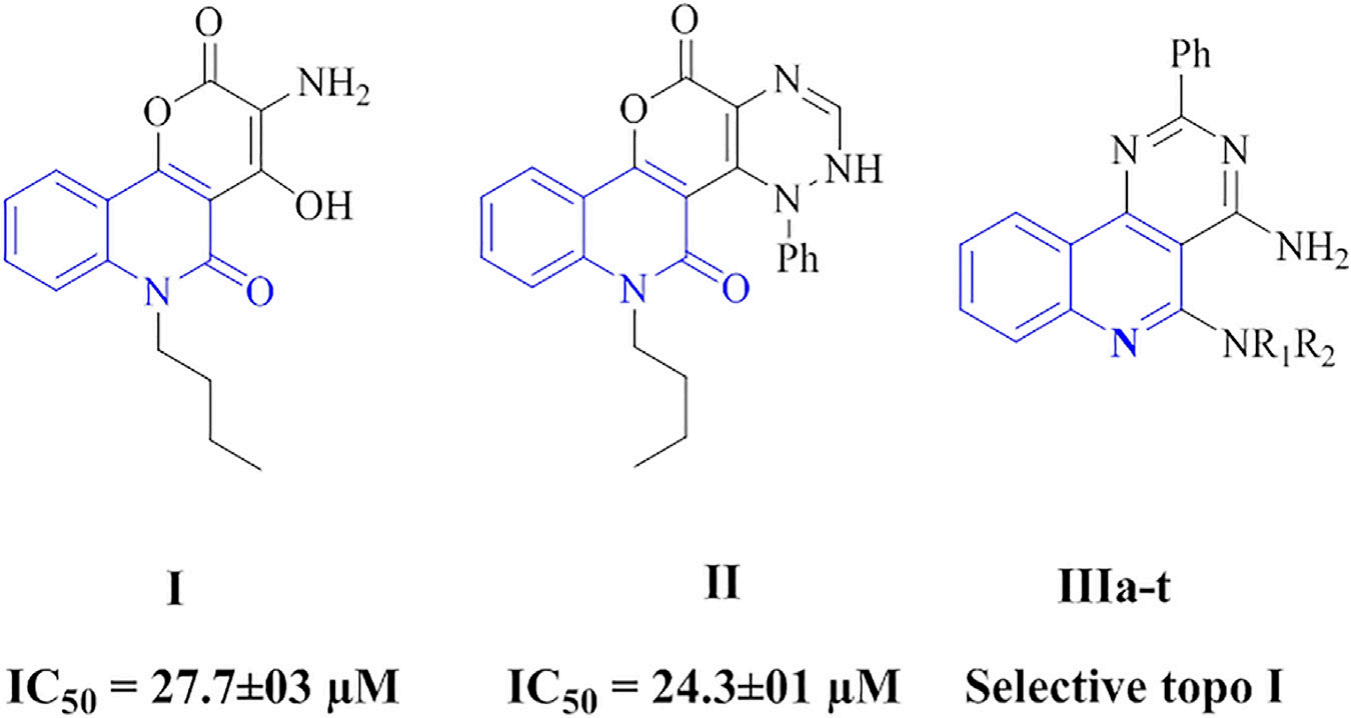
Reported quinolones-based compounds targeting topoisomerases.

Over the past few years, several quinoline-containing compounds have been reportable as potential antitumor agents (Panda and Chakroborty 2020). Further, the quinolone scaffold plays an essential role in antitumor drug development. Their derivatives have shown remarkable results through different mechanisms of action, such as growth inhibitors by cell cycle arrest, apoptosis, inhibition of angiogenesis, disruption of cell migration, and modulation of nuclear receptor responsiveness (Schmid 2021). The anticancer potential of many of these derivatives has been demonstrated on various cancer cell lines. The strength of quinoline scaffold in anticancer drug development is evident from clinically used anticancer drugs like Irinotecan, Topotecan, Camptothecin, etc. (Sissi and Palumbo 2003). Also, It has been reported that many 2-quinolones are known as potent antitumor agents through targeting topoisomerase enzymes with acceptable results and high affinity in docking studies which may be considered a therapeutic promise (compounds **I** and **II**, Figure 1) (Saeed et al., 2020). We recently reported on the design and synthesis of a novel series of pyrimido[5,4-*c*]quinoline derivatives (Compounds **IIIa-t**, Figure 1) as antiproliferative agents targeting topoisomerase (topo) I and topo IIα. Most of the compounds tested exhibited selective topo I inhibitory activity while having weak topo II inhibitory activity, with some new compounds demonstrating better topo I inhibitory activity than the reference camptothecin (Mekheimer et al., 2022).

**FIGURE 2:**
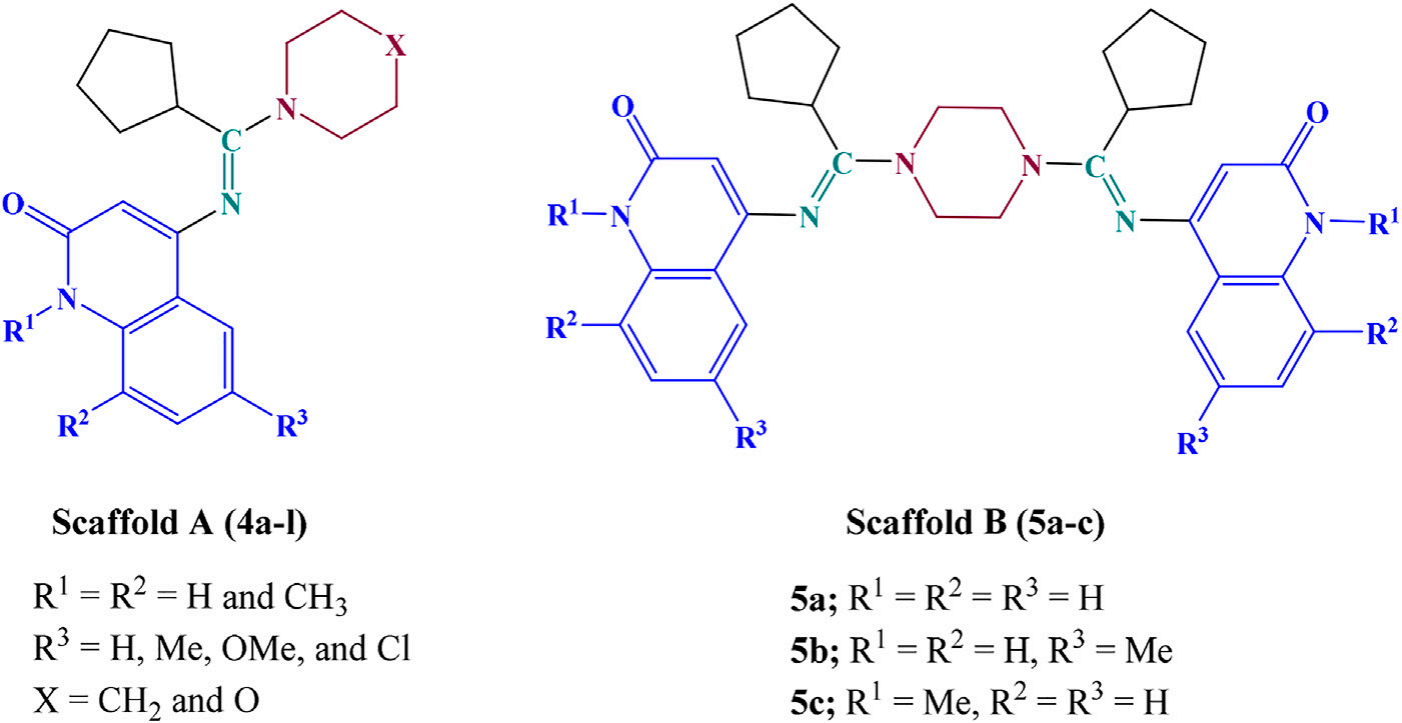
Structures of new quinoline-based derivatives 4a-l and 5a-c.

Based on the data presented above, we developed some new quinolone-based derivatives as antiproliferative agents that target topo I and topo IIα. The new compounds are of two Scaffolds; the Scaffold A compounds (4a-l) were synthesized from the reaction of azido quinoline intermediates, cyclohexanone, and piperidine or morpholine (Figure 2). The Scaffold B compounds (5a-c, Figure 2) were synthesized from the reaction of azido quinoline intermediates, cyclohexanone, and piperazine. The effect of new 4a-l and 5a-c on normal cell lines will be assessed using the MCF-10A (human mammary gland epithelial) cell line in a cell viability assay. The newly synthesized compounds will be tested for antiproliferative activity against four cancer cell lines: A-549 (epithelial cancer cell line), MCF-7 (breast cancer cell line), Panc-1 (pancreas cancer cell line), and HT-29 (colon cancer cell line) to calculate the IC_50_ of each compound using doxorubicin as the reference. The most active compounds’ topo I and IIα inhibitory activity will be determined at 100 μM and 20 µM concentrations using Camptothecin as a positive reference for topo I inhibitory assay and Etoposide for topo IIα inhibitory assay.

## 2 Result and discussion

### 2.1 Chemistry

**SCHEME 1:**
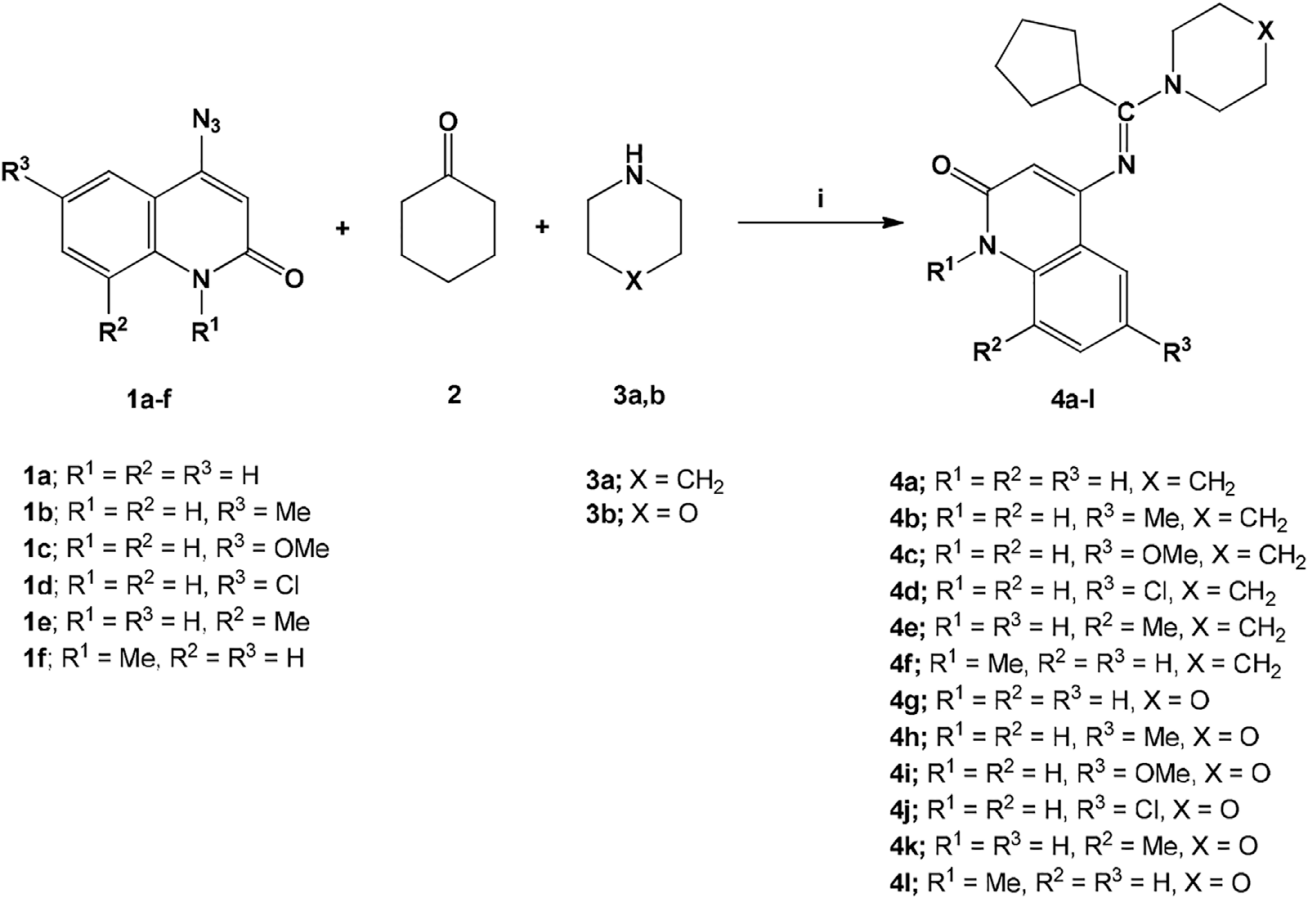
Reactions of azides **1a-f**, cyclohexanone (**2**) and cycloamines **3a,b**, i) 1 mmol of 4-azido-quinolin-2-one (**1a-f**),1.5 mmol of cyclohexanone (**2**) and 2 mmol of **(3a,b)**/DMF, 60 ^o^C, stirring for 13 h.

Our goal in this study is to develop a new series of amidines compounds **4a-l** *via* a three-component reaction involving 4-azido-quinolin-2(1*H*)-ones **1a-f** (Stadlbauer et al., 1991; Steinschifter et al., 1994; Derin et al., 2008), cyclohexanone **(2)**, and piperidine **(3a)** or morpholine **(3b)**. We begin by mixing cyclohexanone (**2**) and piperidine (**3a**) in DMF as a solvent for 1 hour at 60°C in a molar ratio (1.5:2), which results in the formation of enamines *in situ*. Then 1 mmol of 4-azido-quinolin-2(1*H*)-ones **1a-f** will be added in one portion and stirred continuously for another 12 h. The generated enamines will react with the highly electrophilic azides **1a-f** to give good yields of our target compounds **4a-l**. We conducted the reaction under several conditions to ensure the appropriate reaction conditions. However, we discovered that the proper conditions involved a three-component reaction between cyclohexanone **(2)**, piperidine **(3a)**, and 4-azido-quinolin-2(1*H*)-one **(1a)** in molar ratios (1.5:2:1) respectively, in DMF at 60°C, under atmospheric pressure, and we obtained a product (*E*) 4-((cyclopentyl(piperidin-1-yl)methylene)amino)quinolin-2(1*H*)-one (**4a**) in 90% yield. Also, the interaction with cyclopropanone, cyclobutanone and cyclopentanone was also reworked. Fortunately, it did not yield any results, confirming that the behavior of the interaction and also the occurrence of the process of the ring opening with loss a CH_2_-group to give the more stable cycloalkyl and also confirming the reaction mechanism. According to this result, we applied the above optimal conditions in the other reactions of 4-azido-2-quinolones **1b-f** with the variously produced enamines, as illustrated in Scheme 1.

**FIGURE 3:**
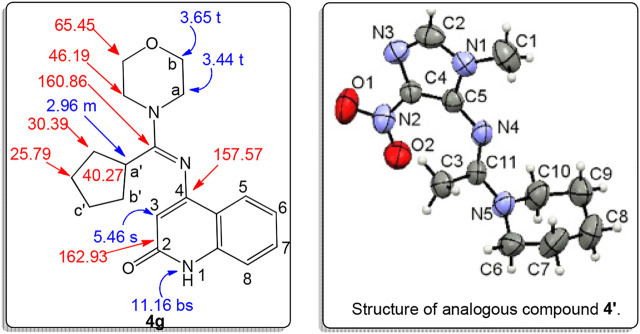
Structure of compound **4g**.

To confirm the structures of our obtained products 4-((cyclopentyl(cycloamine)methylene)-amino)substituted-quinolin-2(1*H*)-ones **4a-l**, NMR, Mass spectrometry, and elemental analysis were performed for all obtained products. All spectral data declares that the acquired molecular formula for **4a-l** is formed from one molecule of compounds **1a-f**, one molecule of cyclohexanone (**2**), and one molecule of heterocyclic amines **3a,b** with the elimination of H_2_O molecule and N_2_ molecule. On the other hand, we know that the reaction between the cycloamine and cyclohexanone to form the enamine is a reverse reaction, and there is a state of equilibrium between the products, and the reactants were reached, so excessive amounts of cyclohexanone and cycloamine were used to ensure the reaction of each azide **1a-f** with the *in situ* formed enamines. We choose compound **4g** which assigned as (*E*) 4-((cyclopentyl-(morpholino)methylene)amino)quinolin-2(1*H*)-one. This compound was formed *via* interaction between cyclohexanone (**2**), morpholine (**3b**), and 4-azido-quinolin-2(1*H*)-one (**1a**) in a molar ratio (1.5**:**2**:**1), respectively (Figure 3).

Compound **4g** exhibited a molecular formula of C_19_H_23_N_3_O_2_, which is compatible with its mass spectrometry with m/z = 325 and elemental analysis. The ^1^H NMR spectrum showed three characteristic signals as a singlet at δ_H_ = 11.16, 5.46 and 2.96 ppm, which were assigned as NH, quinolinone-H-3, and H-a' (cyclopentyl-CH), respectively. Cyclopentyl-CH was further confirmed from ^13^C NMR with the chemical shift at δ_C_ = 40.27 ppm. Also, ^1^H NMR spectrum showed two triplet signals as deshielded protons with chemical shifts at δ_H_ = 3.65 (*J* = 5.1 Hz, 4H) and 4.44 ppm (*J* = 5.1 Hz, 4H) corresponding to morpholino-H-a,b, which were further confirmed from ^13^C NMR with characteristic singlet at δ_C_ = 65.45 and 46.19 ppm for C-b and C-a, respectively. According to the chemical shif values of morpholino-protons (H-a,b), cyclopentyl protons, and stability energy in addition to comparison between our obtained products with its analogue compound **4'** (Figure 3) that was previously proven with NMR spectrums, as well as X-rays, we can only be certain that these compounds are in the *E*-form (Efimov et al., 2016). Further, three shielded signals at δ_C_ = 162.93, 160.86, and 157.57 ppm were assigned as quinolinone-C-2, C=N, and quinolinone-C-4, respectively. Through spectral analyses, we note that all values of the quinolinone ring and cyclopentyl ring, in addition to the morpholino ring, agree with the previously published values (Efimov et al., 2016; Aly et al., 2019; El-Sheref et al., 2021).

**FIGURE 4:**
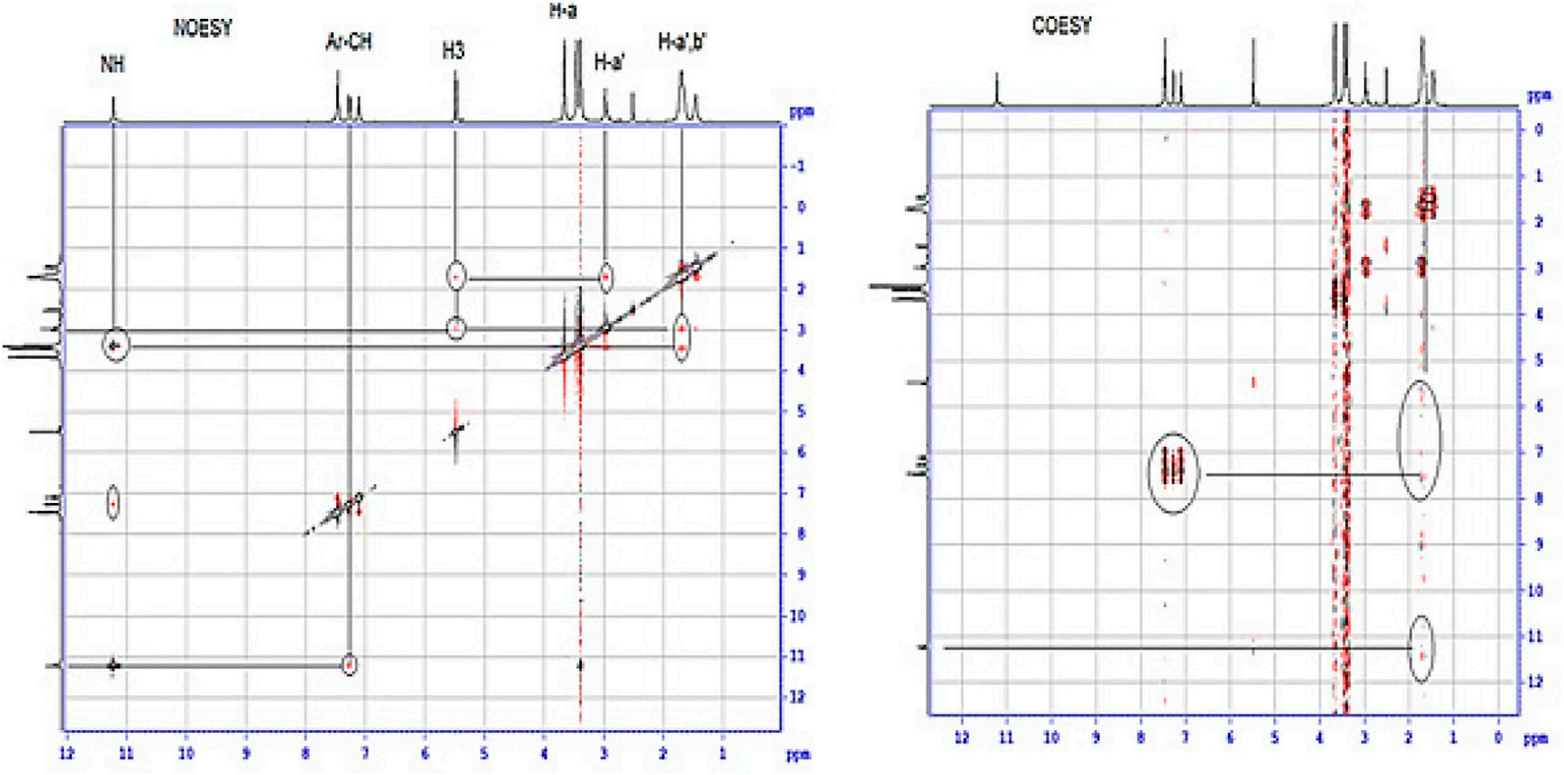
Part of NOSY and COSY spectrum for compound **4g**.

Further, The proton at δ_H_ 5.46 ppm; which assigned as H-3 give two NOESY correlation, one with the protons appears at δ_H_ 2.96 and 1.45–1.69 ppm, which are assigned as H-a' and cyclopentyl-CH_2_, and the other with the proton at δ_H_ 11.16 ppm, which assigned as NH. Further, the cyclopentyl protons give a clear COSY correlation with the quinolinone-CH prontons which appers at δ_H_ 7.09–7.42 ppm. Also, the carbon at δ_C_ 30.39 ppm give two HMBC correlation with the protons appearing at δ_H_ 2.96 (m, 1H) and 1.45–1.69 (m, 8H), and 7.30 (d, *J* = 7.4 Hz; 2H), ppm. this carbon assigned as C-b'. Also, H-a' giva HMBC correlation with the two carbons appears at δ_C_ = 162.93 and 160.86 ppm. which were assigned as C=O and C=N, respectively. Further, the cyclopentyl-CH2 give HSQC correlation with the two carbon appeare at 30.39 and 25.79 ppm. whcich were assigned as C-b' and C-c', respectively. This correlation between cyclopenty-ring and quinolinone-ring protons indicated that these protons were in same direction and confirming the *E*-structure of compound **4g** (Figure 4).

**SCHEME 2:**
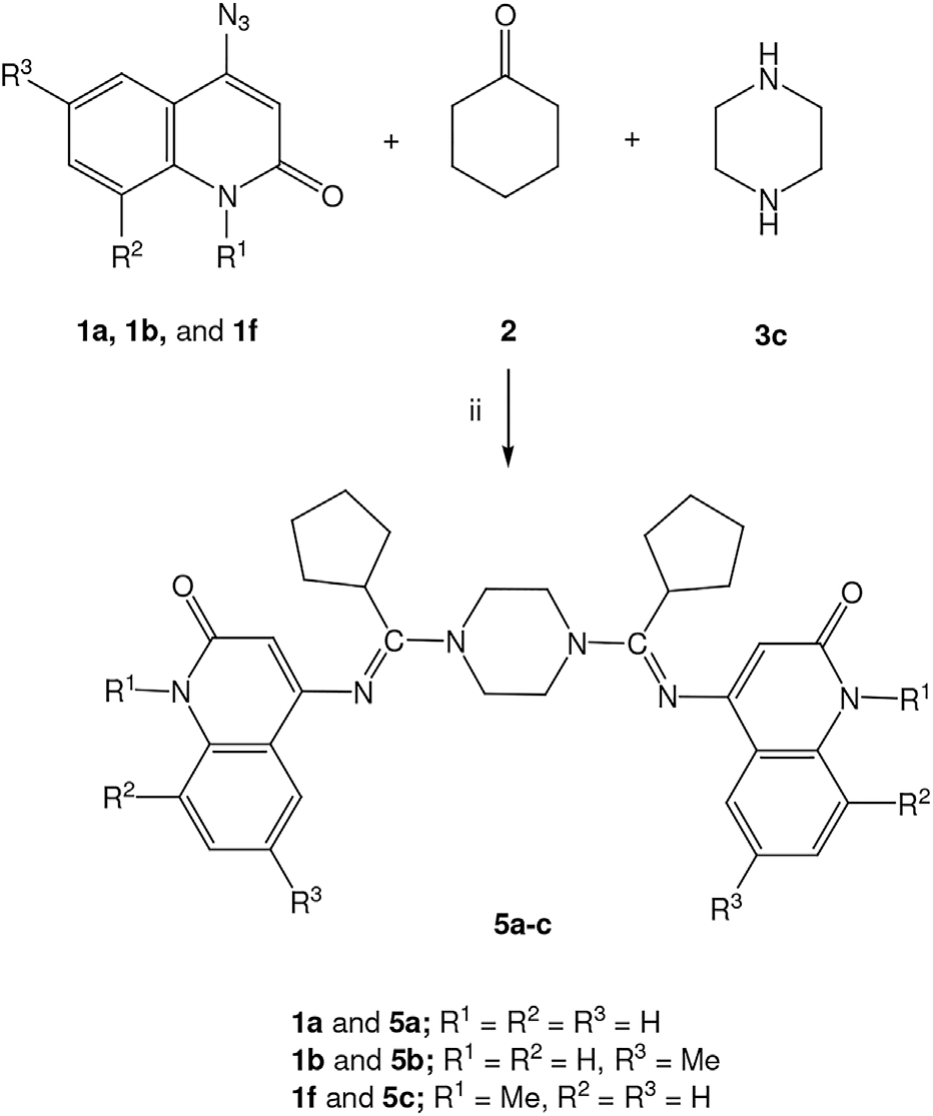
Reactions of azides **1a**, **1b**, and **1f**, cyclohexanone (**2**) and piperazine **3c**, ii) 2 mmol of 4-azido-quinolin-2-one (**1a**, **1b**, and **1f**), 2 mmol of cyclohexanone (**2**) and 2 mmol of piperazine **(3c)**/DMF, 60^o^C, stirring for 24 h.

The results we obtained from the previous reactions prompted us to conduct the reaction again with piperazine **3c** to give it the opportunity for the reaction to take place on both sides with the same previous conditions. Under the same conditions, 4-azidoquinolin-2(1*H*)-ones (**1a**, **1b**, and **1f**) reacted with cyclohexanone (**2**) and piperazine (**3c**) in molar ratio (2:2:3) respectively, in DMF at 60°C, under atmospheric pressure with stirring for 24 h to give 4,4'-((piperazine-1,4-diylbis(cyclopentylmethanylylidene))bis(azanylylidene))bis(substituted-quinolin-2(1*H*)-ones) (**5a-c)** in yields 60–73% as shown in Scheme 2.

**FIGURE 5:**
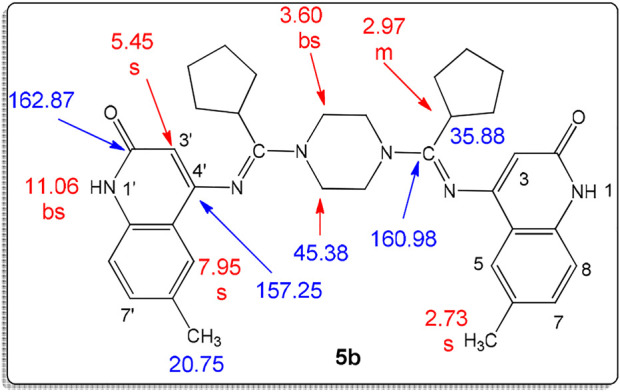
Structure of compound **5b**.

To illustrate the structures of products 5a-c, we chose compound 5b as an example (Figure 3). The structure assignment of compound 5b was based on spectral data and combustion analysis. Elemental analysis for it assigned it has a molecular formula C_36_H_42_N_6_O_2_ which is further confirmed from the mass spectrometry with m/z = 590. The ^1^H NMR spectrum of 5b clearly shows the presence of five singlet signals centered at δ_H_ = 11.06, 7.95, 5.45, 3.60, 2.97, and 2.73 ppm, which are assigned as quinolinone-NH-1,1′, quinolinone-H-5,5′, quinolinone-H-3,3′, piperazine-CH_2_, cyclopentyl-CH, and methyl groups, respectively. In addition to two doublet signals at δ_H_ = 7.27 (*J* = 6.6 Hz) and 7.15 ppm (*J* = 9.0 Hz), which assigned as quinolinone-H-7,7′,8,8’. Furthermore, the ^13^C NMR spectrum of compound 5b revealed the following signals at δ_C_ = 162.87, 160.98, 157.25, 45.38, 35.88, and 20.75 ppm, which were assigned as C=O, C=N, quinolinone-C-4,4′, piperazine-CH_2_, cyclopentyl-CH, and methyl groups, respectively (Figure 5).

**SCHEME 3:**
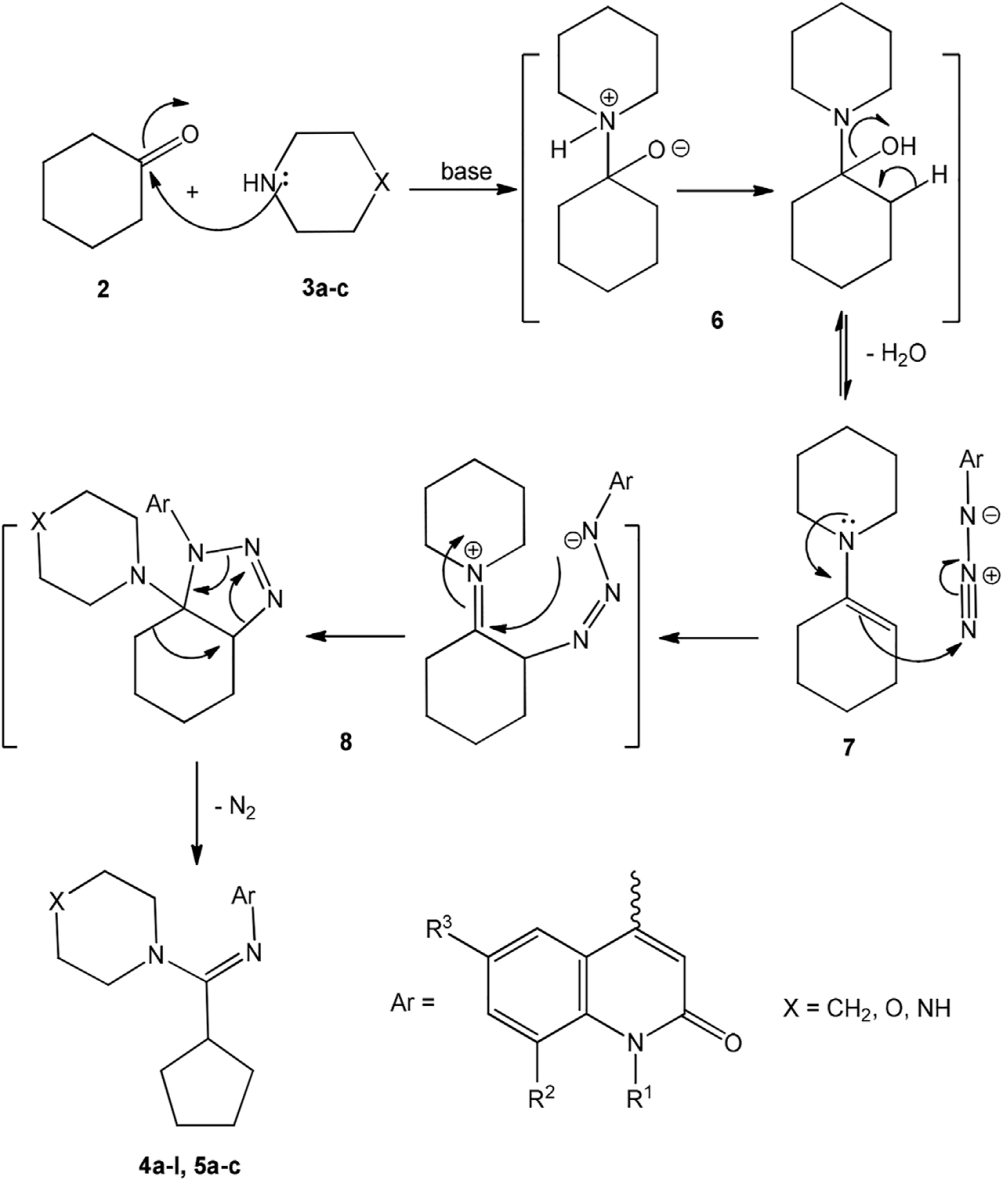
Formation of amidines **4a-l, 5a-c**.

The formation of products amidines 4a-l and 5a-c can be rationalized by the following mechanism. In the first step, cyclohexanone (2) reacted with secondary amine 3a-c catalyzed with excess base and resulted in the formation of enamine 7. The formed enamine 7 is in equilibrium with its initial cyclohexanone and secondary amine, which facilitates the second step. The second step is an interaction between enamine 7 and our 4-azidoquinolinones 1a-f catalyzed by an excess of base and forming triazoline 8, which loss a molecule of nitrogen to give the desirable amidines 4a-l and 5a-c as shown in Scheme 3.

### 2.2 Biology

#### 2.2.1 Antiproliferative action

##### 2.2.1.1 Cell viability assay

To study the effect of **4a-l** and **5a-c** on normal cell lines, a cell viability assay was done using the MCF-10A (human mammary gland epithelial) cell line (Abdelbaset et al., 2019; Al-Wahaibi et al., 2020). This study uses a concentration of 50 µM of the investigated compound for 4 days, after which cell viability is evaluated. Table 1 shows that compounds **4a-l** and **5a-c** have no toxic impact and have greater than 85% cell viability.

**TABLE 1:** IC_50_ of compounds 4a-l, 5a-c, and Doxorubicin.

Compd. No.	Cell viability	Antiproliferative activity IC_50_ ± SEM (μM)				
	(50 µM)	Panc-1	MCF-7	HT-29	A-549	Average
4a	85	5.40 ± 0.50	5.10 ± 0.50	5.50 ± 0.50	5.90 ± 0.50	5.50
4b	92	1.50 ± 0.10	1.40 ± 0.10	1.60 ± 0.10	1.50 ± 0.10	1.50
4c	91	2.05 ± 0.20	1.90 ± 0.20	2.20 ± 0.20	2.30 ± 0.20	2.10
4d	92	1.30 ± 0.10	1.20 ± 0.10	1.40 ± 0.10	1.50 ± 0.10	1.35
4e	90	1.20 ± 0.10	1.10 ± 0.10	1.20 ± 0.10	1.30 ± 0.10	1.20
4f	87	7.90 ± 0.80	7.50 ± 0.80	7.70 ± 0.80	8.00 ± 0.80	7.80
4g	89	6.10 ± 0.60	5.90 ± 0.60	6.20 ± 0.60	6.30 ± 0.60	6.10
4h	91	2.30 ± 0.20	1.90 ± 0.20	2.30 ± 0.20	2.60 ± 0.20	2.30
4i	89	1.60 ± 0.20	1.40 ± 0.20	1.20 ± 0.20	1.10 ± 0.20	1.30
4j	89	3.10 ± 0.30	2.90 ± 0.30	3.50 ± 0.30	3.70 ± 0.30	3.30
4k	91	4.20 ± 0.40	4.05 ± 0.40	4.10 ± 0.40	4.60 ± 0.40	4.20
4l	89	7.40 ± 0.70	7.10 ± 0.70	7.20 ± 0.70	7.60 ± 0.70	7.30
5a	91	4.90 ± 0.30	4.70 ± 0.40	5.10 ± 0.30	5.15 ± 0.30	5.00
5b	91	3.90 ± 0.30	3.70 ± 0.30	3.80 ± 0.30	4.10 ± 0.30	3.90
5c	89	0.90 ± 0.10	0.80 ± 0.10	1.10 ± 0.10	1.10 ± 0.10	1.00
Doxorubicin	--	1.40 ± 0.20	0.90 ± 0.10	1.00 ± 0.10	1.20 ± 0.10	1.10

##### 2.2.1.2 Antiproliferative assay

The newly synthesized compounds were investigated for antiproliferative activity against four different types of cancer cells (Marzouk et al., 2020; Mohassab et al., 2021): A-549 (epithelial cancer cell line), MCF-7 (breast cancer cell line), Panc-1 (pancreas cancer cell line), and HT-29 (colon cancer cell line). Table 1 shows the results of calculating the IC_50_ of each compound using doxorubicin as the reference. In general, the newly examined compounds 4a-l and 5a-c showed considerable antiproliferative activity against the four tested cancer cell lines, with mean GI_50_ ranging from 1 μM to 7.80 µM compared to the reference doxorubicin, which had a GI_50_ of 1.10 µM. The highest antiproliferative activity was found in five compounds: 4b, 4d, 4e, 4i (Scaffold A), and 5c (Scaffold B), with GI_50_ values ranging from 1.00 µM to 1.50 µM.

Compound 5c (R^1^ = CH_3_, R^2^ = R^3^ = H, Scaffold B) was the most potent derivative with a GI50 of 1.00 µM, equivalent to the reference doxorubicin’s GI_50_ of 1.10 µM. Except for HT-29 (colon cancer cell line), compound 5c suppressed all cancer cell lines more efficiently than doxorubicin.

The Scaffold B compounds 5a (R^1^ = R^2^ = R^3^ = H) and 5b (R^1^ = R^2^ = H, R^3^ = CH_3_) both exhibited modest antiproliferative activity with GI_50_ values of 5.00 µM and 3.90 µM, respectively.

Compared to doxorubicin, which has a GI_50_ of 1.10 µM, compounds 4b, 4d, 4e, and 4i, which are based on Scaffold A, demonstrated strong antiproliferative activity with GI_50_ ranging from 1.20 µM to 1.50 µM. With a GI_50_ of 1.20 µM, compound 4e (R^1^ = R^3^ = H, R^2^ = Me, X = CH_2_) was equal to and even more effective than doxorubicin against the Panc-1 (pancreas cancer) cell line, placing it second in activity after compound 5a.

Compound 4i (R^1^ = R^2^ = H, R^3^ = OCH_3_, X = O) demonstrated a potent antiproliferative effect with a GI_50_ of 1.30 µM, indicating that scaffold-A-based compounds were more potent as antiproliferative agents against cancer cell lines than scaffold B compounds 5a and 5b and that there was no significant difference between using piperidine or morpholine as the amine part in the formation of such class of organic compounds.

Compounds 4g (R^1^ = R^2^ = R^3^ = H, X = O) and 4l (R^1^ = Me, R^2^ = R^3^ = H, X = O) were the least potent derivatives, with GI_50_ values of 6.10 µM and 7.30 µM, respectively, being at least 6-fold less potent than the reference doxorubicin (Table 1).

#### 2.2.2 Topo I and IIα inhibitory activity

Topoisomerase I inhibitors are a unique class of anticancer agents that cause cell death by interfering with DNA replication in cancer cells (Delgado et al., 2018). According to the literature, quinoline-based derivatives exhibited an antiproliferative impact by inhibiting variable proteins such as topo I and topo II (Monsalve et al., 2012; Ismail et al., 2013).

Topo I and IIα inhibitory activity of the most active compounds 4b, 4d, 4e, 4i, and 5c were determined at 100 μM and 20 µM concentrations using Camptothecin as a positive reference for topo I inhibitory test and Etoposide for topo IIα inhibitory assay (Park et al., 2019). The results are summarized in Table 2.

**TABLE 2:** Topo I and IIα inhibitory activity of compounds 4b, 4d, 4e, 4i, and 5c.

Code no.	Topo I (% inhibition)		Topo IIα (% inhibition)	
	100 µM	20 µM	100 µM	20 µM
4b	65.9	29.6	57.2	22.30
4d	71.6	34.7	54.2	21.70
4e	81.4	39.1	60.4	25.60
4i	74.9	37.20	56.70	23.10
5c	84.8	46.7	23.8	ND
Camptothecin	82.6	40	ND	ND
Etoposide	ND	ND	83.7	66

Most of the investigated compounds displayed mild to moderate topo IIα inhibitory action at 100 µM (23.8%–60.4%) compared to the standard etoposide (83.7%). Compound **4e** (R^1^ = R^3^ = H, R^2^ = Me, X = CH_2_, Scaffold A) had the best topo IIα inhibitory action at both concentrations (100 µM = 60.40% and 20 µM = 25.60%). Compounds **4b**, **4d**, and **4i** showed similar topo IIα inhibitory activity at 100 μM, ranging from 54.2% to 57.2% (Table 2). The most effective antiproliferative agent, compound **5c**, had the lowest activity against topo IIα.

Compared to the reference drug Camptothecin (82.6%), most evaluated compounds showed selective topo I inhibitory action ranging from 65.9% to 84.8% at 100 µM. The most effective derivative in the antiproliferative assay test, 5c, demonstrated higher topo I inhibitory activity (84.8) than the reference camptothecin (82.6%) at 100 µM. Compound 5c may eventually be developed as a viable anticancer agent because it was discovered to be effective at suppressing Topo I and to have strong antiproliferative action.

The most significant topo I inhibition was demonstrated by compounds 4e and 4i after compound 5c (81.4% and 74.9%, respectively). However, their activity was lower than that of the standard drug camptothecin (82.6%). As a result, we can infer that Topo I inhibition is essential to the antiproliferative effect of these compounds.

### 2.3 Molecular docking simulations

Based on promising antiproliferative activity shown by test compounds (4a-l and 5a-c) against 4 cancer lines and inhibitory activity exerted on Topo I and Topo IIα enzymes, we run molecular docking simulations to explore the possible mode of inhibition of such class of compounds. Docking simulations within the active site of Topo I revealed an interesting binding profile that could be used as an explanation for their inhibitory activity. As shown in Table 3, docking scores of test compounds were moderate to strong, especially compounds 4i and 5c which showed the highest docking score compared to co-crystallized ligand, Camptothecin (CPT).

**TABLE 3:** Docking Score and Binding Interactions of 4b, 4d, 4e, 4i and 5c within active sites of Topo I and Topo IIα proteins (PDB ID: 1T8I and 4FM9; respectively).

Cpd	Topo I (PDB ID: 1T8I)					Topo IIα (PDB ID: 4FM9)				
	S (Kcal/mol)	RMSD (Å)	Binding interactions			S	RMSD (Å)	Binding interactions		
a.a. residue	Type	Distance (Å)	a.a. residue	Type	Distance
4b	−5.05	2.28	DT10	pi-pi	3.97	−5.11	2.07	-	-	-
			TGP11	pi-H	3.59					
			THR718	H-donor	3.15					
4d	−5.12	2.13	THR718	H-donor	3.15	−5.05	2.08	-	-	-
4e	−5.62	1.26	ARG364	H-acceptor	3.21	−5.90	2.23	GLU682	H-donor	3.27
			DA113	pi-pi	3.56					
4i	−6.26	1.38	ARG364	H-acceptor	2.82	−5.15	1.78	-	-	-
5c	−8.19	1.36	ARG364	pi-H	4.12	NA	NA	-	-	-
Ref^b^	−7.30	1.28	ARG364	H-acceptor	2.91	−6.5	2.26	LEU592	H-acceptor	2.9
			TGP11	pi-pi	3.64					
			DA113	pi-pi	3.63					
			DA113	pi-pi	3.52					
			TGP11	pi-pi	3.67					
			DA113	pi-pi	3.48					
			DC112	pi-pi	3.60					

^a^ S: docking score (Kcal/mol).

^b^ Ref: co-crystallized ligand Camptothecin (CPT) for 1T8I, Etoposide for 4FM9.

**FIGURE 6:**
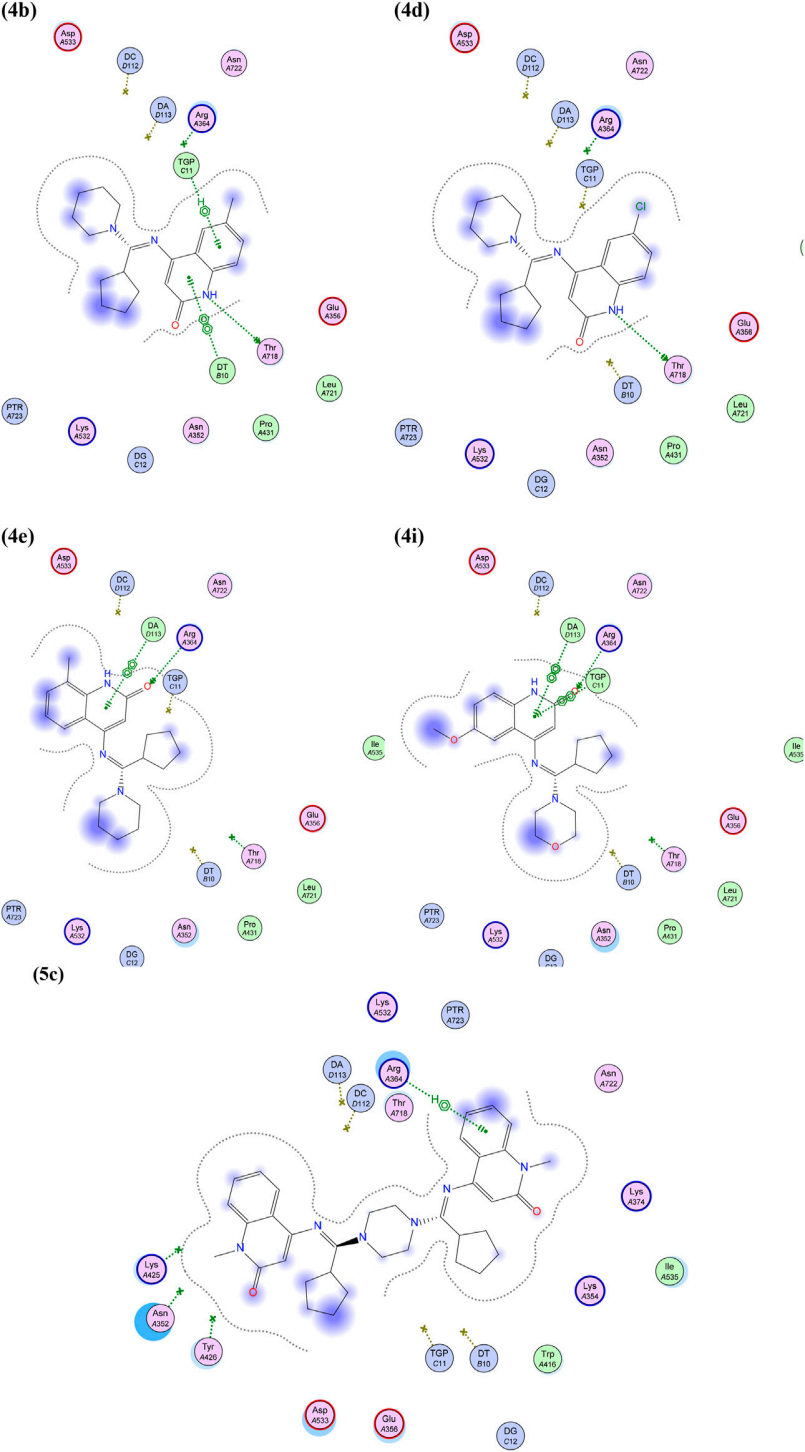
Best poses of 4b, 4d, 4e, 4i, and 5c into the DNA-Topo I complex, showing H-bonds (as a green arrow) and both pi-H and pi-pi stacking interactions (as green-dotted lines).

Visual inspection of stacking interactions especially those mediated through either H-bond and/or pi-H interaction with Arg 364, were found to be with compounds 4e, 4i, and 5c (which is one of the conserved intercalating binding interactions within the minor groove side of Topo I seen with three well-known Topo I poisons; camptothecin and representative members of the indolocarbazole and indinoisoquinoline classes) (Staker et al., 2005; Lauria et al., 2021), in addition to a number of H-bond with THR 718 and/or TGP11 and DT10 base pairs, as shown in Figure 6.

**FIGURE 7:**
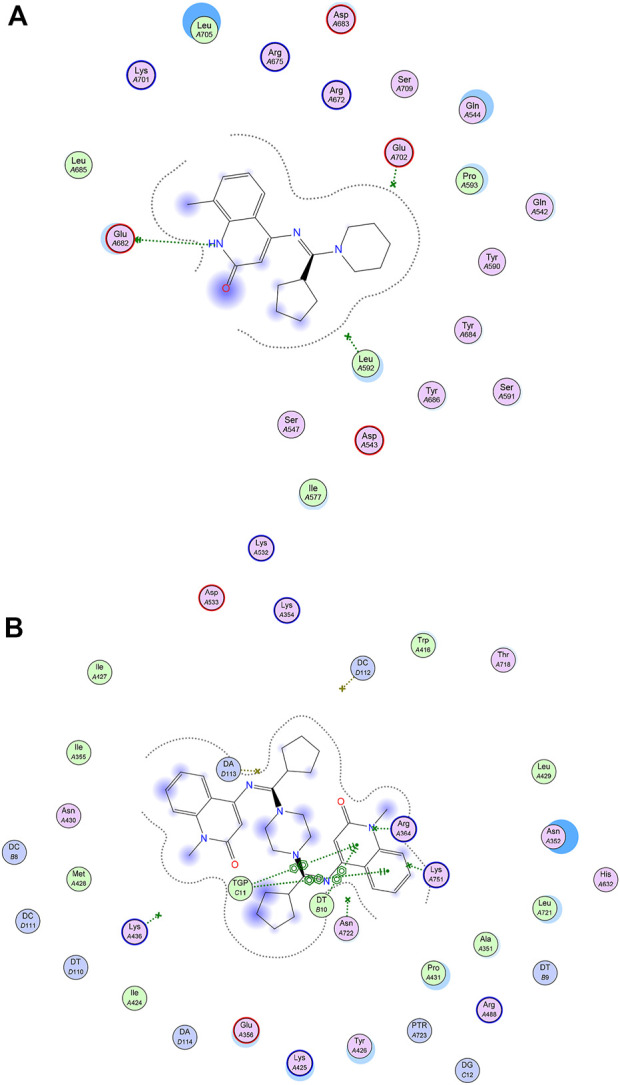
**(A)** Binding mode of 4e in active site of Topo IIα (PDB ID: 4FM9); **(B)** Binding mode of 5c in DNA-Topo I complex (PDB ID: 1T8I).

Additionally, as we mentioned before that the majority of the investigated compounds displayed mild to moderate topo IIα inhibitory activity, so we also explored their binding behavior within its crystal structure (RCSB data bank deposited crystal structure; PDB ID: 4FM9) and results (as docking score and RMSD were listed in Table 3), and interestingly we found a very close docking profile with all these test compounds represented by having a medium docking score (S = 5.05–5.90 kcal/mol, c.f. Etoposide: 6.50 kcal/mol). Based on the inhibitory activity of these compounds against the Topo IIα enzyme, compound 4e has the best % inhibition among its congeners (4b, 4d, and 4i). From our interest in exploring its binding interactions within the Topo IIα active site, we examined its best docking poses obtained in Figure 7.

Another interesting observation was seen during docking simulations of such class of compounds that changing the configuration from found *E*-form to *Z*-form results in enhancement in docking score with a different binding interactions with various amino acid residues lining active site of Topo I. taking compound 5c (which has the highest docking score) to examine its docking poses hoping to explain such higher score, we found the appearance of multiple stacking pi-pi interactions with DT10 and TGP11 base pairs which contribute to stabilization of its molecule within active site, as shown in Figure 7B. To conclude, such new synthesized quinoline-derivatives (either *E*- or *Z*-form) have a promising binding profile with both Topo I and Topo IIα active sites comparable to co-crystallized ligands (Camptothecin and Etoposide; respectively), and further investigation of best derivatives is worth mentioning.

## 3 Conclusion

A new series of amidines (**4a-l** and **5a-c**) was synthesised and their structures were determined using ^1^H NMR, ^13^C NMR, and elemental analyses. The new compounds were tested against four cancer cell lines as antiproliferative agents. The majority of the synthesised derivatives showed promising inhibitory activity, with GI_50_ values ranging from 1.00 µM to 7.80 µM. The most active derivatives as antiproliferative agents inhibited Topo I better than Topo IIα, as xplained by docking studies. The new synthesized quinoline-derivatives have a promising binding profile with both Topo I and Topo IIα active sites comparable to co-crystallized ligands (Camptothecin and Etoposide.

## 4 Experimental

### 4.1 Chemistry

**General Details:** See Supplementary Appendix S1A.

4-Azdo-quinoline-2(1*H*)-ones **1a-f** were prepared according to reported procedures (Derin et *al.,* 2008; Steinschifter et al., 1994; Stadlbauer et al., 1991).

#### 4.1.1 General procedure for the synthesis of compounds (4a-l)

In a round-bottom flask, mix 1.5 mmol of cyclohexanone (**2**) and 2 mmol of piperidine (**3a**) or morpholine (**3b**) in 25 ml of DMF as a solvent with stirring at 60°C for 1h. Then, 1 mmol of 4-azido-quinoline-2(1*H*)-ones **1a-f** was added in one portion to the above mixture. The reaction mixture was stirred at the same temperature for another 13 h, and the reaction was followed with TLC. After completion, the reaction mixture was poured into a conical containing 200 g crashed ice with stirring. The obtained products were filtered under vacuum, washed three times with cooled water, and dried well to give products **4a-l** in good yields.

##### 4.1.1.1 (*E*) 4-((Cyclopentyl(piperidin-1-yl)methylene)amino)quinolin-2(1*H*)-one (4a)

Colorless powder, (90%), m.p. 210°C; ^1^H NMR (DMSO-d_6_): δ_H_ = 11.10 (bs; 1H, NH-1), 7.45 (m; 2H, quinolin-H), 7.25 (d, *J* = 8.1 Hz; 1H, quinolin-H), 7.08 (t, *J* = 7.5 Hz; 1H, quinolin-H), 5.39 (bs; 1H, H-3), 3.41 (m; 10H, piperidinyl-H), 2.94 (m; 1H, H-a'), 1.70–1.46 ppm (m; 8H, cyclopentyl-CH_2_), ^13^C NMR (DMSO-d_6_): δ_C_ = 162.06 (C=O), 160.49 (C=N), 157.72 (C-4), 139.05 (C-8a), 130.25 (C-7), 124.37 (C-5), 121.96 (C-6), 117.77 (C-3), 115.19 (C-8), 103.85 (C-4a), 46.63 (C-a), 40.33 (C-a'), 30.55 (C-b'), 25.83 (C-b), 25.45 (C-c'), 24.14 ppm (C-c). *Anal. Calcd for* C_20_H_25_N_3_O: C, 74.27; H, 7.79; N, 12.99; Found: C, 74.50; H, 7.87; N, 13.20.

##### 4.1.1.2 (*E*) 4-((Cyclopentyl(piperidin-1-yl)methylene)amino)-6-methylquinolin-2(1*H*)-one (4b)

Colorless powder, (85%), m.p. 222°C; ^1^H NMR (DMSO-d_6_): δ_H_ = 11.06 (bs; 1H, NH-1), 7.26–7.24 (m; 2H, quinolin-H), 7.12 (d, *J* = 7.8 Hz; 1H, quinolin-H), 5.36 (bs; 1H, H-3), 3.40 (m; 10H, piperidinyl-H), 2.96 (m; 1H, H-a'), 2.29 (s; 3H, Me), 1.70–1.46 ppm (m; 8H, cyclopentyl-CH_2_), ^13^C NMR (DMSO-d_6_): δ_C_ = 162.95 (C=O), 160.56 (C=N), 157.48 (C-4), 137.02 (C-8a), 131.37 (C-7), 129.67 (C-6), 123.85 (C-5), 117.64 (C-3), 115.15 (C-8), 103.79 (C-4a), 46.62 (C-a), 40.60 (C-a'), 30.58 (C-b'), 25.18 (C-b), 25.45 (C-c'), 24.11 (C-c), 20.68 ppm (Me). EI-MS (m/z, %): 337 (M^+^, 55). *Anal. Calcd for* C_21_H_27_N_3_O: C, 74.74; H, 8.06; N, 12.45; Found: C, 74.61; H, 8.23; N, 12.71.

##### 4.1.1.3 (*E*) 4-((Cyclopentyl(piperidin-1-yl)methylene)amino)-6-methoxyquinolin-2(1*H*)-one (4c)

Colorless powder, (82%), m.p. 250°C; ^1^H NMR (DMSO-d_6_): δ_H_ = 11.01 (bs; 1H, NH-1), 7.23–7.09 (m; 2H, quinolin-H), 6.90 (d, *J* = 2.1 Hz; 1H, quinolin-H), 5.41 (bs; 1H, H-3), 3.73 (s; 3H, OMe), 3.42–3.31 (m; 10H, piperidinyl-H), 2.97 (m; 1H, H-a'), 1.72–1.49 ppm (m; 8H, cyclopentyl-CH_2_), ^13^C NMR (DMSO-d_6_): δ_C_ = 162.65 (C=O), 160.56 (C=N), 157.15 (C-4), 153.55 (C-6), 133.44 (C-8a), 133.44 (C-7), 118.69 (C-3), 118.29 (C-8), 116.49 (C-4a), 106.27 (C-5), 55.19 (OMe), 46.66 (C-a), 40.61 (C-a'), 30.54 (C-b'), 25.78 (C-b), 25.48 (C-c'), 24.12 ppm (C-c). *Anal. Calcd for* C_21_H_27_N_3_O_2_: C, 71.36; H, 7.70; N, 11.89; Found: C, 71.52; H, 7.88; N, 12.13.

##### 4.1.1.4 (*E*) 4-((Cyclopentyl(piperidin-1-yl)methylene)amino)-6-chloroquinolin-2(1*H*)-one (4d)

Colorless powder, (75%), m.p. 248°C; ^1^H NMR (DMSO-d_6_): δ_H_ = 11.27 (bs; 1H, NH-1), 7.48–7.64 (t, *J* = 6, 2.5 Hz; 1H, quinolin-H), 7.41 (s; 1H, quinolin), 7.25–7.23 (d, *J* = 9 Hz; 1H, quinolin-H), 5.39 (bs; 1H, H-3), 3.42–3.34 (m; 10H, piperidinyl-H), 3.01 (m; 1H, H-a'), 1.71–1.47 ppm (m; 8H, cyclopentyl-CH_2_), ^13^C NMR (DMSO-d_6_): δ_C_ = 162.83 (C=O), 161.39 (C=N), 156.33 (C-4), 137.80 (C-8a), 130.13 (C-7), 124.91 (C-8), 123.25 (C-6), 119.25 (C-3), 117.17 (C-4a), 104.34 (C-5), 46.69 (C-a), 40.43 (C-a'), 30.64 (C-b'), 25.85 (C-b), 25.47 (C-c'), 24.06 ppm (C-c). *Anal. Calcd for* C_20_H_24_ClN_3_O: C, 67.12; H, 6.76; N, 11.74; Found: C, 67.40; H, 6.89; N, 12.01.

##### 4.1.1.5 (*E*) 4-((Cyclopentyl(piperidin-1-yl)methylene)amino)-8-methylquinolin-2(1*H*)-one (4e)

Pale yellow precipitate, (88%), m.p. 184°C; ^1^H NMR (DMSO-d_6_): δ_H_ = 10.25 (bs; 1H, NH-1), 7.36–7.27 (m; 2H, quinolin-H), 6.99 (t, *J* = 7.5 Hz; 1H, quinolin-H), 5.42 (bs; 1H, H-3), 3.40–3.32 (m; 10H, piperidinyl-H), 2.95 (m; 1H, H-a'), 2.40 (s; 3H, Me), 1.67–1.46 ppm (m; 8H, cyclopentyl-CH_2_), ^13^C NMR (DMSO-d_6_): δ_C_ = 163.28 (C=O), 160.32 (C=N), 158.17 (C-4), 137.36 (C-8a), 131.52 (C-7), 123.26 (C-6), 122.42 (C-5), 120.65 (C-8), 117.81 (C-3), 103.56 (C-4a), 46.62 (C-a), 40.66 (C-a'), 30.52 (C-b'), 25.78 (C-b), 25.43 (C-c'), 24.11 (C-c), 17.43 ppm (Me). *Anal. Calcd for* C_21_H_27_N_3_O: C, 74.74; H, 8.06; N, 12.45; Found: C, 74.85; H, 8.22; N, 12.71.

##### 4.1.1.6 (*E*) 4-((Cyclopentyl(piperidin-1-yl)methylene)amino)-1-methylquinolin-2(1*H*)-one (4f)

Colorless precipitate, (77%), m.p. 142°C; ^1^H NMR (DMSO-d_6_): δ_H_ = 7.59–7.56 (m; 1H, quinolin-H), 7.41 (d, *J* = 8.4 Hz; 1H, quinolin-H), 7.18 (t, *J* = 7.5 Hz; 1H, quinolin-H), 5.54 (bs; 1H, H-3), 3.54 (s; 3H, Me), 3.41–3.32 (m; 10H, piperidinyl-H), 2.96 (m; 1H, H-a'), 1.69–1.44 ppm (m; 8H, cyclopentyl-CH_2_), ^13^C NMR (DMSO-d_6_): δ_C_ = 162.03 (C=O), 160.66 (C=N), 156.30 (C-4), 139.83 (C-8a), 130.64 (C-7), 124.93 (C-6), 121.03 (C-5), 118.85 (C-3), 114.45 (C-8), 103.41 (C-4a), 46.60 (C-a), 40.53 (C-a'), 30.51 (C-b'), 28.38 (C-b), 25.77 (C-c'), 25.42 (C-c), 24.08 ppm (Me). *Anal. Calcd for* C_21_H_27_N_3_O: C, 74.74; H, 8.06; N, 12.45; Found: C, 74.91; H, 8.23; N, 12.62.

##### 4.1.1.7 (*E*) 4-((Cyclopentyl(morpholino)methylene)amino)quinolin-2(1*H*)-one (4g)

Colorless powder, (89%), m.p. 249°C; ^1^H NMR (DMSO-d_6_): δ_H_ = 11.16 (bs; 1H, NH-1), 7.42 (dd, *J* = 7.8, 1.2 Hz; 2H, quinolin-H), 7.24, 7.09 (m; 2H, quinolin-H), 5.46 (bs; 1H, H-3), 3.65 (t, *J* = 5.1 Hz; 4H, H-b), 3.44 (t, *J* = 5.1 Hz; 4H, H-a), 2.96 (m; 1H, H-a'), 1.69–1.45 ppm (m; 8H, cyclopentyl-CH_2_), ^13^C NMR (DMSO-d_6_): δ_C_ = 162.93 (C=O), 160.86 (C=N), 157.45 (C-4), 139.01 (C-8a), 130.39 (C-7), 124.36 (C-5), 121.07 (C-6), 117.47 (C-3), 115.23 (C-8), 104.40 (C-4a), 65.95 (C-b), 46.19 (C-a), 40.27 (C-a'), 30.39 (C-b'), 25.79 ppm (C-c'). EI-MS (m/z, %): 325 (M^+^, 48). *Anal. Calcd for* C_19_H_23_N_3_O_2_: C, 70.13; H, 7.12; N, 12.91; Found: C, 70.39; H, 7.30; N, 13.08.

##### 4.1.1.8 (*E*) 4-((Cyclopentyl(morpholino)methylene)amino)-6-methylquinolin-2(1*H*)-one (4h)

Colorless powder, (87%), m.p. 226°C; ^1^H NMR (DMSO-d_6_): δ_H_ = 11.06 (bs; 1H, NH-1), 7.23 (m; 3H, quinolin-H), 5.43 (bs; 1H, H-3), 3.65 (t, *J* = 4.5 Hz; 4H, H-b), 3.45 (t, *J* = 4.2 Hz; 4H, H-a), 2.96 (m; 1H, H-a'), 2.30 (s; 3H, Me), 1.71–1.45 ppm (m; 8H, cyclopentyl-CH_2_), ^13^C NMR (DMSO-**d**_**6**_): δ_C_ = 162.78 (C=O), 160.89 (C=N), 157.17 (C-4), 136.97 (C-8a), 131.51 (C-7), 129.83 (C-5), 123.78 (C-6), 117.33 (C-3), 115.16 (C-8), 104.34 (C-4a), 65.92 (C-b), 46.18 (C-a), 40.26 (C-a'), 30.41 (C-b'), 25.75 (C-c'), 20.64 ppm (Me). *Anal. Calcd for* C_20_H_25_N_3_O_2_: C, 70.77; H, 7.42; N, 12.38; Found: C, 70.98; H, 7.61; N, 12.59.

##### 4.1.1.9 (*E*) 4-((Cyclopentyl(morpholino)methylene)amino)-6-methoxyquinolin-2(1*H*)-one (4i)

Colorless powder, (82%), m.p. 247°C; ^1^H NMR (DMSO-d_6_): δ_H_ = 11.06 (bs; 1H, NH-1), 7.23–7.10 (m; 2H, quinolin-H), 6.87 (d, *J* = 2.7 Hz; 1H, quinolin-H), 5.47 (bs; 1H, H-3), 3.37 (s; 3H, OMe), 3.65 (t, *J* = 4.8 Hz; 4H, H-b), 3.45 (t, *J* = 4.5 Hz; 4H, H-a), 2.96 (m; 1H, H-a'), 1.70–1.46 ppm (m; 8H, cyclopentyl-CH_2_), ^13^C NMR (DMSO-d_6_): δ_C_ = 162.55 (C=O), 161.00 (C=N), 156.91 (C-4), 153.63 (C-6), 133.39 (C-8a), 118.82 (C-4a), 118.02 (C-7), 116.**56** (C-3), 106.56 (C-5), 104.77 (C-4a), 65.95 (C-b), 55.26 (OMe), 46.20 (C-a), 40.27 (C-a'), 30.40 (C-b'), 25.77 ppm (C-c'). *Anal. Calcd for* C_20_H_25_N_3_O_3_: C, 67.58; H, 7.09; N, 11.82; Found: C, 67.71; H, 7.23; N, 12.04.

##### 4.1.1.10 (*E*) 4-((Cyclopentyl(morpholino)methylene)amino)-6-chloroquinolin-2(1*H*)-one (4j)

Colorless powder, (73%), m.p. 247°C; ^1^H NMR (DMSO-d_6_): δ_H_ = 11.32 (bs; 1H, NH-1), 7.5–7.47 (m; 1H, quinolin-H), 7.42–7.41 (d, *J* = 0.8 Hz; 1H, quinolin), 7.26–7.24 (d, *J* = 9 Hz; 1H, quinolin-H), 5.46 (bs; 1H, H-3), 3.65–363 (t, *J* = 4 Hz; 4H, H-b), 3.64–3.45 (t, *J* = 4.5 Hz; 4H, H-a), 2.99 (m; 1H, H-a'), 1.73–1.46 ppm (m; 8H, cyclopentyl-CH_2_), ^13^C NMR (DMSO-d_6_): δ_C_ = 162.68 (C=O), 161.66 (C=N), 156.12 (C-4), 137.78 (C-8a), 130.30 (C-7), 125.03 (C-8), 123.29 (C-5), 118.96 (C-6), 117.20 (C-3), 105.02 (C-4a), 65.92 (C-b), 46.18 (C-a), 39.50 (C-a'), 30.49 (C-b'), 25.82 ppm (C-c'). *Anal. Calcd for* C_19_H_22_ClN_3_O_2_: C, 63.42; H, 6.16; N, 11.68; Found: C, 63.70; H, 6.39; N, 11.91.

##### 4.1.1.11 (*E*) 4-((Cyclopentyl(morpholino)methylene)amino)-8-methylquinolin-2(1*H*)-one (4k)

Colorless powder, (81%), m.p. 220°C; ^1^H NMR (DMSO-d_6_): δ_H_ = 10.32 (bs; 1H, NH-1), 7.34–7.27 (m; 2H, quinolin-H), 7.04 (t, *J* = 7.5 Hz; 1H, quinolin-H), 5.48 (bs; 1H, H-3), 3.63 (t, *J* = 4.5 Hz; 4H, H-b), 3.44 (t, *J* = 4.2 Hz; 4H, H-a), 2.97 (m; 1H, H-a'), 2.40 (s; 3H, Me), 1.70–1.45 ppm (m; 8H, cyclopentyl-CH_2_), ^13^C NMR (DMSO-d_6_): δ_C_ = 163.17 (C=O), 160.69 (C=N), 157.89 (C-4), 137.36 (C-8a), 131.66 (C-7), 123.34 (C-6), 122.41 (C-5), 120.76 (C-8), 117.52 (C-3), 104.14 (C-4a), 65.95 (C-b), 46.20 (C-a), 40.36 (C-a'), 30.39 (C-b'), 25.75 (C-c'), 17.43 ppm (Me). *Anal. Calcd for* C_20_H_25_N_3_O_2_: C, 70.77; H, 7.42; N, 12.38; Found: C, 70.98; H, 7.60; N, 12.47.

##### 4.1.1.12 (*E*) 4-((Cyclopentyl(morpholino)methylene)amino)-1-methylquinolin-2(1*H*)-one (4l)

Colorless powder, (76%), m.p. 213°C; ^1^H NMR (DMSO-d_6_): δ_H_ = 7.65–7.44 (m; 3H, quinolin-H), 7.20 (t, *J* = 7.5 Hz; 1H, quinolin-H), 5.60 (bs; 1H, H-3), 3.64 (t; 4H, H-b), 3.55 (s; 3H, Me), 3.46 (t; 4H, H-a), 2.98 (m; 1H, H-a'), 1.71–1.46 ppm (m; 8H, cyclopentyl-CH_2_), ^13^C NMR (DMSO-d_6_): δ_C_ = 161.96 (C=O), 161.09 (C=N), 156.10 (C-4), 139.81 (C-8a), 130.85 (C-7), 124.96 (C-6), 121.22 (C-5), 118.57 (C-3), 114.61 (C-8), 104.02 (C-4a), 65.94 (C-b), 46.18 (C-a), 40.34 (C-a'), 30.38 (C-b'), 28.50 (Me), 25.79 ppm (C-c'). *Anal. Calcd for* C_20_H_25_N_3_O_2_: C, 70.77; H, 7.42; N, 12.38; Found: C, 70.95; H, 7.58; N, 12.63.

#### 4.1.2 General procedure for the synthesis of compounds (5a-c)

In a round-bottom flask, mix 3 mmol of cyclohexanone (**2**) and 2 mmol of piperazine (**3c**) in 25 ml of DMF as a solvent with stirring at 60°C for 1 h. Then, 1 mmol of 4-azido-quinoline-2(1*H*)-ones **1a-c** was added in one portion to the above mixture. The reaction mixture was stirred at the same temperature for another 24 h, and the reaction mixture was followed with TLC. After completion, the reaction mixture was poured into a conical containing 200 g crashed ice with stirring. The obtained products were filtered under vacuum, washed three times with cooled water, and dried well to give products **5a-c** in good yields.

##### 4.1.2.1 4,4'-((1*E*,1′*E*)-(piperazine-1,4-diylbis(cyclopentylmethanylylidene))bis(azanylylid-ene))bis(quinolin-2(1*H*)-one) (5a)

Colorless powder, (89%), m.p. 360°C; ^1^H NMR (DMSO-d_6_): δ_H_ = 11.08 (bs; 2H, NH-1, NH-1′), 7.27 (dd, *J* = 4.5, 2.1 Hz; 4H, quinolin-H), 7.16 (t, *J* = 4.5 Hz; 4H, quinolin-H), 5.45 (bs; 2H, H-3,3′), 3.59 (b; 8H, piperazine-H), 2.49 (m; 1H, cyclopentyl-CH), 1.74–1.47 ppm (m; 16H, cyclopentyl-CH_2_), ^13^C NMR (DMSO-d_6_): δ_C_ = 162.84 (2CO), 160.91 (C=N), 157.12 (C-4,4′), 137.04 (C-8a, 8a′), 131.55 (C-7,7′), 129.87 (C-5,5′), 123.91 (C-6,6′), 117.44 (C-3,3′), 115.21 (C-8,8′), 104.45 (C-4a, 4a’), 45.36 (piperazine-CH_2_), 38.70 (cyclopentyl-CH), 30.70, 25.83 ppm (cyclopentyl-CH_2_). EI-MS (m/z, %): 562 (M^+^, 30). *Anal. Calcd for* C_34_H_38_N_6_O_2_: C, 72.57; H, 6.81; N, 14.94; Found: C, 72.80; H, 7.04; N, 15.12.

##### 4.1.2.2 4,4'-((1*E*,1′*E*)-(piperazine-1,4-diylbis(cyclopentylmethanylylidene))bis(azanylylid-ene))bis(6-methylquinolin-2(1*H*)-one) (5b)

Colorless powder, (84%), m.p. 355°C; ^1^H NMR (DMSO-d_6_): δ_H_ = 11.06 (bs; 2H, NH-1, NH-1′), 7.95 (s; 2H, quinolin-H-5,5′), 7.27 (d, *J* = 6.6 Hz; 2H, quinolin-H), 7.15 (d, *J* = 9.0 Hz; 2H, quinolin-H), 5.45 (bs; 2H, H-3,3′), 3.60 (bs; 8H, piperazine-H), 2.97 (m; 1H, cyclopentyl-CH), 2.73 (s; 3H, Me), 1.74–1.47 ppm (m; 16H, cyclopentyl-CH_2_), ^13^C NMR (DMSO-d_6_): δ_C_ = 162.87 (CO), 160.98 (C=N), 157.25 (C-4,4′), 137.07 (C-6,6′), 131.63 (C-8,8a′), 129.93 (C-5,5′), 123.95 (C-7,7′), 117.47 (C-3,3′), 115.26 (C-8,8′), 104.51 (C-4a, 4a’), 45.38 (piperazine-CH_2_), 35.88 (cyclopentyl-CH), 30.59, 25.88 (cyclopentyl-CH_2_), 20.75 ppm (Me). *Anal. Calcd for* C_36_H_42_N_6_O_2_: C, 73.19; H, 7.17; N, 14.23; Found: C, 73.41; H, 7.35; N, 14.50.

##### 4.1.2.3 4,4'-((1*E*,1′*E*)-(piperazine-1,4-diylbis(cyclopentylmethanylylidene))bis(azanylylid-ene))bis(1-methylquinolin-2(1H)-one) (5c)

Colorless powder, (77%), m.p. 342°C; ^1^H NMR (DMSO-d_6_): δ_H_ = 7.62–719 (m; 8H, quinolin-H), 5.61 (bs; 2H, H-3,3′), 3.59 (bs; 4H, piperazine-H), 2.97 (m; 1H, cyclopentyl-CH), 3.33 (s, 6H, 2CH_3_), 1.71–1.44 ppm (m; 16H, cyclopentyl-CH_2_), ^13^C NMR (DMSO-d_6_): δ_C_ = 161.97 (CO), 161.05 (C=N), 156.08 (C-4,4′), 139.83 (C-6,6′), 130.85 (C-8,8a′), 125.06 (C-5,5′), 121.21 (C-7,7′), 118.62 (C-3,3′), 114.61 (C-8,8′), 104.07 (C-4a, 4a’), 45.31 (piperazine-CH_2_), 30.47 (CH_3_), 28.51, 25.80 ppm (cyclopentyl-CH_2_). *Anal. Calcd for* C_36_H_42_N_6_O_2_: C, 73.19; H, 7.17; N, 14.23; Found: C, 73.38; H, 7.29; N, 14.38.

### 4.2 Biology

#### 4.2.1 Antiproliferative assays

##### 4.2.1.1 Cell viability assay

To study the effect of **4a-l** and **5a-c** on normal cell lines, a cell viability assay was done using the MCF-10A (human mammary gland epithelial) cell line (Abdelbaset et al., 2019; Al-Wahaibi et al., 2020). See Supplementary Appendix S1A.

##### 4.2.1.2 Antiproliferative activity

The newly synthesized compounds **4a-l** ad **5a-c** were investigated for antiproliferative activity against four different types of cancer cells (Marzouk et al., 2020; Mohassab et al., 2021): A-549 (epithelial cancer cell line), MCF-7 (breast cancer cell line), Panc-1 (pancreas cancer cell line), and HT-29 (colon cancer cell line). The IC_50_ of each compound was calculated using doxorubicin as the reference. See Supplementary Appendix S1A.

#### 4.2.2 Topo I and IIα inhibitory activity

Topo I and IIα inhibitory activity of the most active compounds **4b**, **4d**, **4e**, **4i**, and **5c** were determined at 100 μM and 20 µM concentrations using Camptothecin as a positive reference for topo I inhibitory test and Etoposide for topo IIα inhibitory assay (Park et al. 2019). See Supplementary Appendix S1A.

## Data Availability

The original contributions presented in the study are included in the article/Supplementary Material, further inquiries can be directed to the corresponding authors.
