# Supplementary material for: Synthesis of novel amidines via one-pot three component reactions: Selective topoisomerase I inhibitors with antiproliferative properties
Source: Front Chem. 2022 Nov 18;10:1039176. doi: 10.3389/fchem.2022.1039176 (PMC9716094; doi:10.3389/fchem.2022.1039176)
Supplement: Supplementary file 1 [file DataSheet1.docx]

**Synthesis of novel amidines *via* one-pot three component reactions: Selective topoisomerase I inhibitors with antiproliferative properties**

Essmat M. El-Sheref*^1^, Hendawy N. Tawfeek^1^, Alaa A. Hassan^1^, S. Bräse*^2^, Mohammed A. I. Elbastawesy^3^, Hesham A. M. Gomaa^4^, Bahaa G. M. Youssif*^5^

^1^Chemistry Department, Faculty of Science, Minia University, El Minia, 61519 Egypt; ^2^Institute of Biological and Chemical Systems, IBCS-FMS, Karlsruhe Institute of Technology, 76131 Karlsruhe, Germany; ^3^Department of Pharmaceutical Organic Chemistry, Faculty of Pharmacy, Al-Azhar University, 71524 Assiut, Egypt; ^4^Pharmacology Department, College of Pharmacy, Jouf University, Sakaka 72314, Saudi Arabia; ^5^Pharmaceutical Organic Chemistry Department, Faculty of Pharmacy, Assiut University, Assiut 71526, Egypt.

**Spectral data for compound 4a**

**
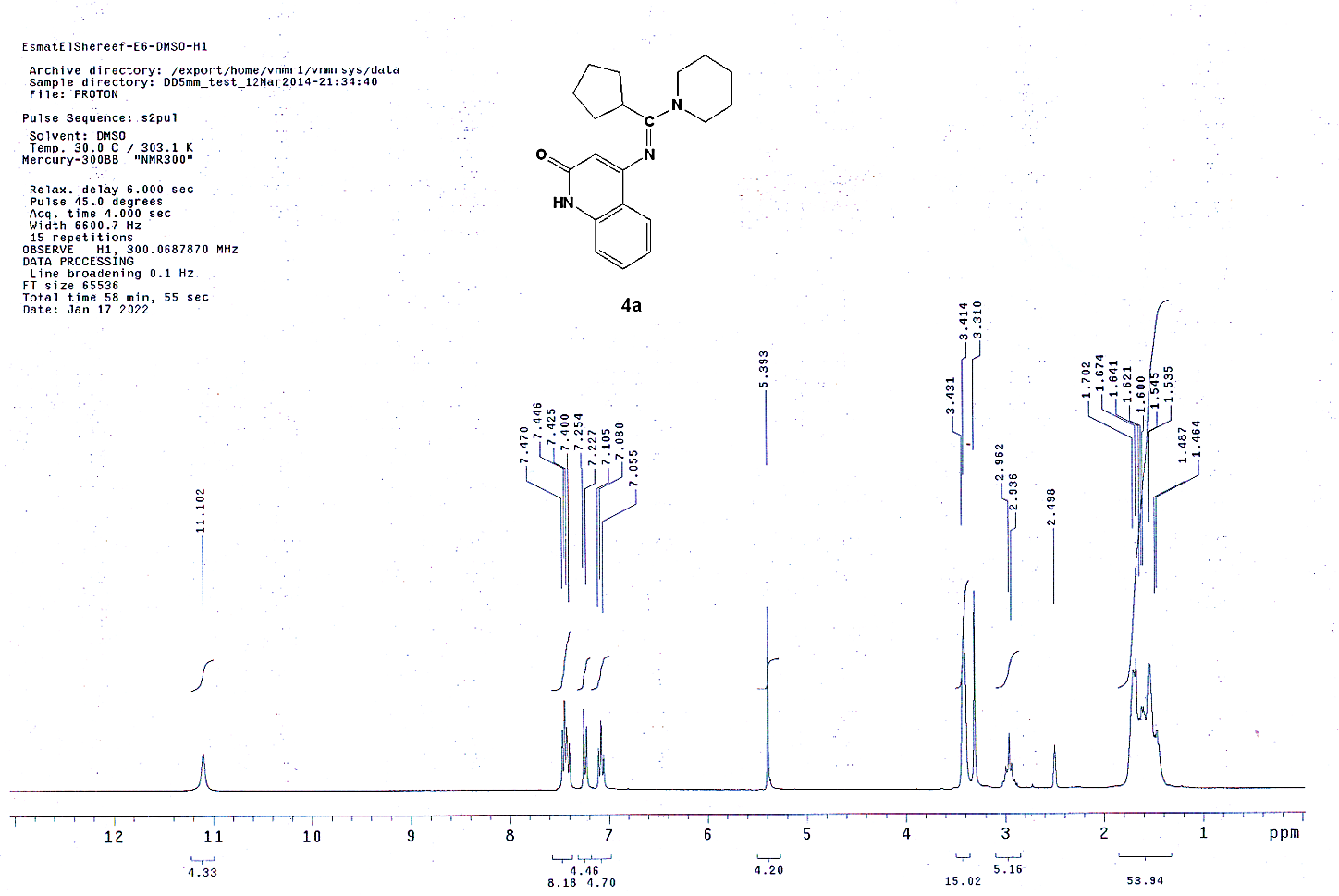
**

**Figure 1.** ^1^H-NMR spectrum for compound **4a.**

**
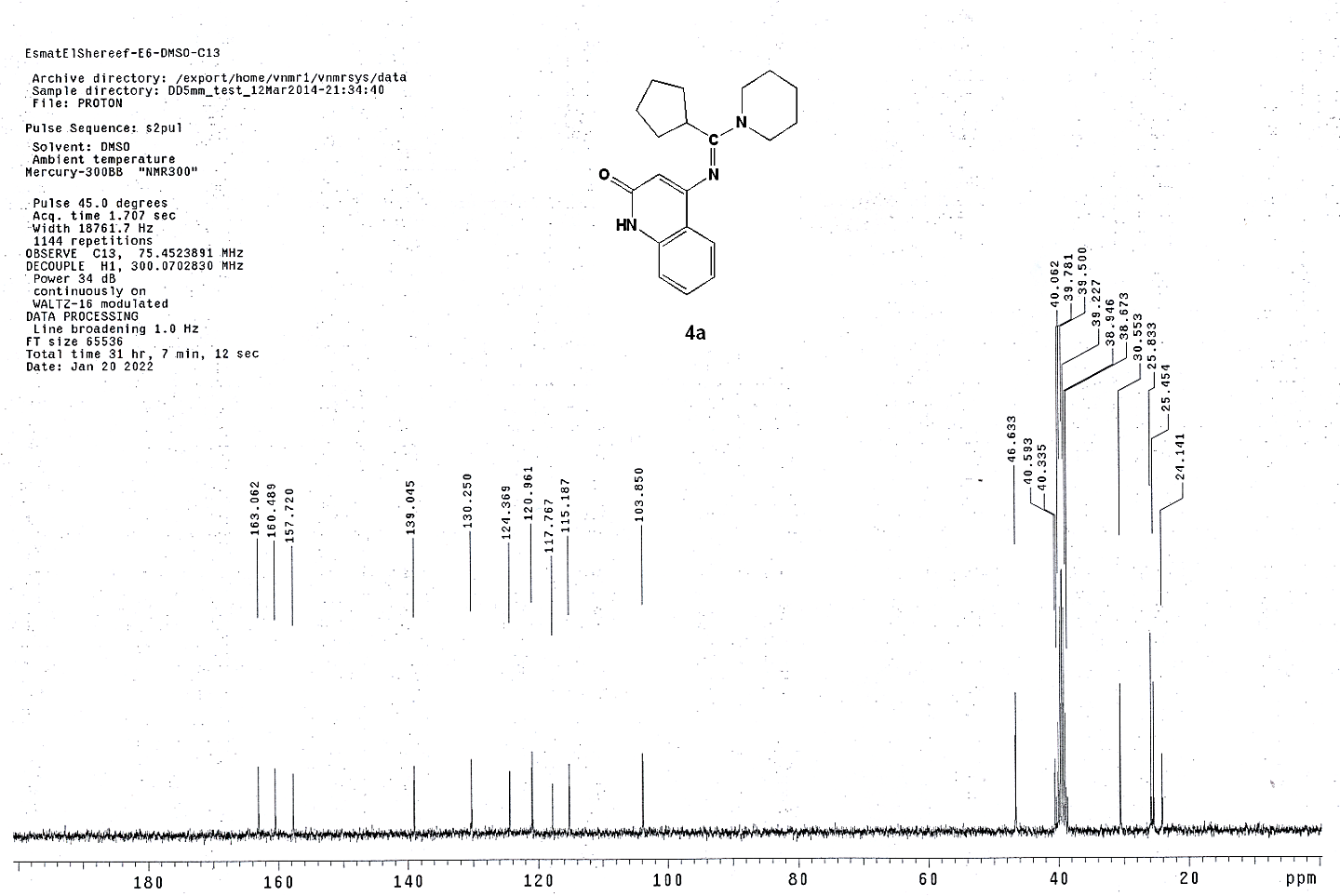
**

**Figure 2.** ^13^C-NMR spectrum for compound **4a.**

**Spectral data for compound 4b.**

**
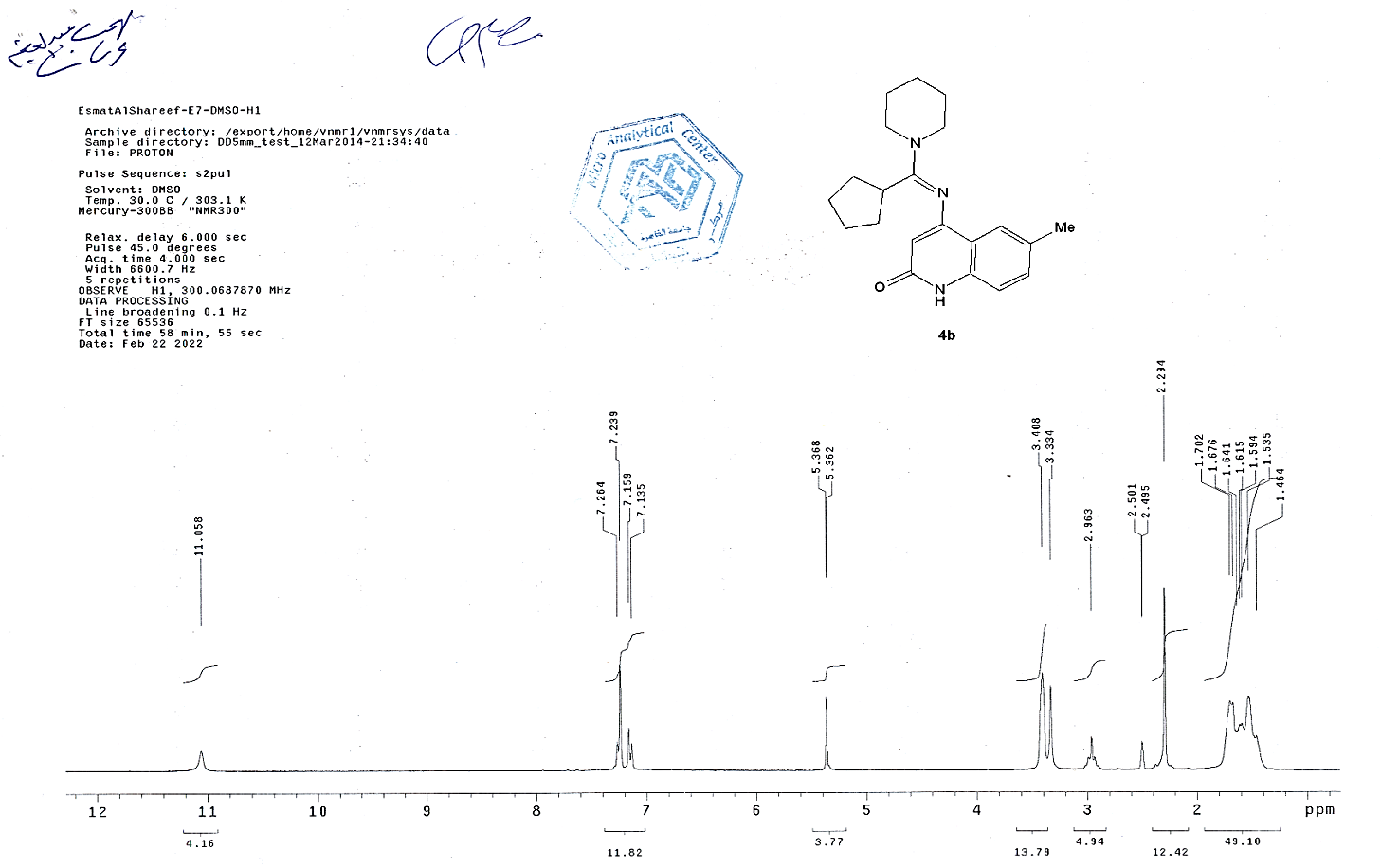
**

**Figure 3.** ^1^H-NMR spectrum for compound **4b.**

**
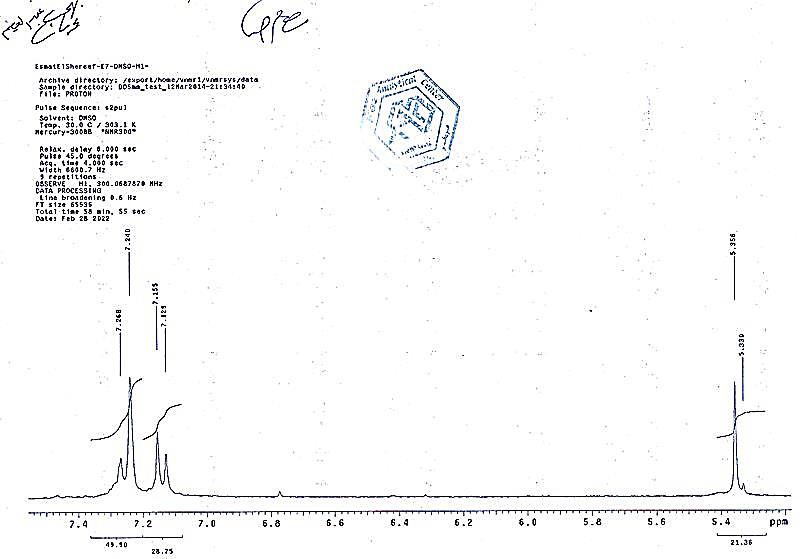
**

**Figure 4.** Part of the ^1^H-NMR spectrum for compound **4b.**

**
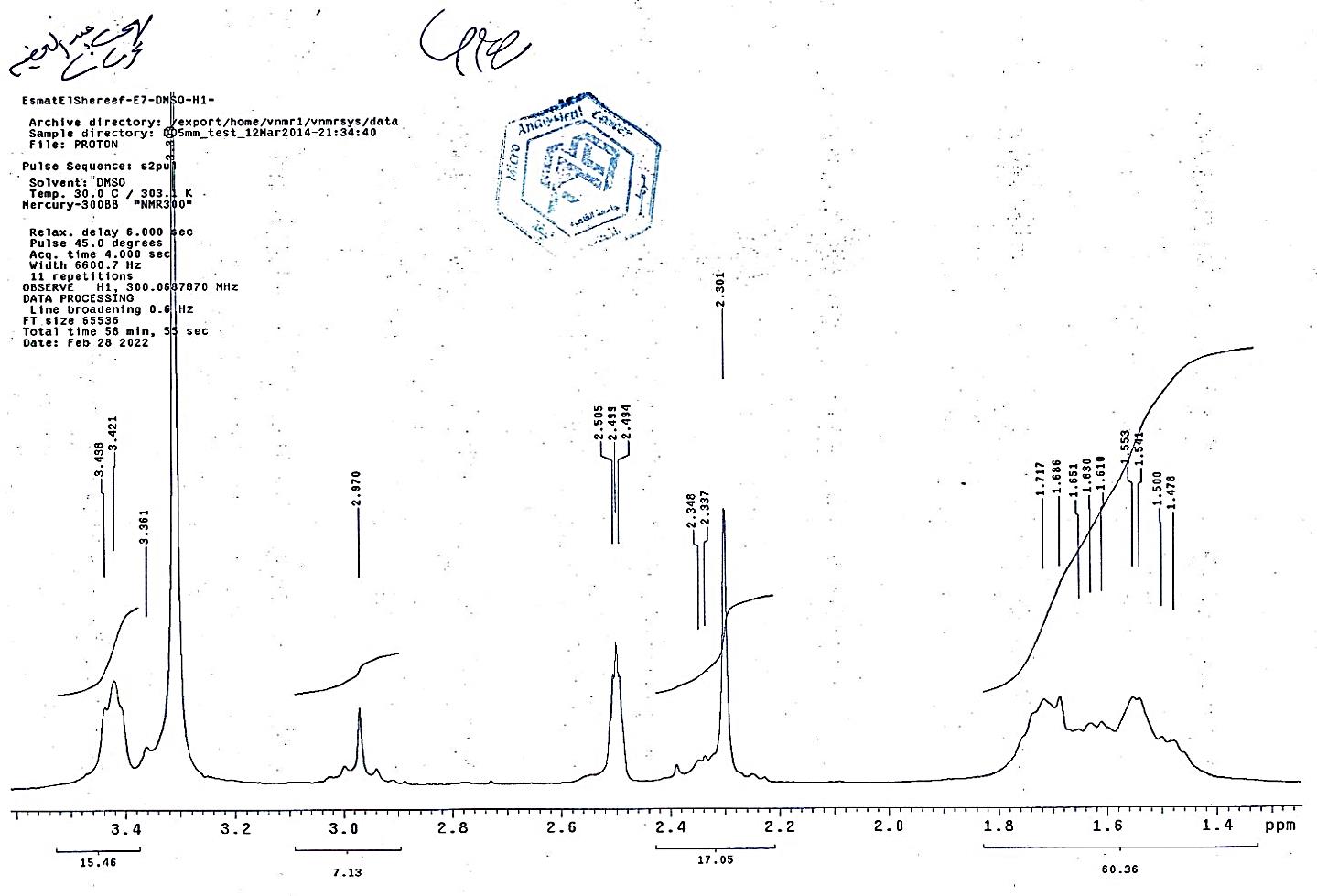
**

**Figure 5.** Part of the ^1^H-NMR spectrum for compound **4b.**

**
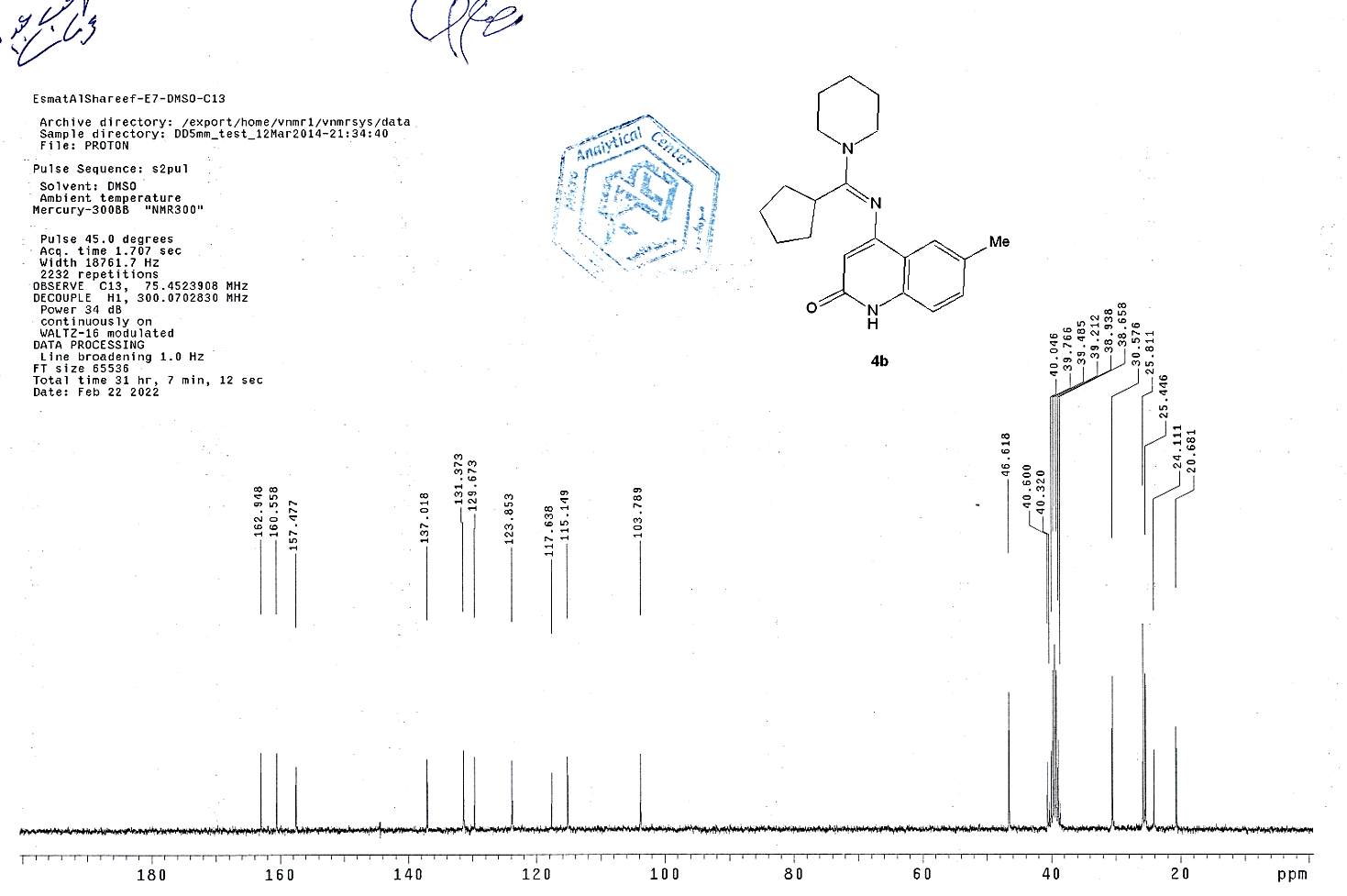
**

**Figure 6.** ^13^C-NMR spectrum for compound **4b.**


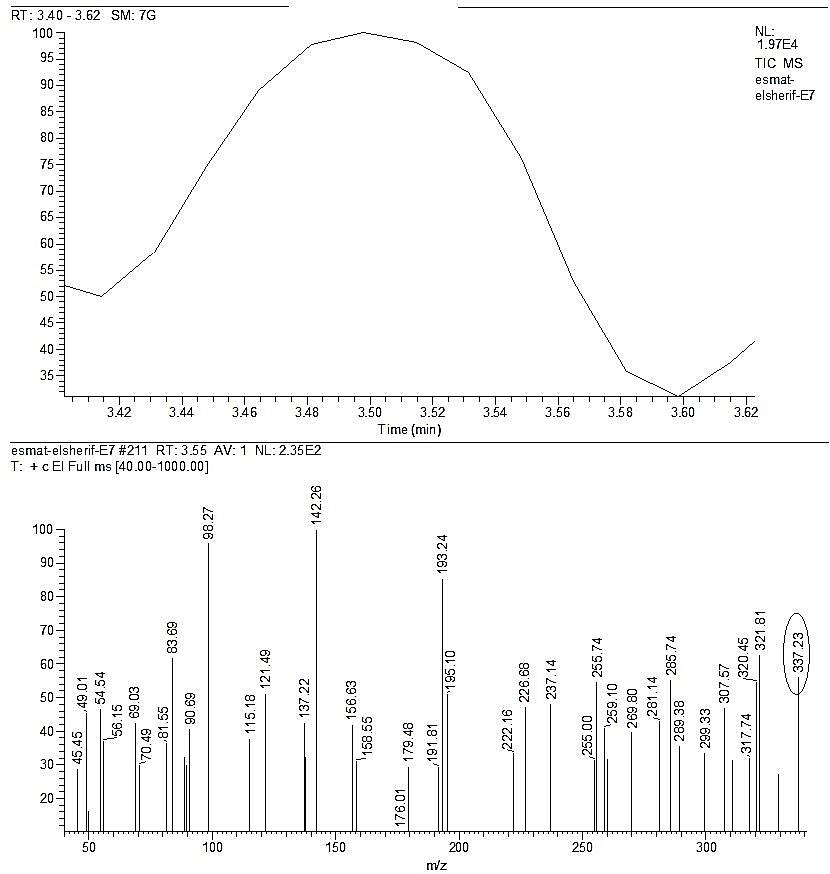


**Figure 7.** Mass spectrometry for compound **4b.**

**Spectral data for compound 4c.**

**
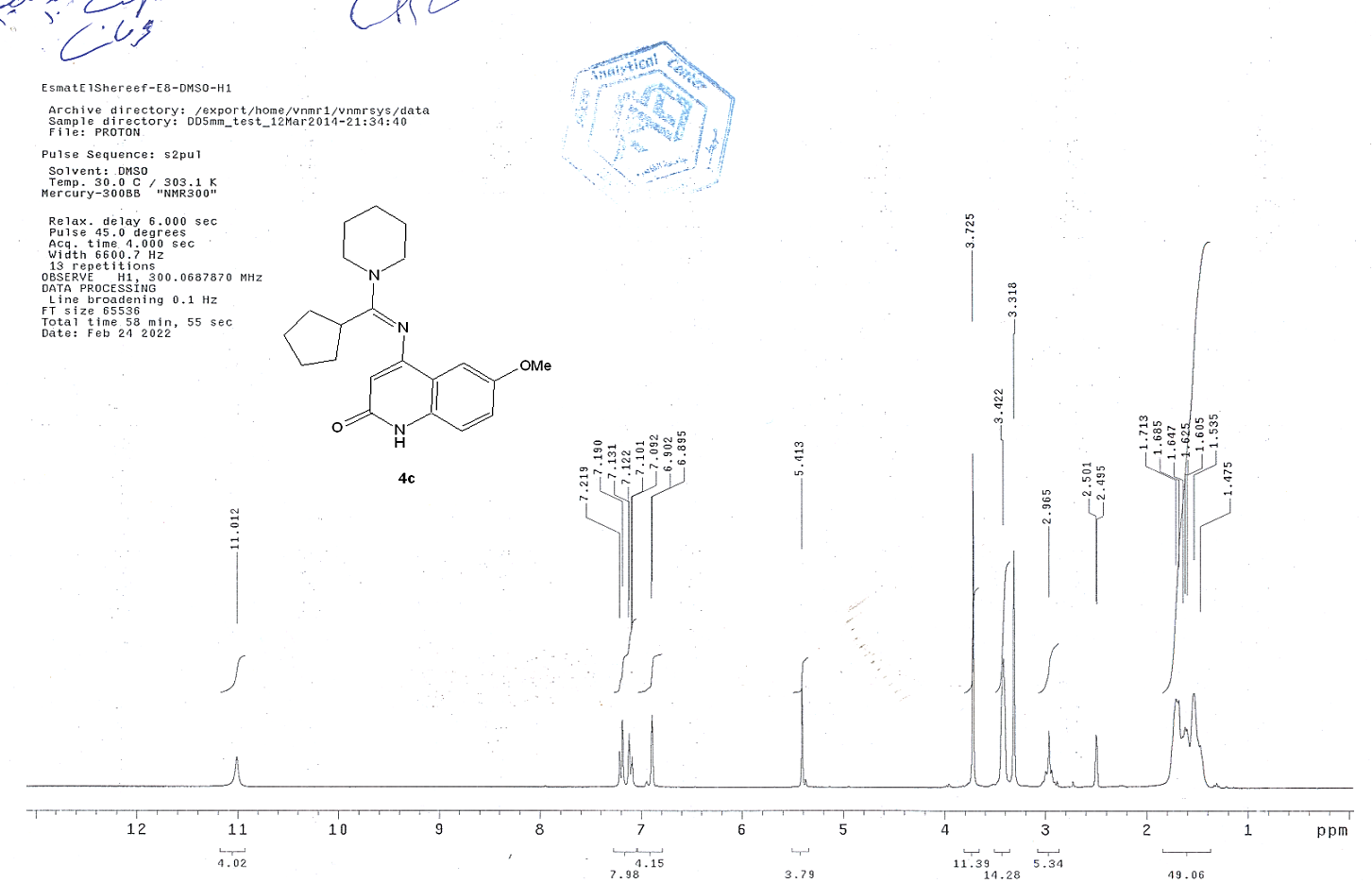
**

**Figure 8.** ^1^H-NMR spectrum for compound **4c.**


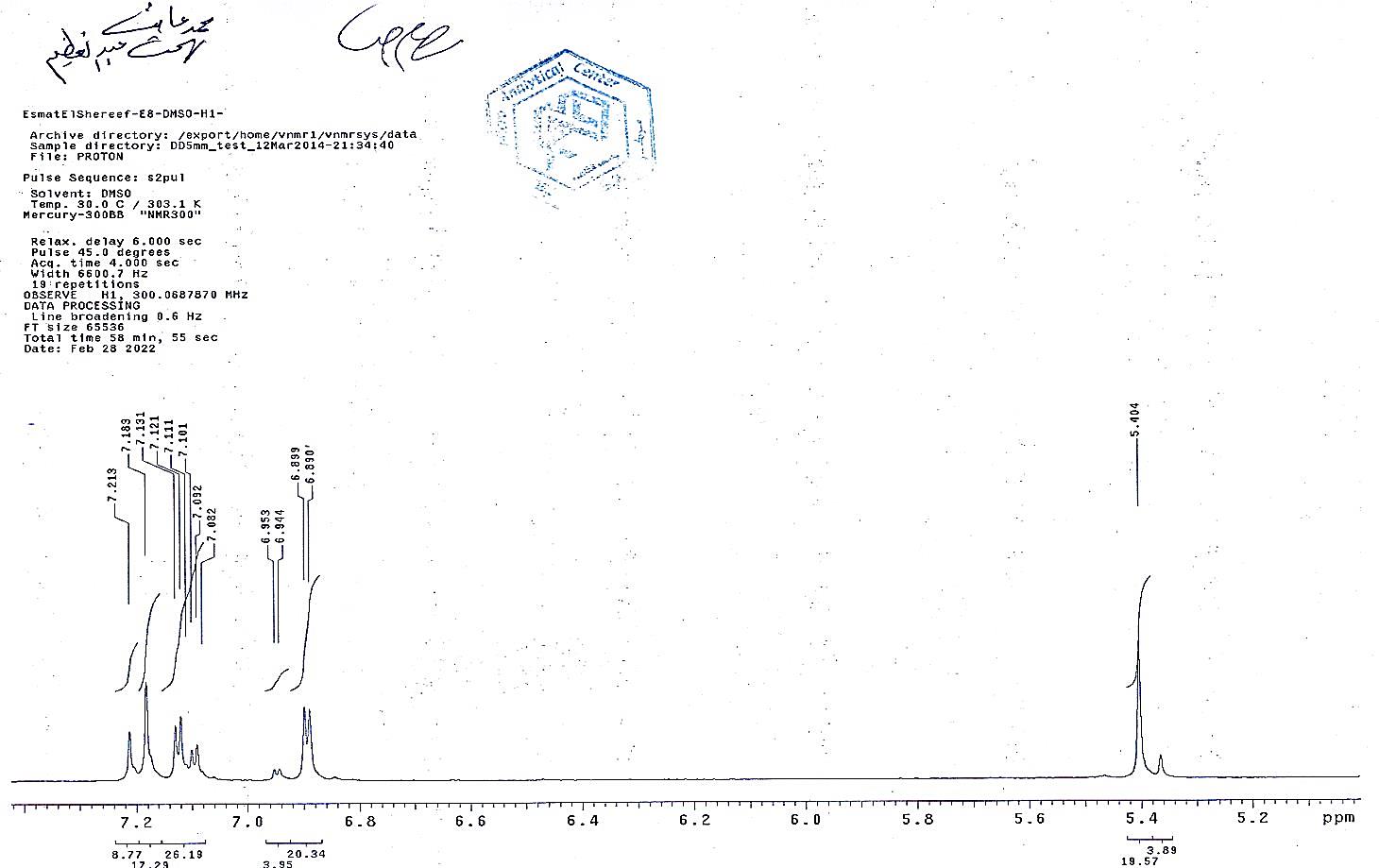


**Figure 9.** Part of the ^1^H-NMR spectrum for compound **4c.**


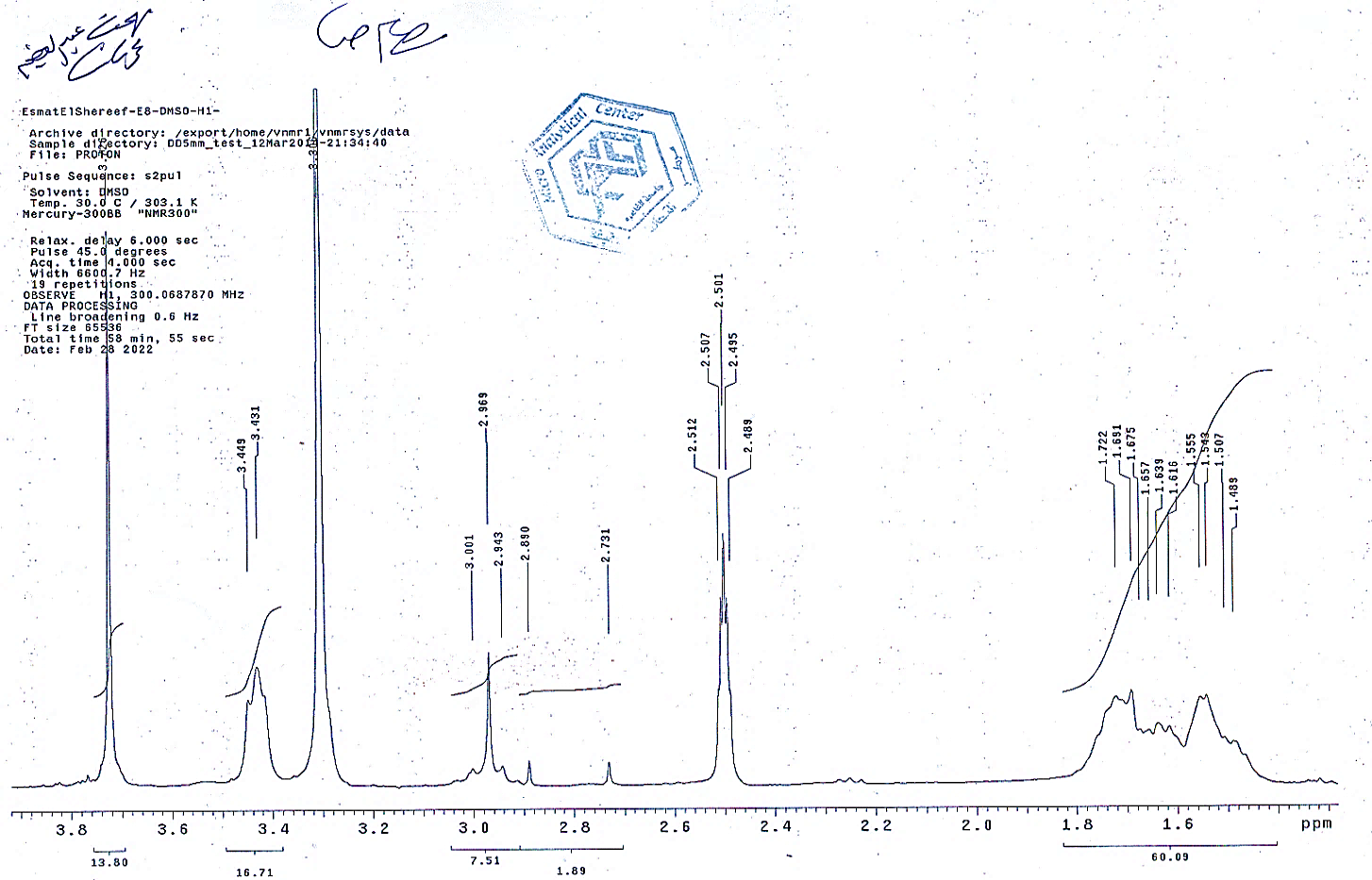


**Figure 10.** Part of the ^1^H-NMR spectrum for compound **4c.**


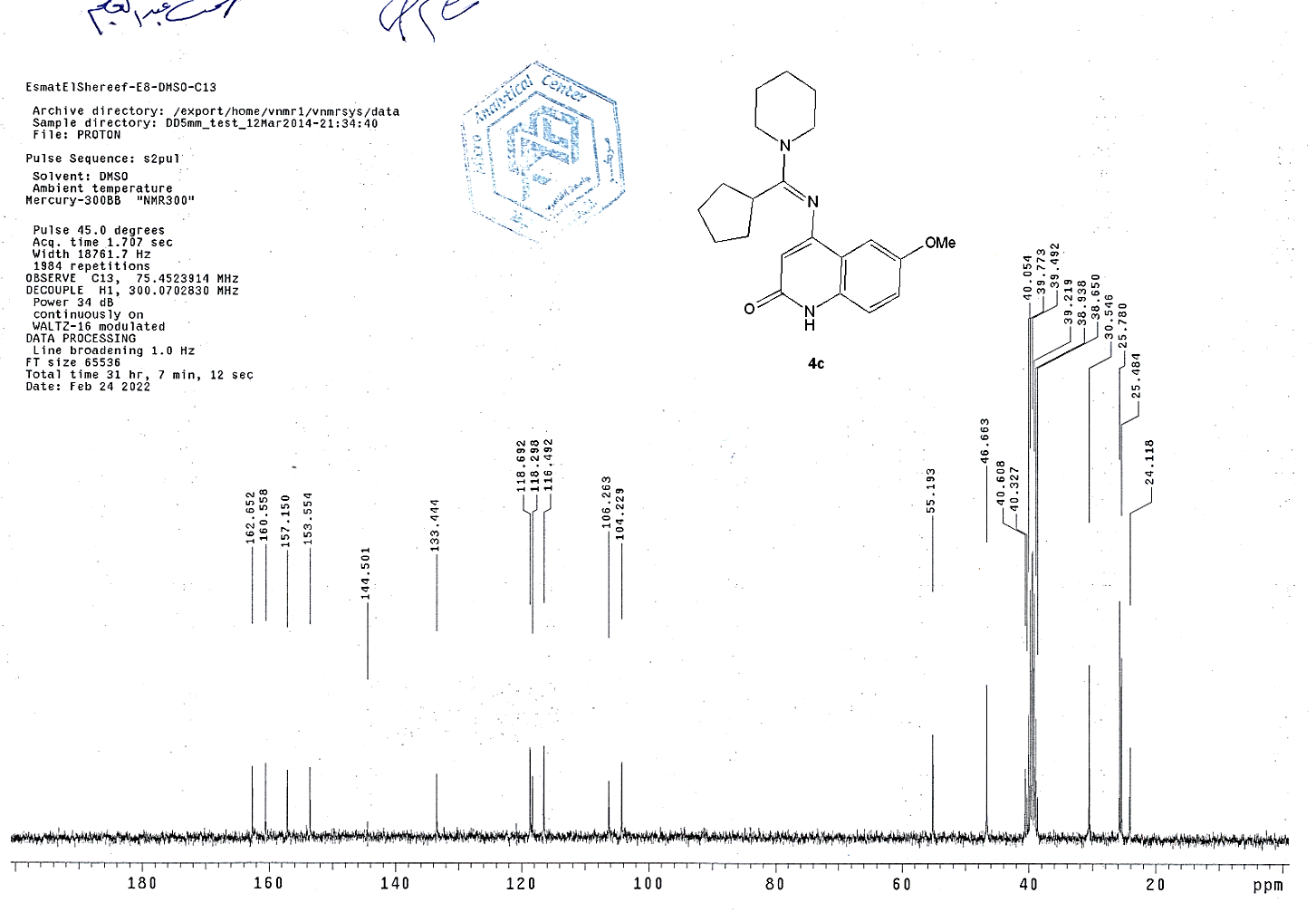


**Figure 11.** ^13^C-NMR spectrum for compound **4c.**

**Spectral data for compound 4d.**

**
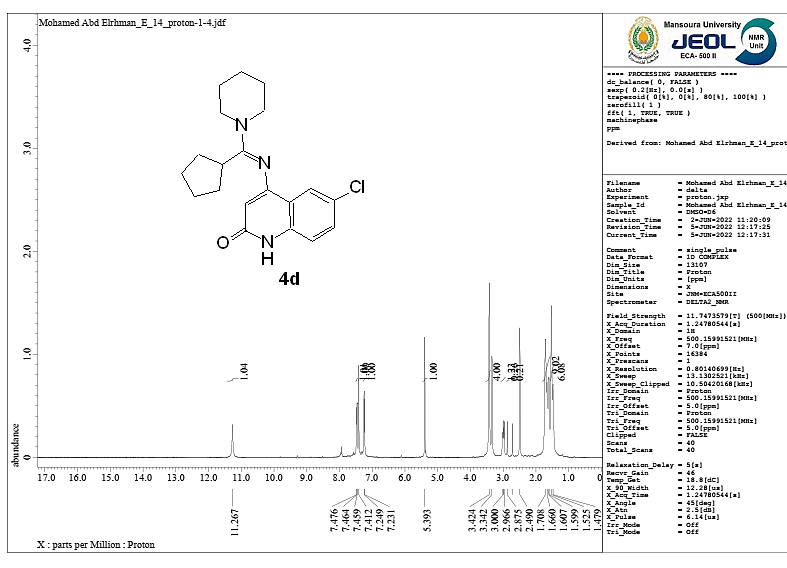
**

**Figure 12.** ^1^H-NMR spectrum for compound **4d.**


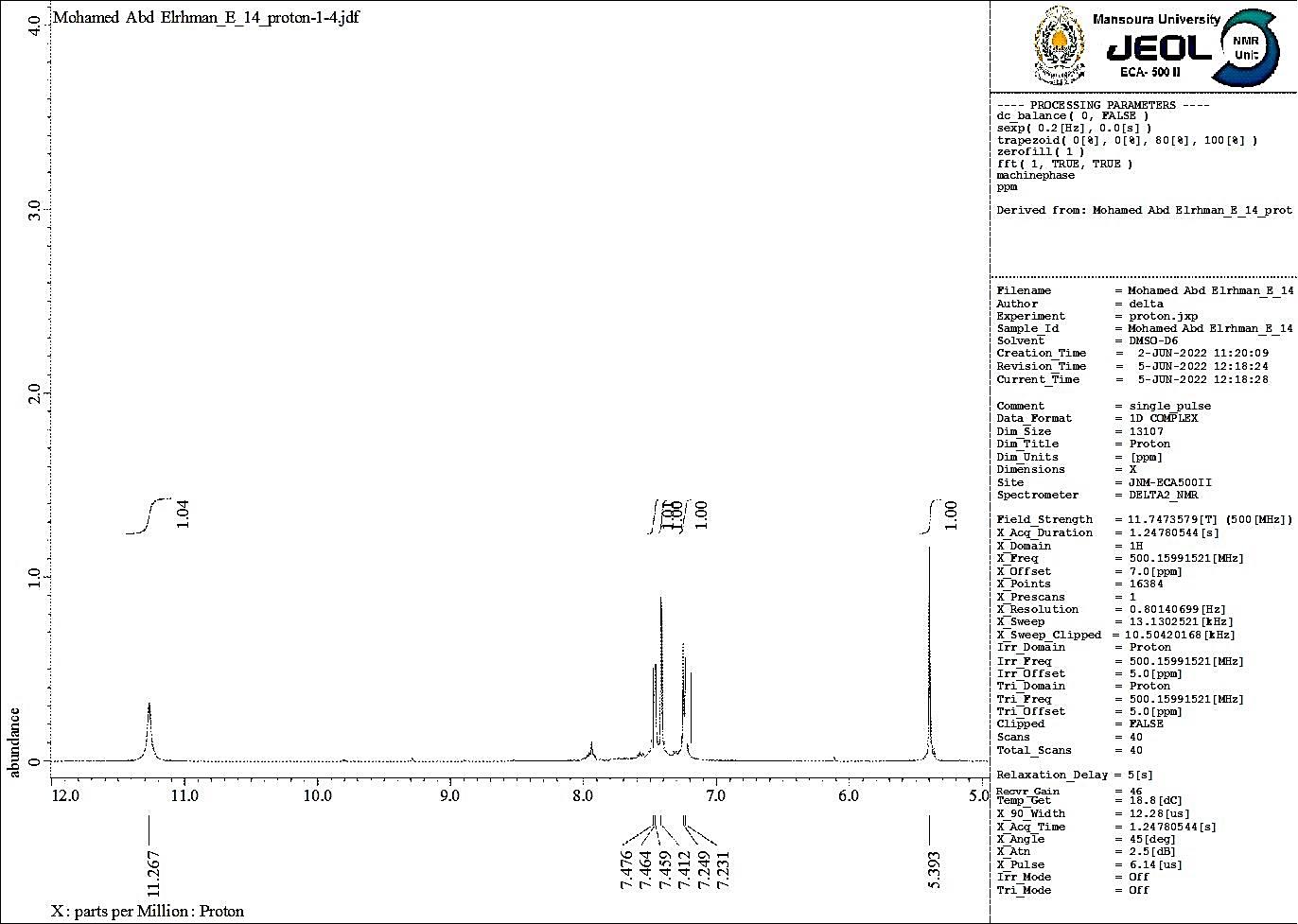


**Figure 13.** Part of the ^1^H-NMR spectrum for compound **4d.**


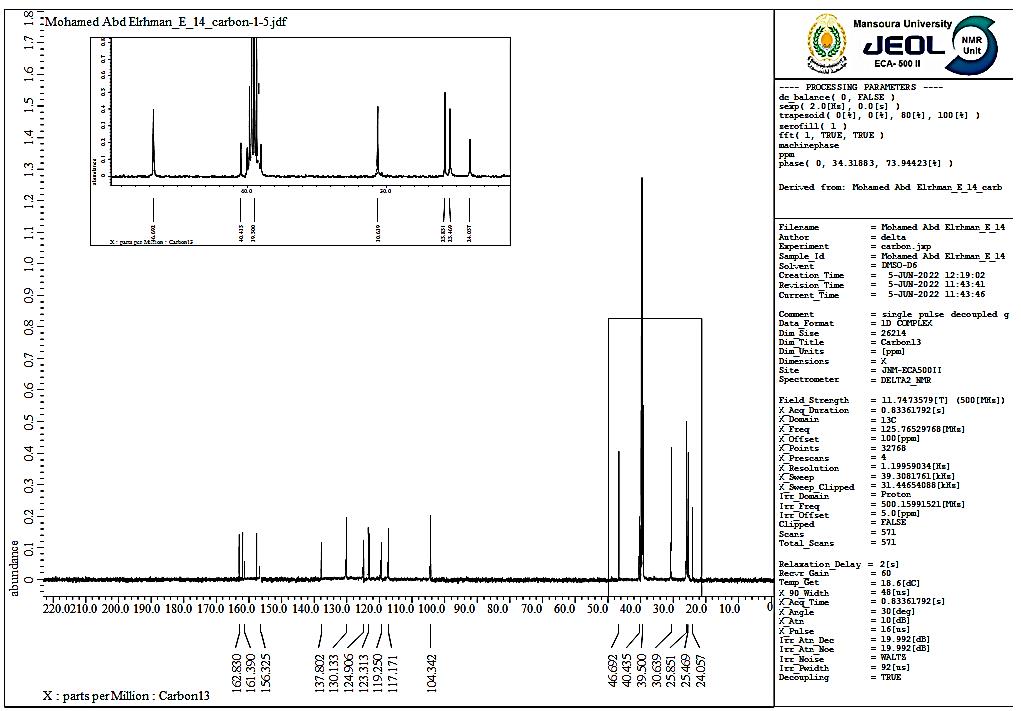


**Figure 14.** ^13^C-NMR spectrum for compound **4d.**

**Spectral data for compound 4e.**

**
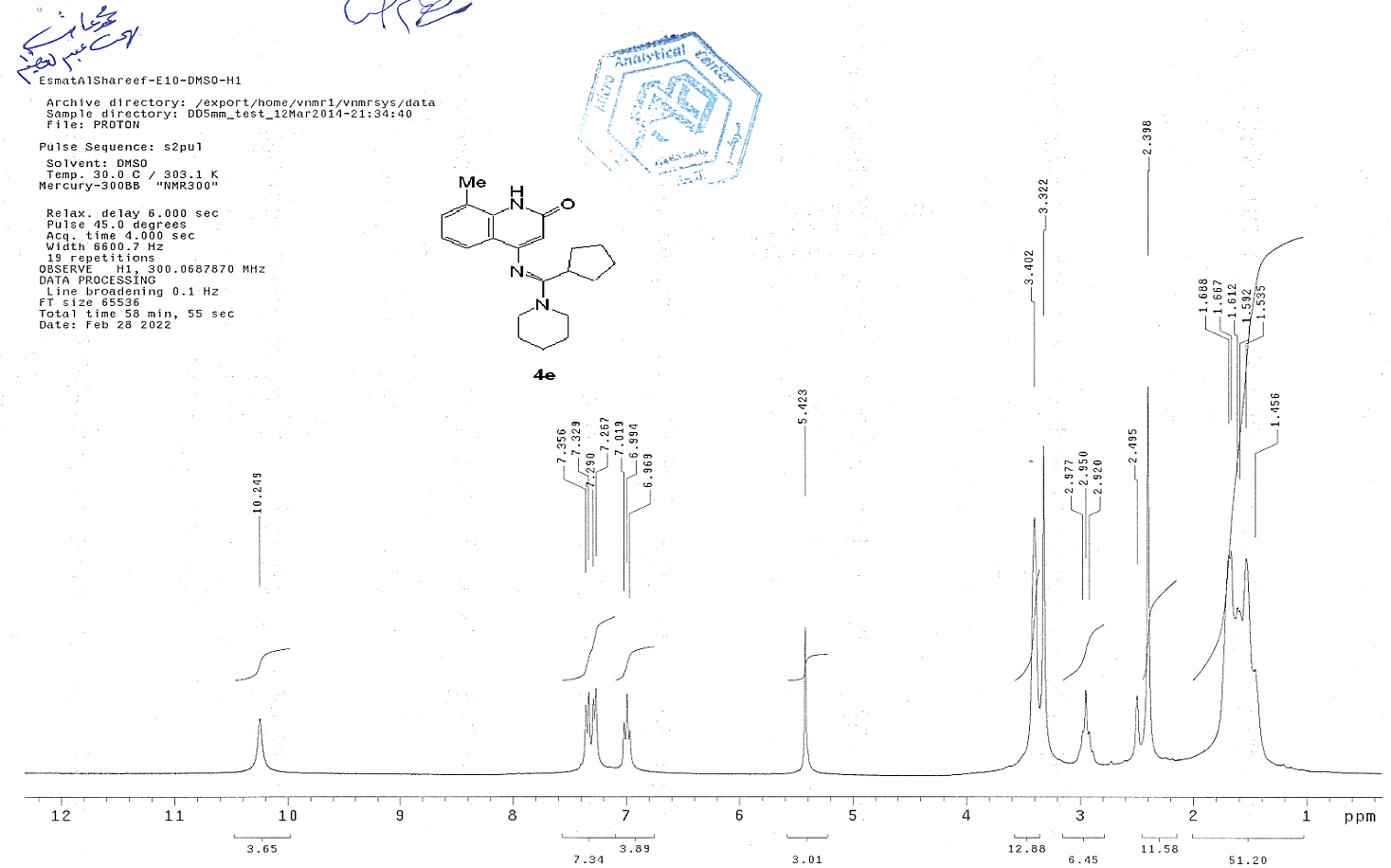
**

**Figure 15.** ^1^H-NMR spectrum for compound **4e.**


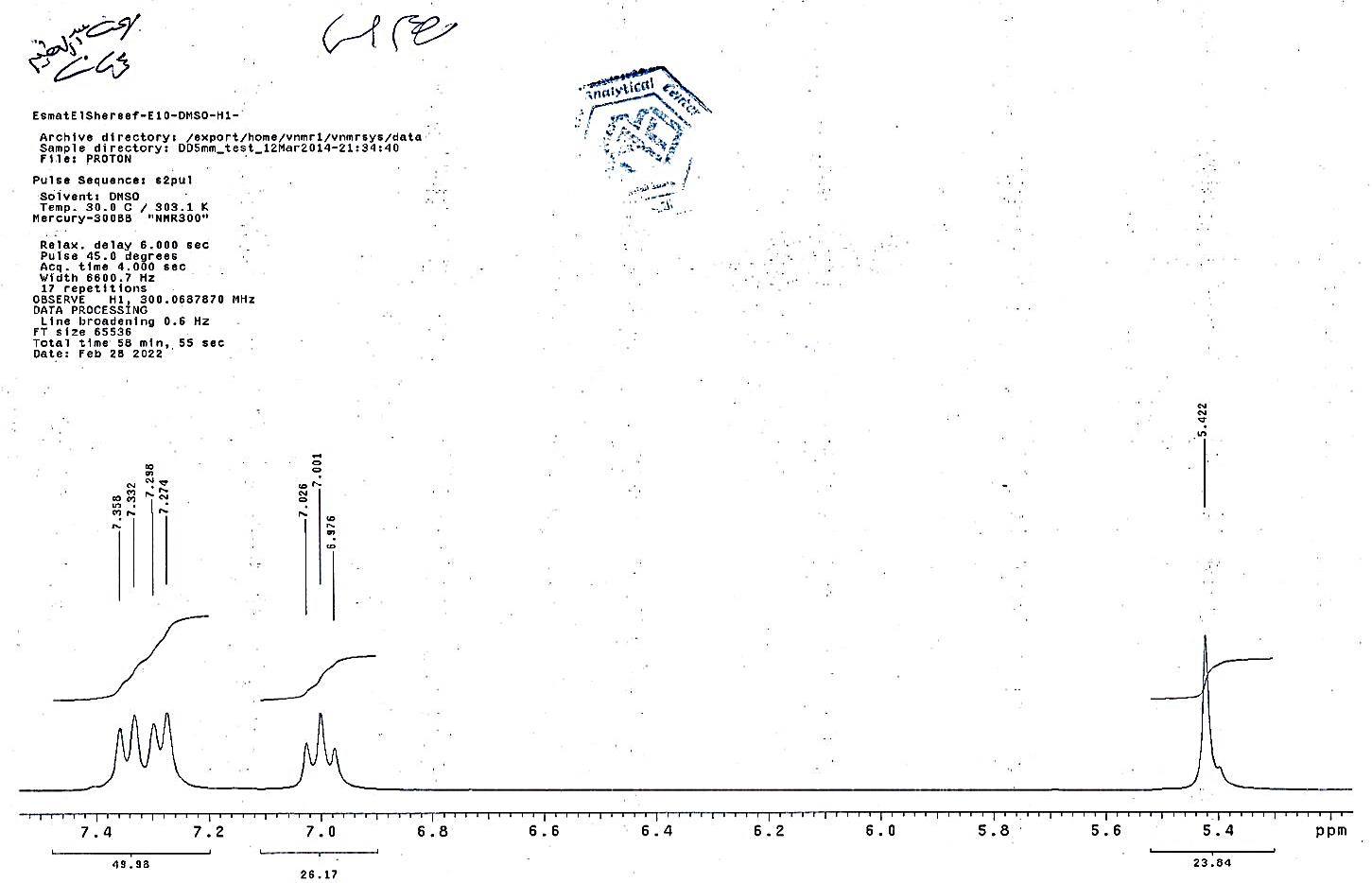


**Figure 16.** Part of the ^1^H-NMR spectrum for compound **4e.**


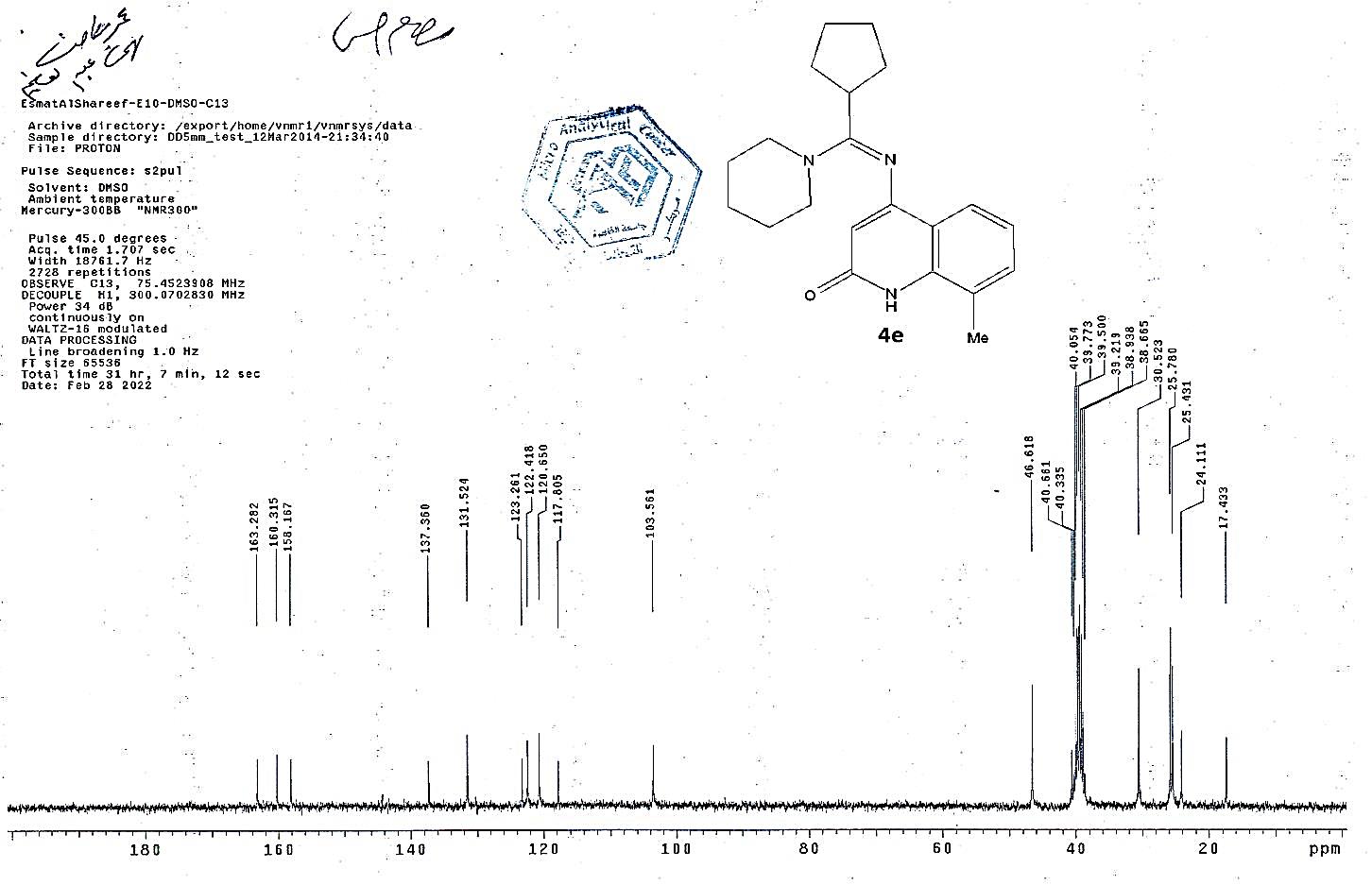


**Figure 17.** ^13^C-NMR spectrum for compound **4e.**

**Spectral data for compound 4f.**

**
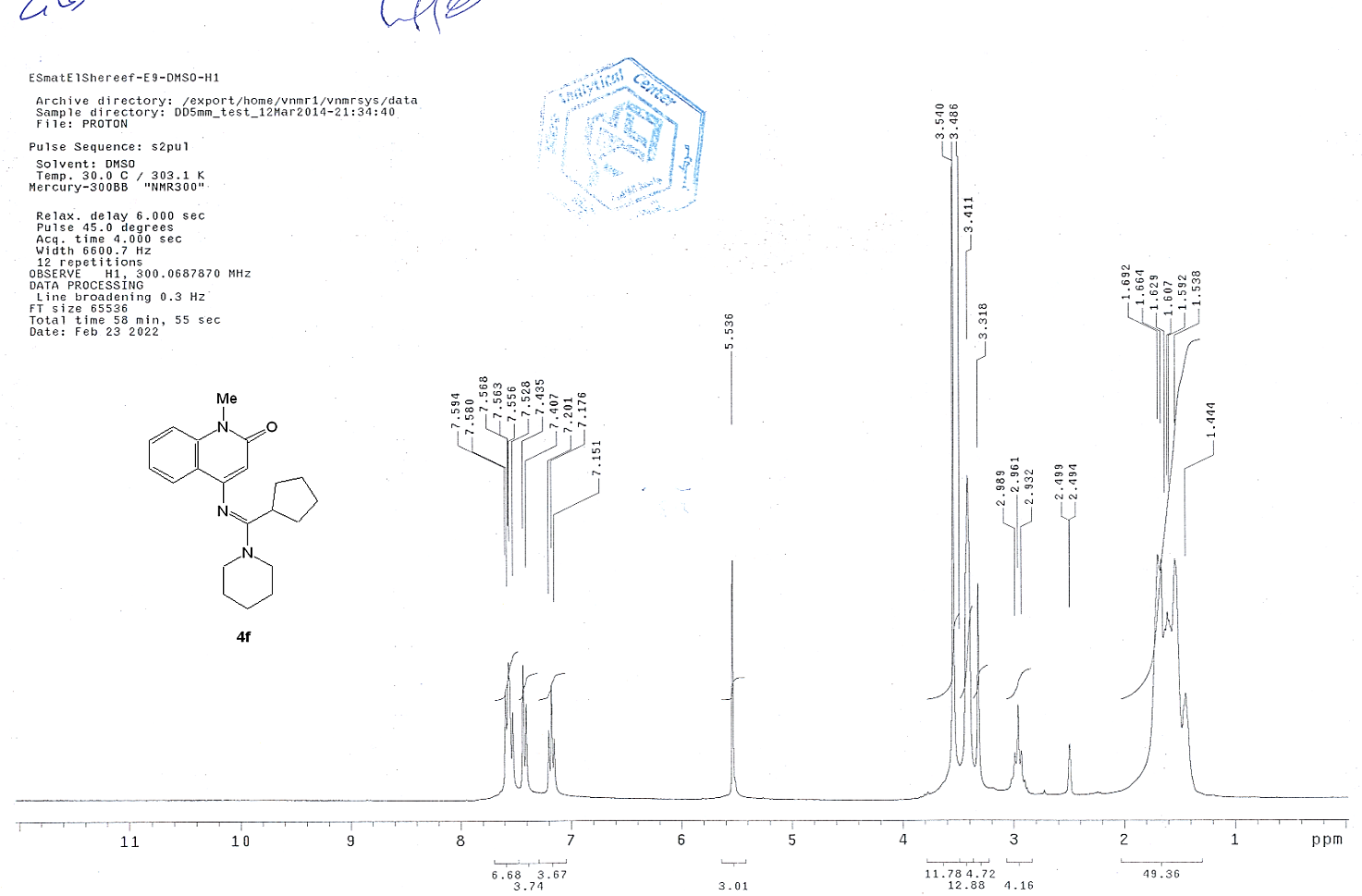
**

**Figure 18.** ^1^H-NMR spectrum for compound **4f.**


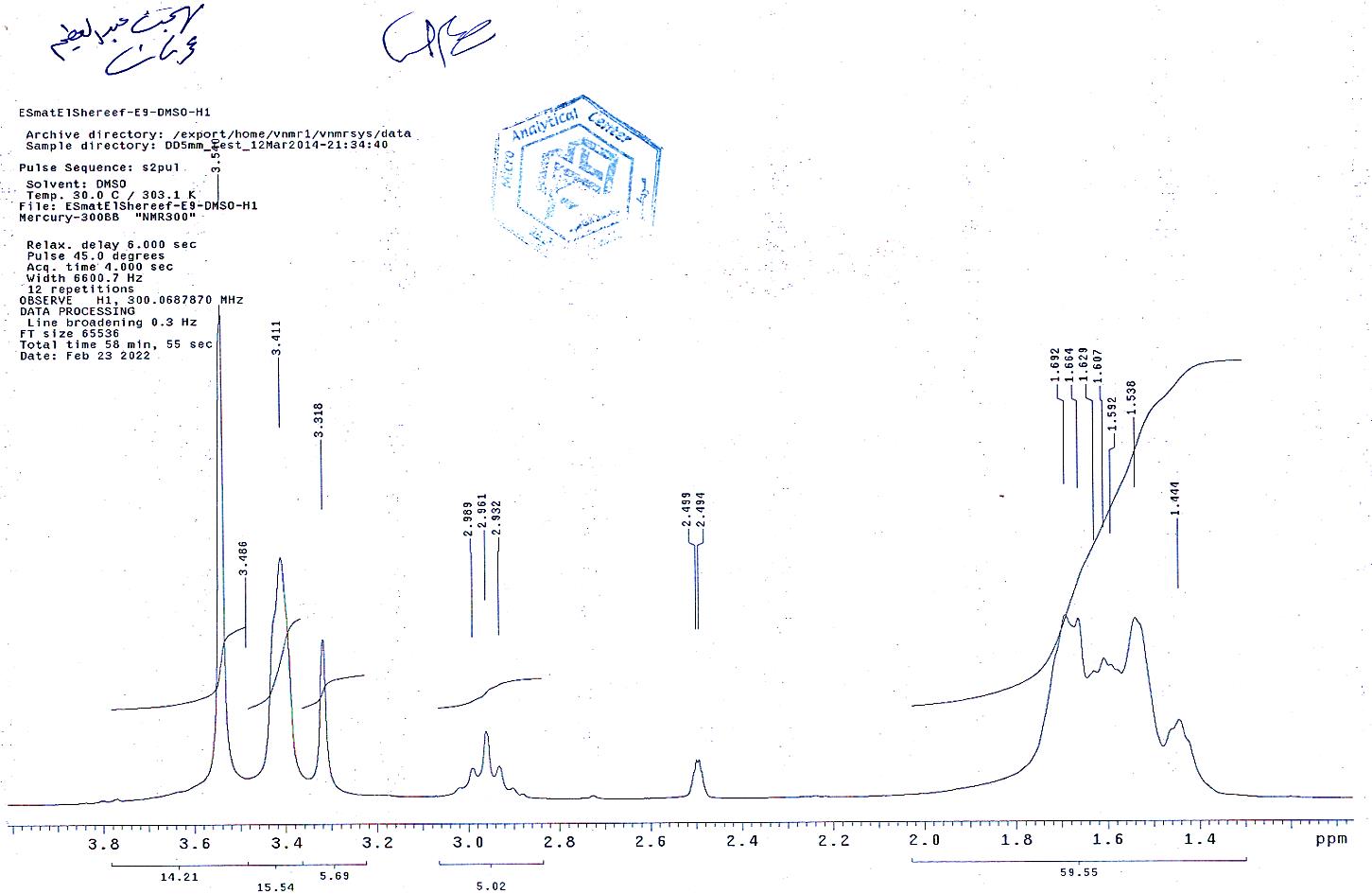


**Figure 19.** Part of the ^1^H-NMR spectrum for compound **4f.**


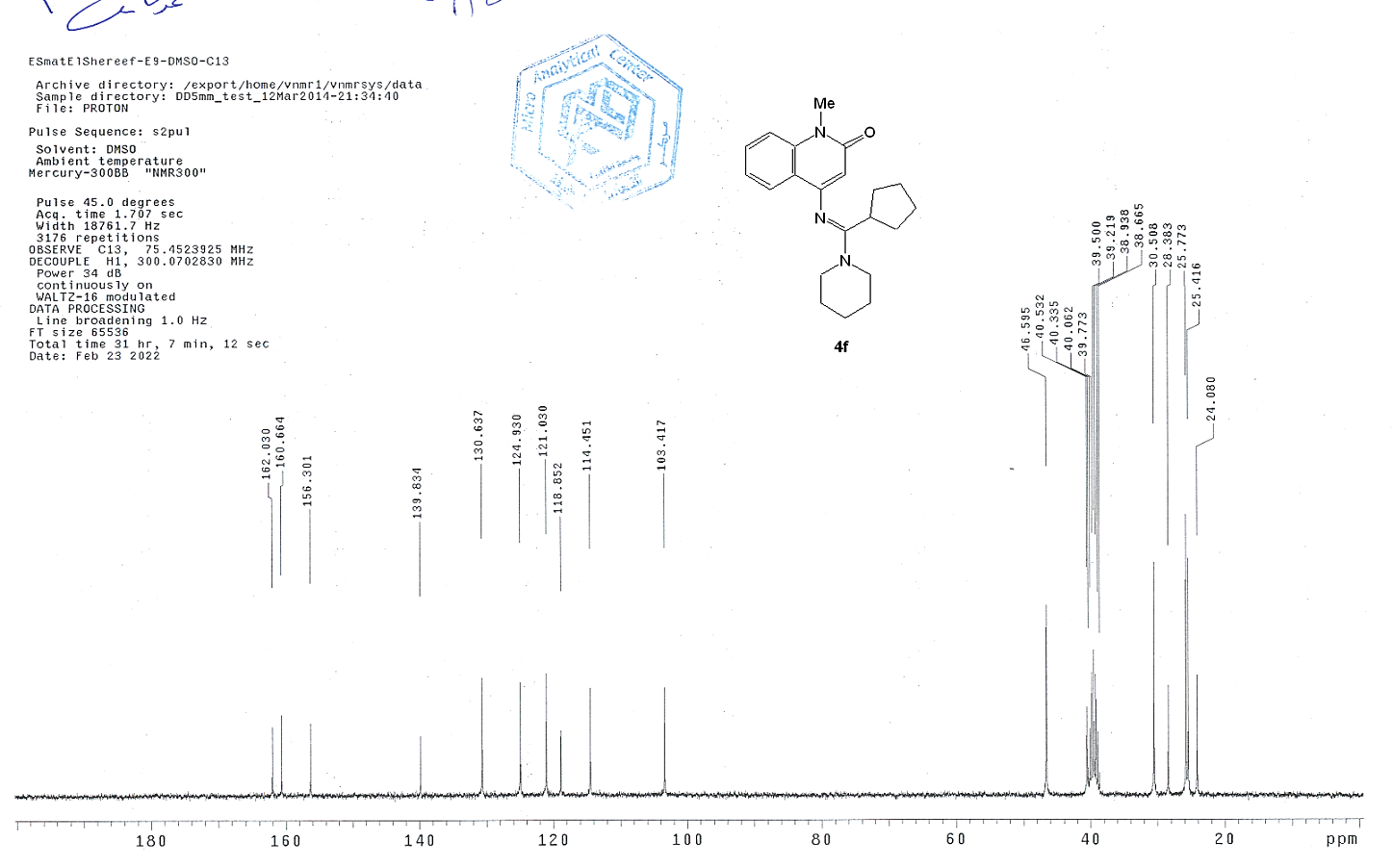


**Figure 20.** ^13^C-NMR spectrum for compound **4f.**

**Spectral data for compound 4g.**

**
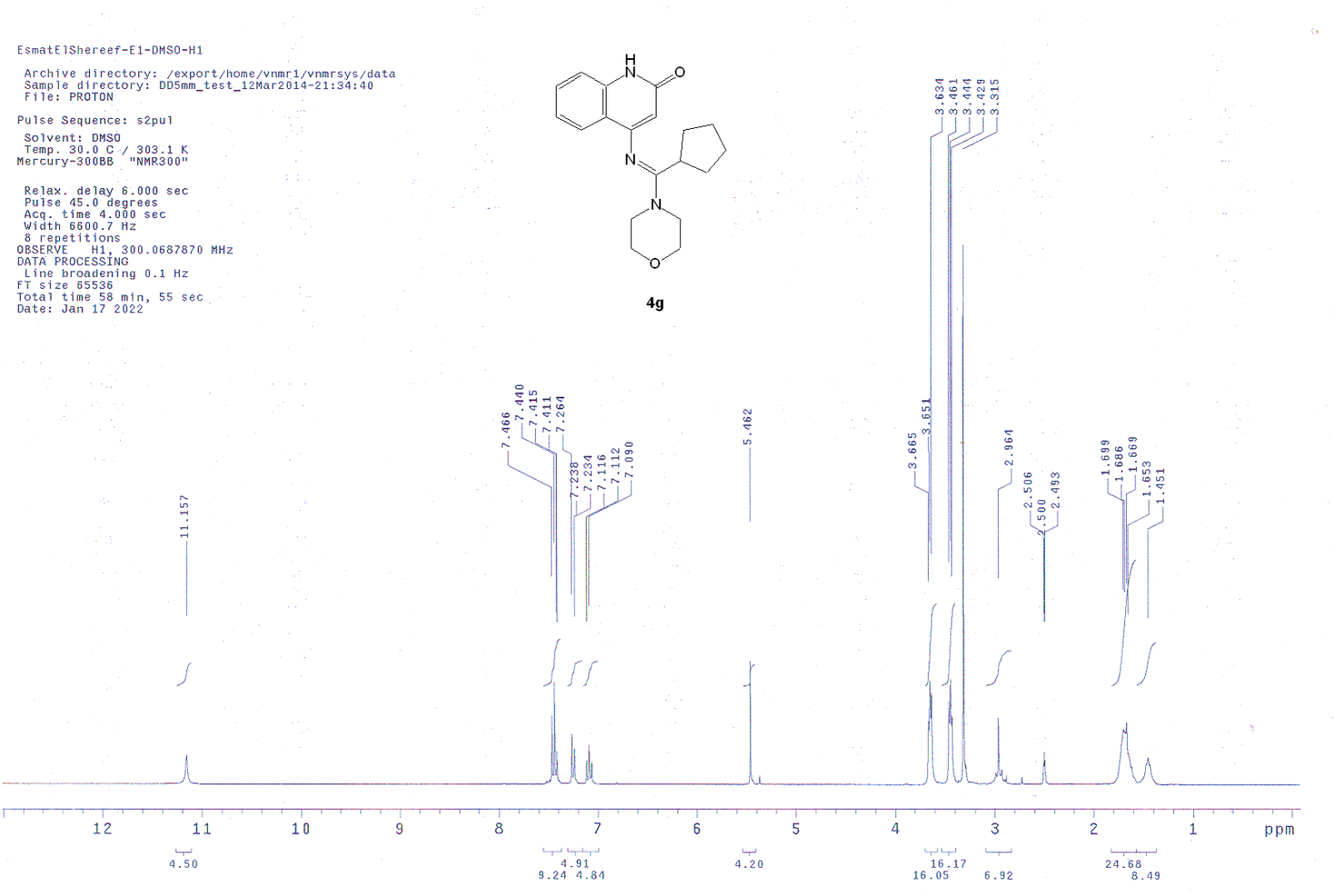
**

**Figure 21.** ^1^H-NMR spectrum for compound **4g.**


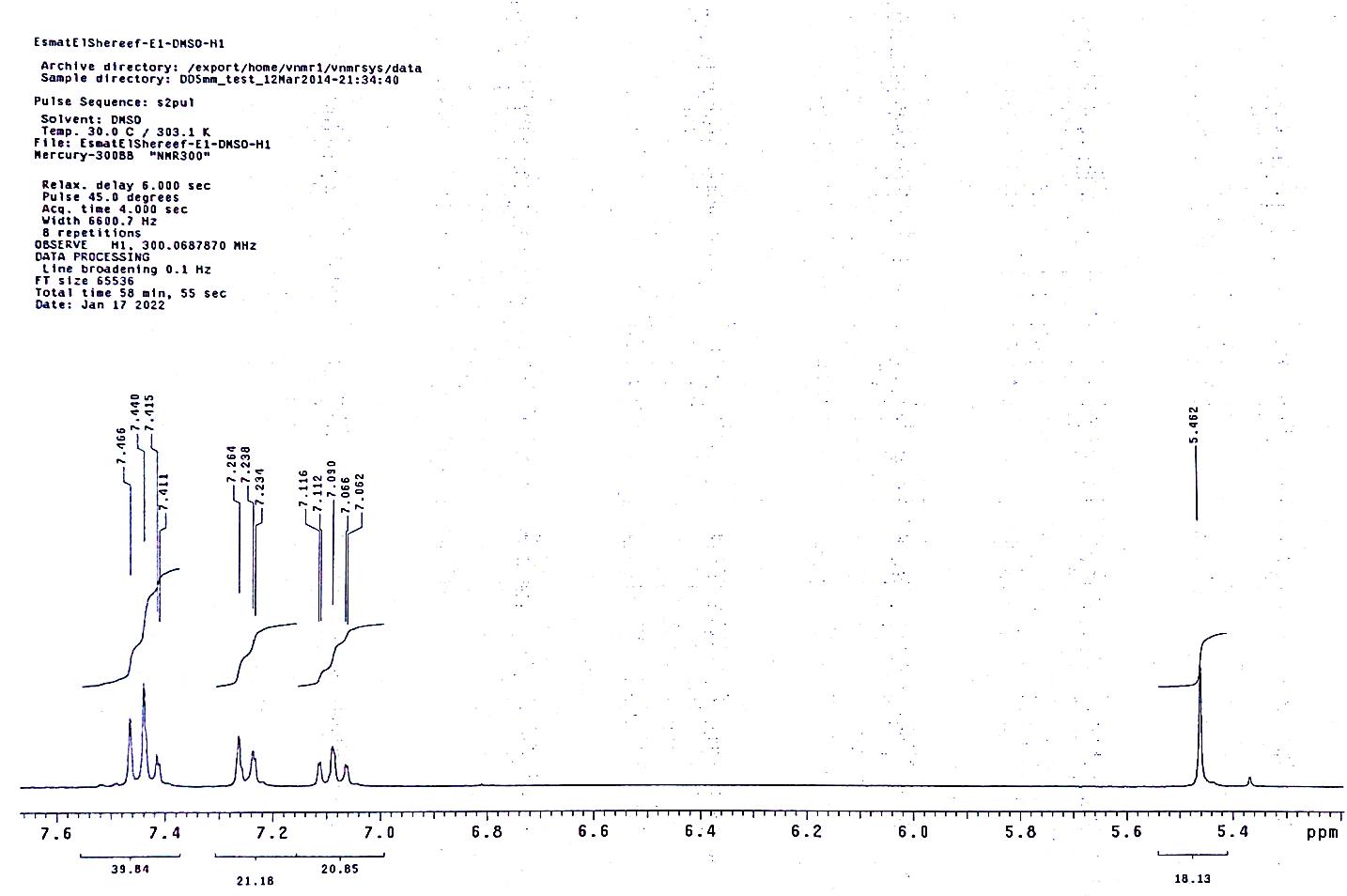


**Figure 22.** Part of the ^1^H-NMR spectrums for compound **4g.**

**
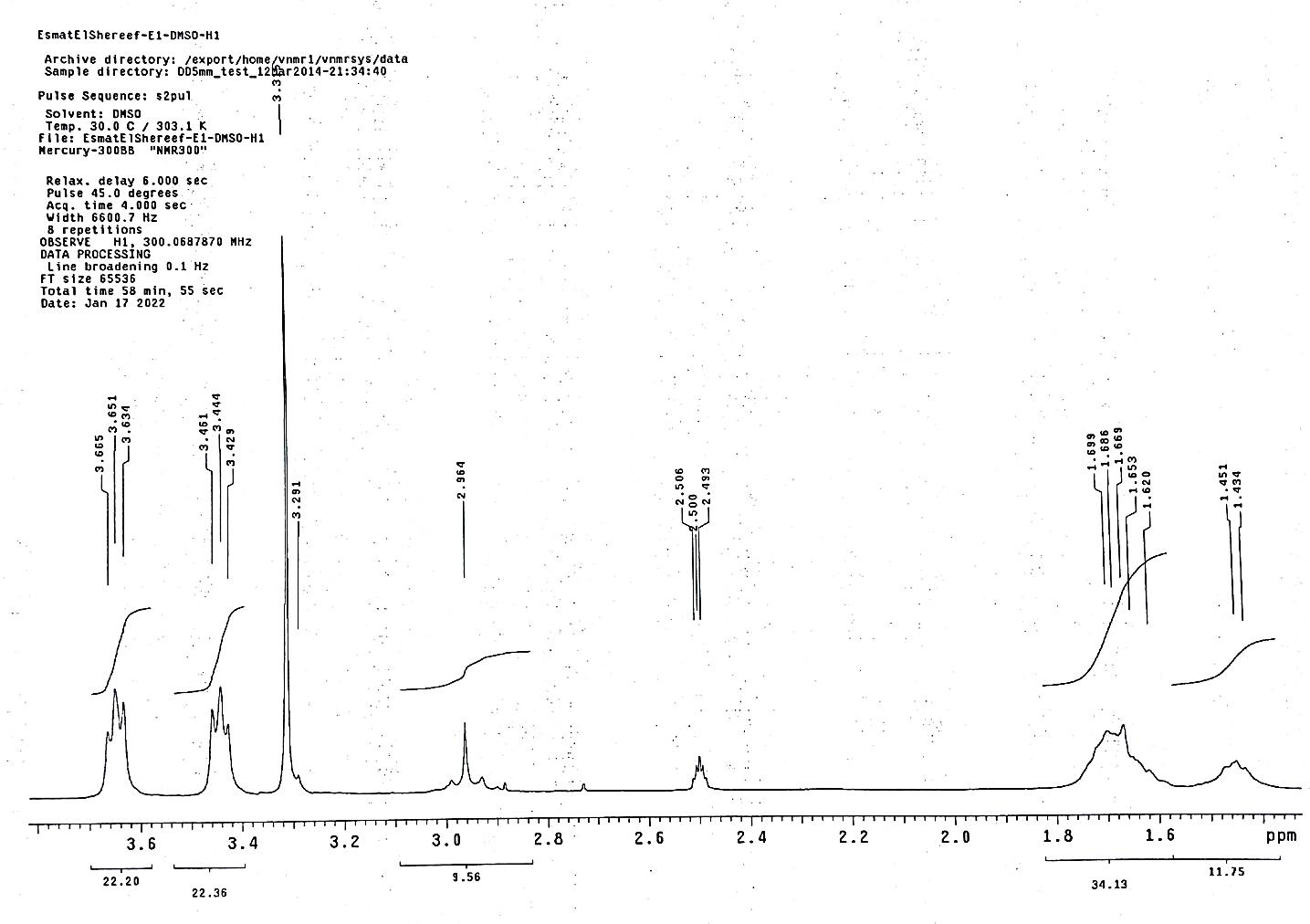
**

**Figure 23.** Part of the ^1^H-NMR spectrum for compound **4g.**

**
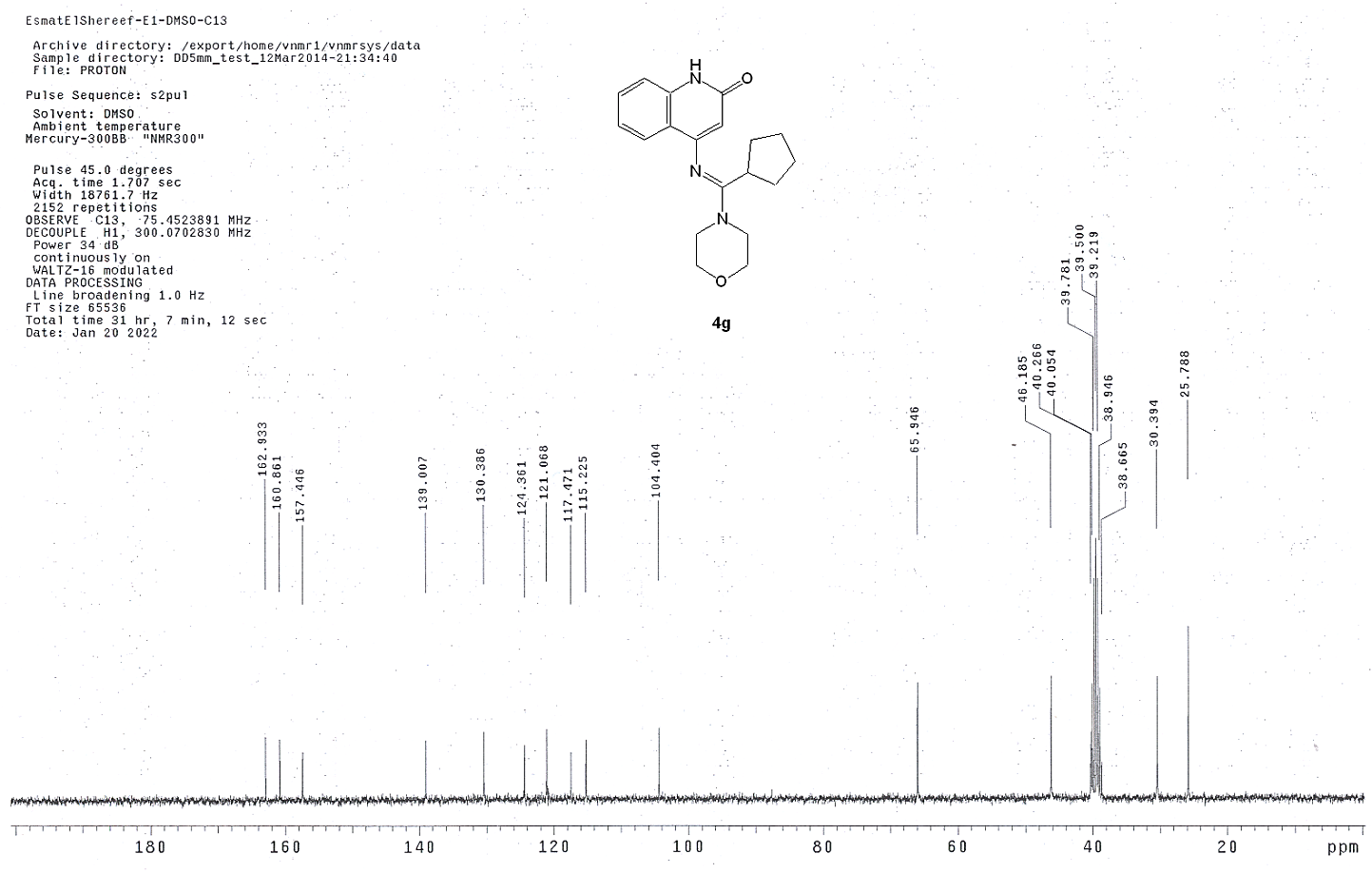
**

**Figure 24.** ^13^C-NMR spectrum for compound **4g.**

**
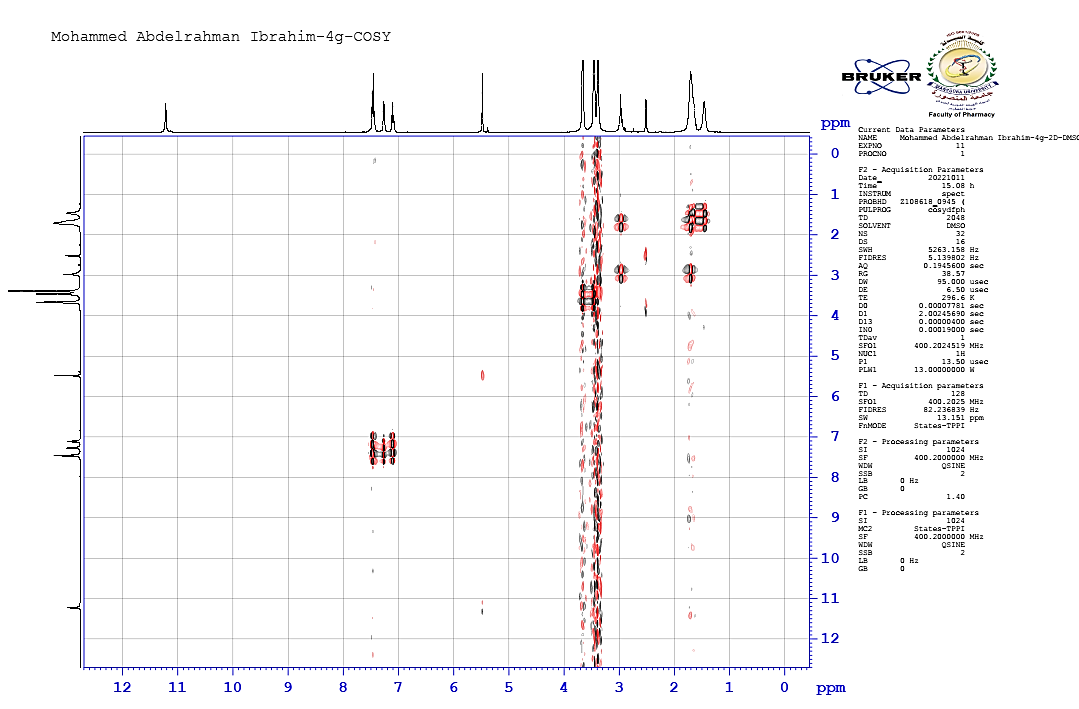
**

**Figure 25.** ^1^H-^1^H Cosy spectrum for compound **4g.**


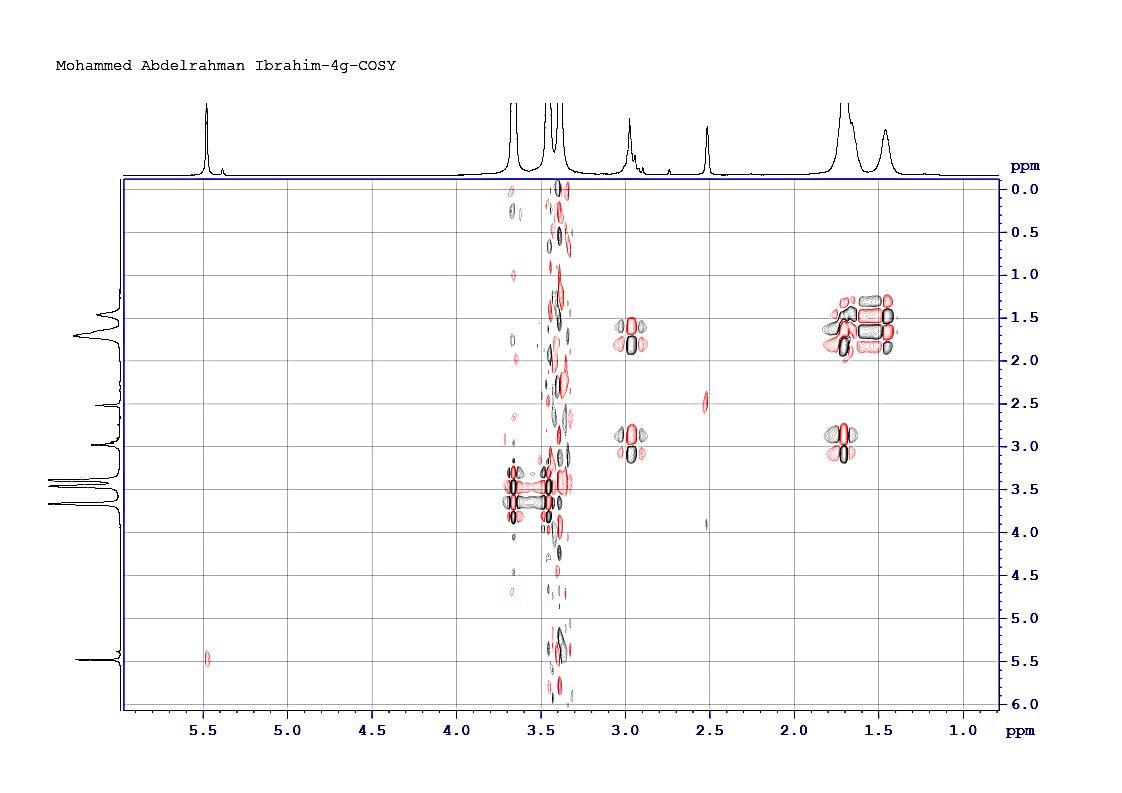


**Figure 26.** Part of ^1^H-^1^H Cosy spectrum for compound **4g.**


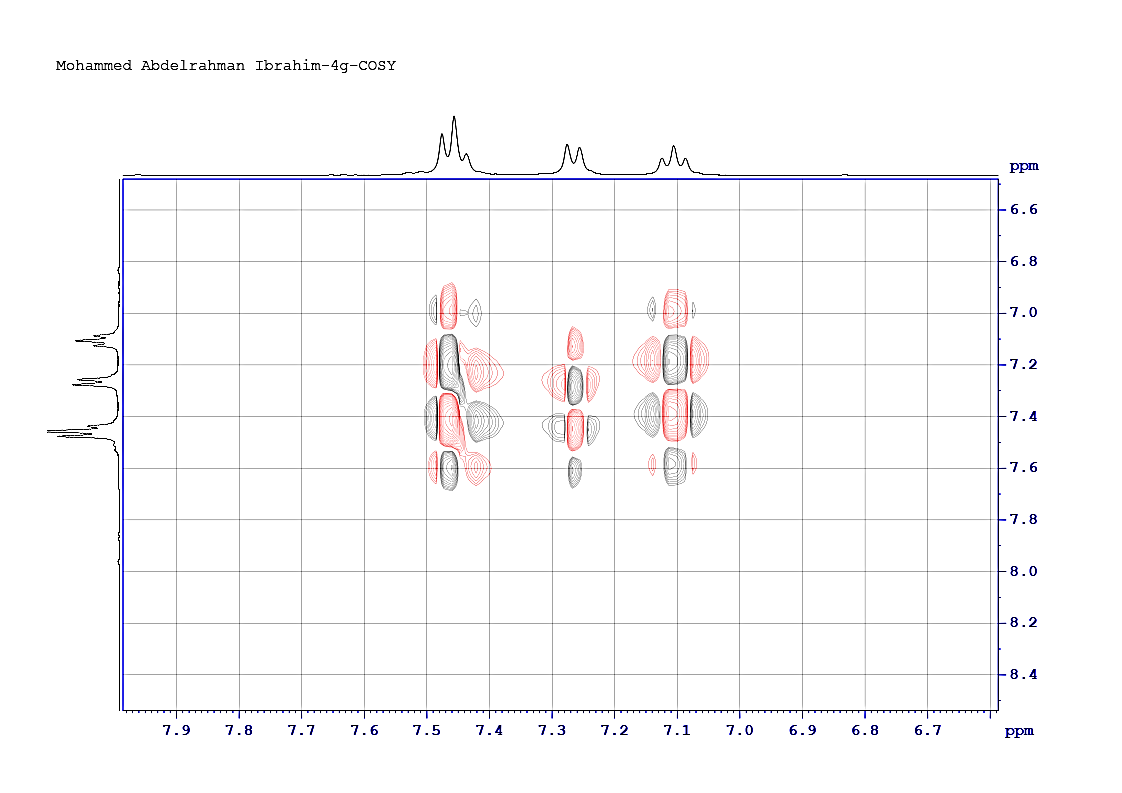


**Figure 27.** Part of ^1^H-^1^H Cosy spectrum for compound **4g.**


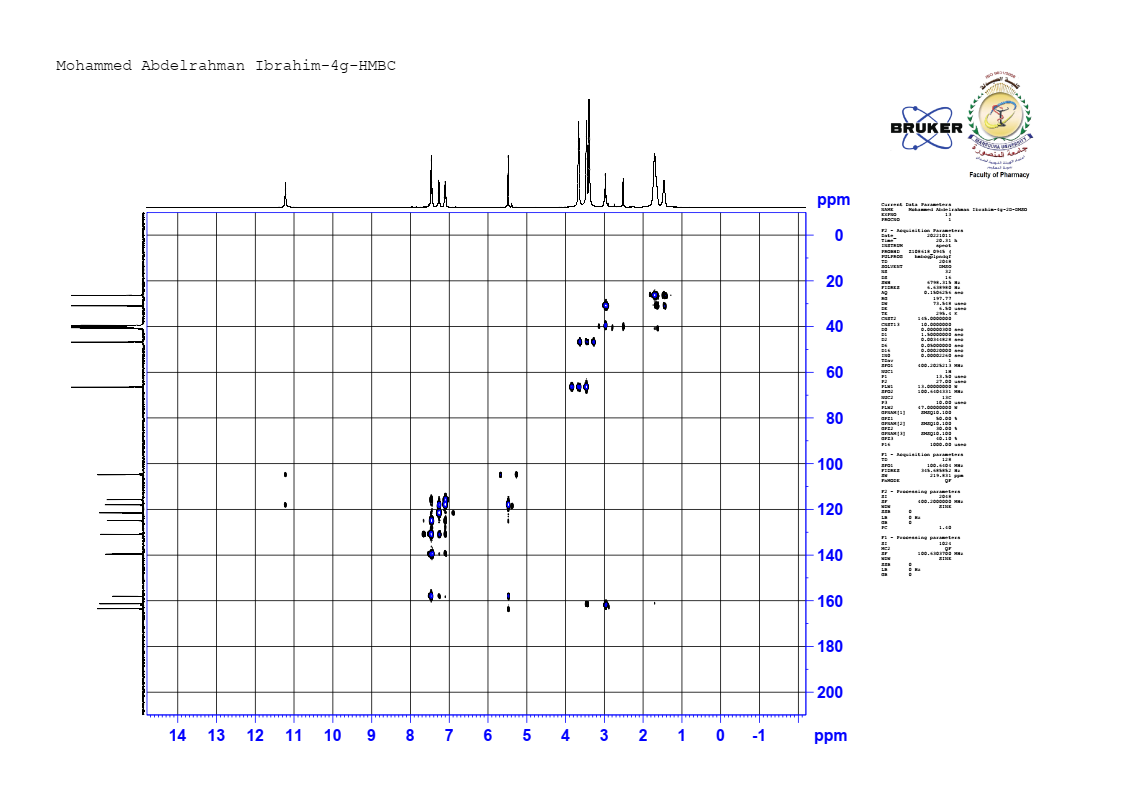


**Figure 28.** HMBC spectrum for compound **4g.**


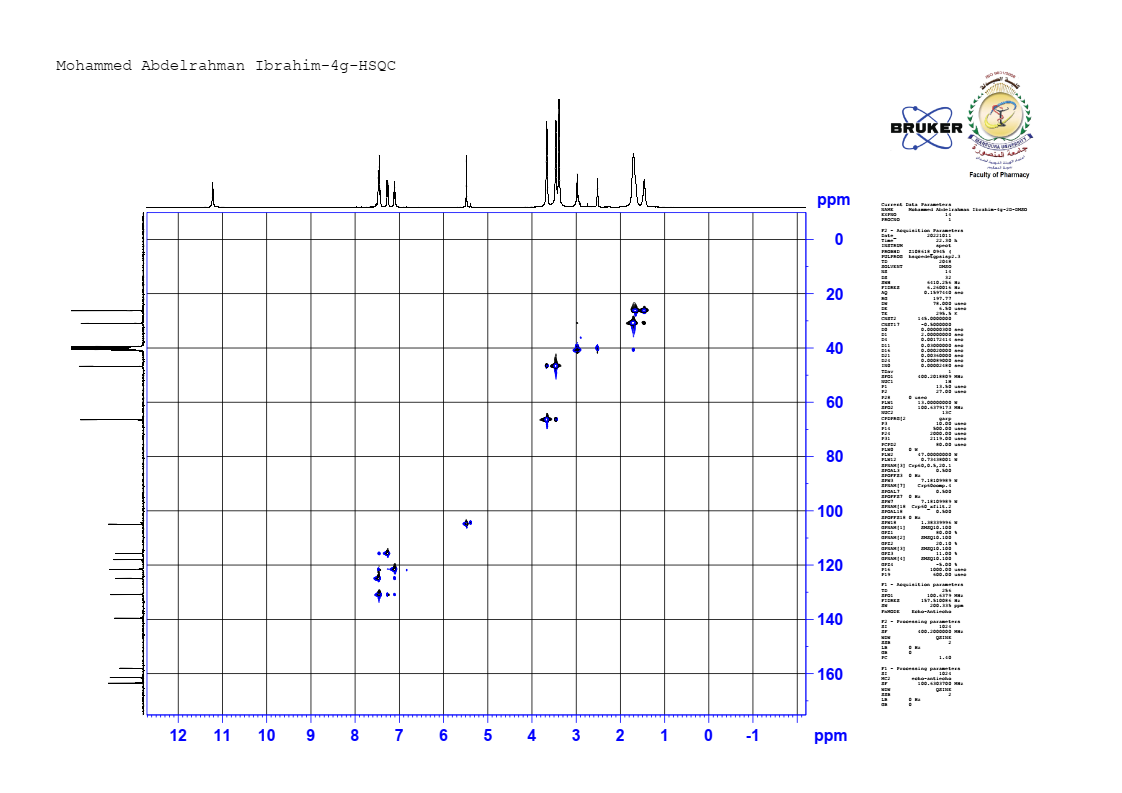


**Figure 29.** HSQC spectrum for compound **4g.**

**
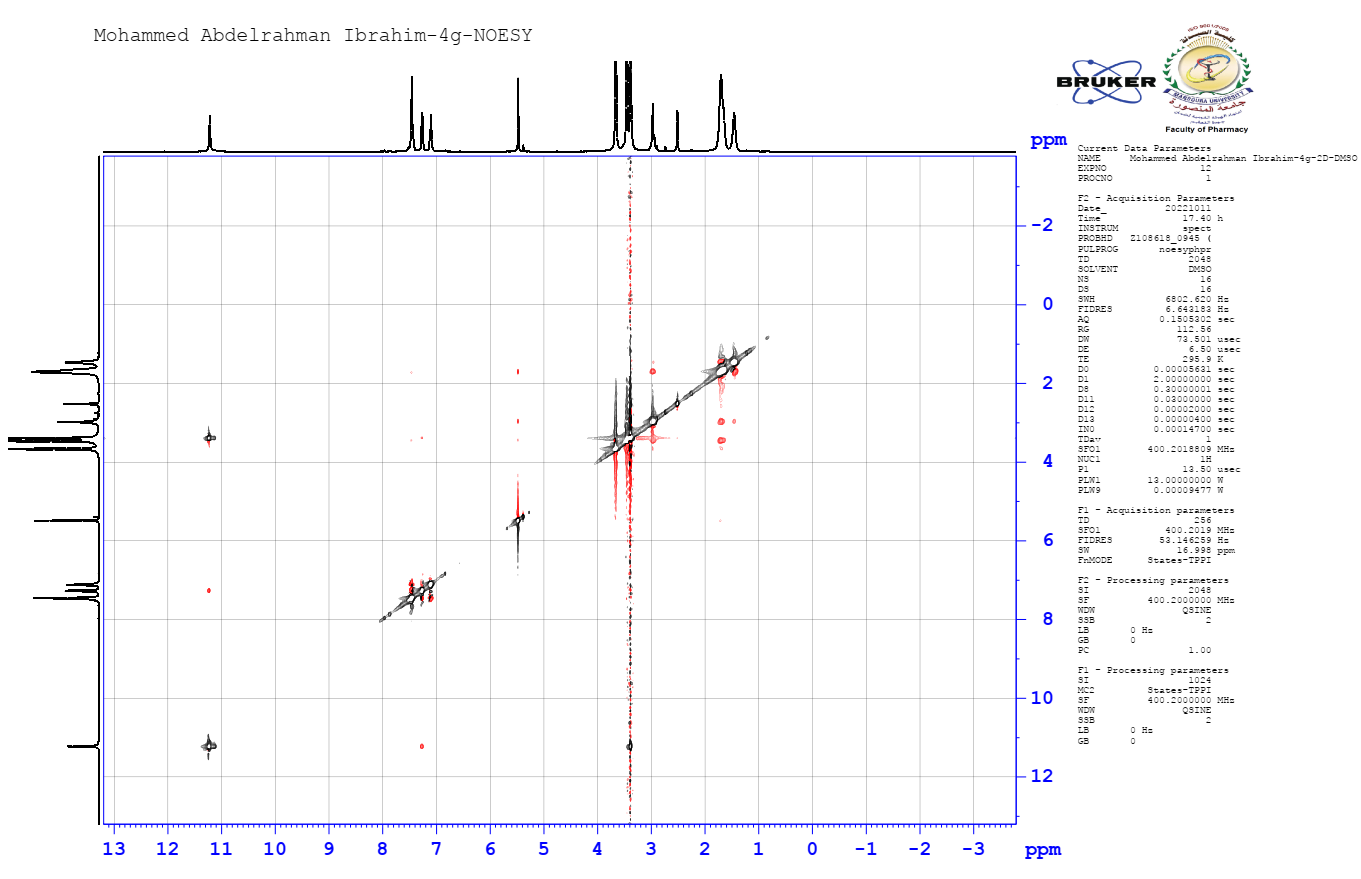
**

**Figure 30.** NOESY spectrum for compound **4g.**

**
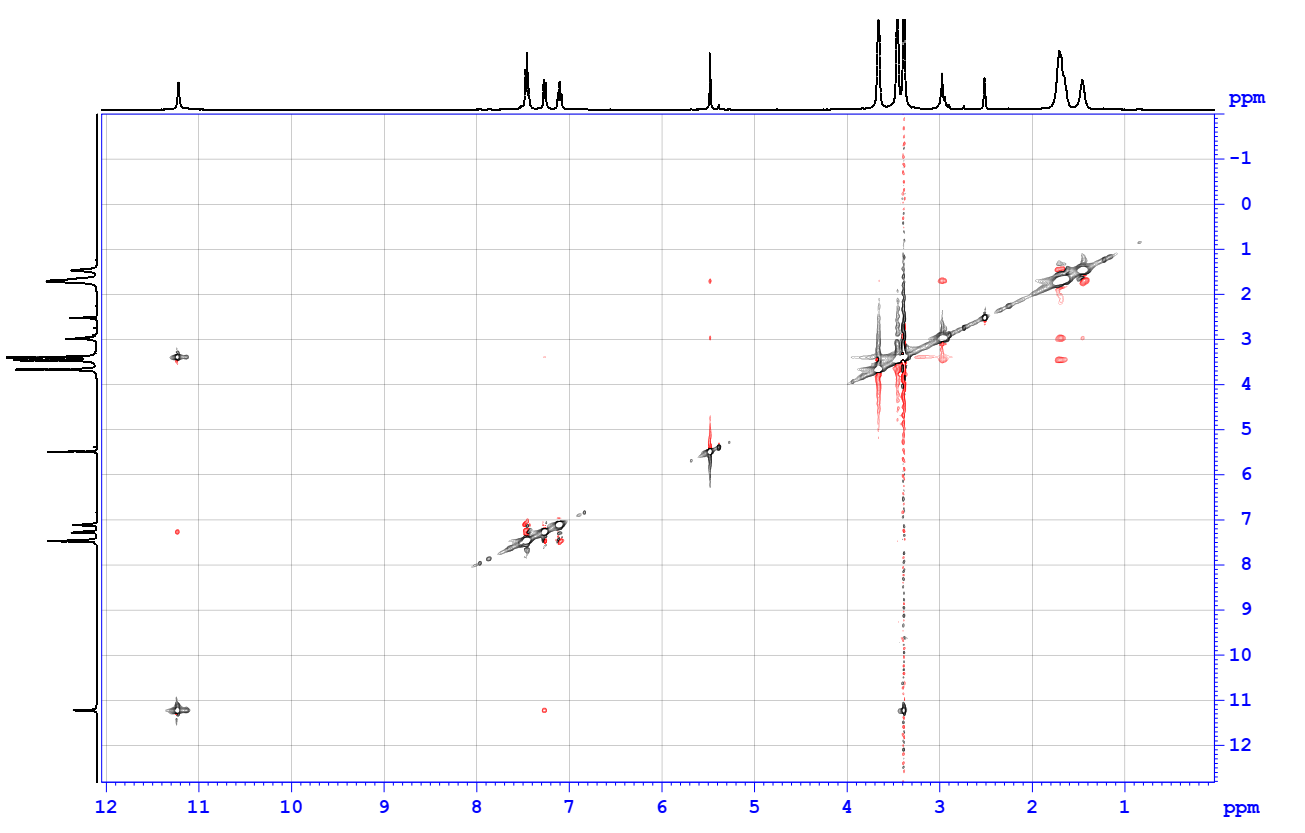
**

**Figure 31.** Part NOESY spectrum for compound **4g.**

**
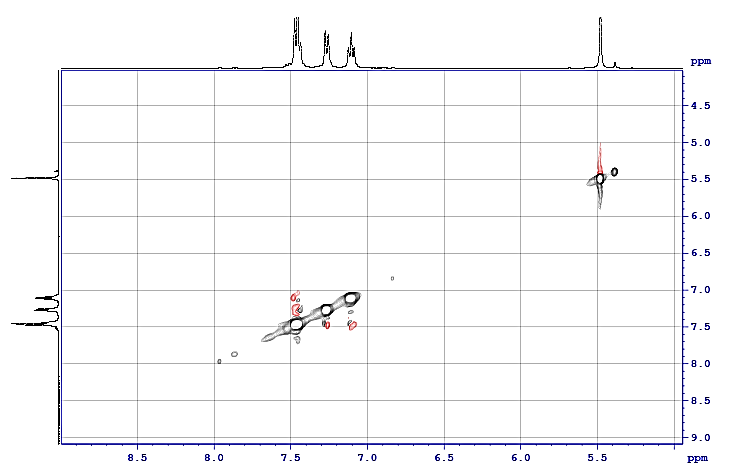
**

**Figure 32.** Part NOESY spectrum for compound **4g.**

**
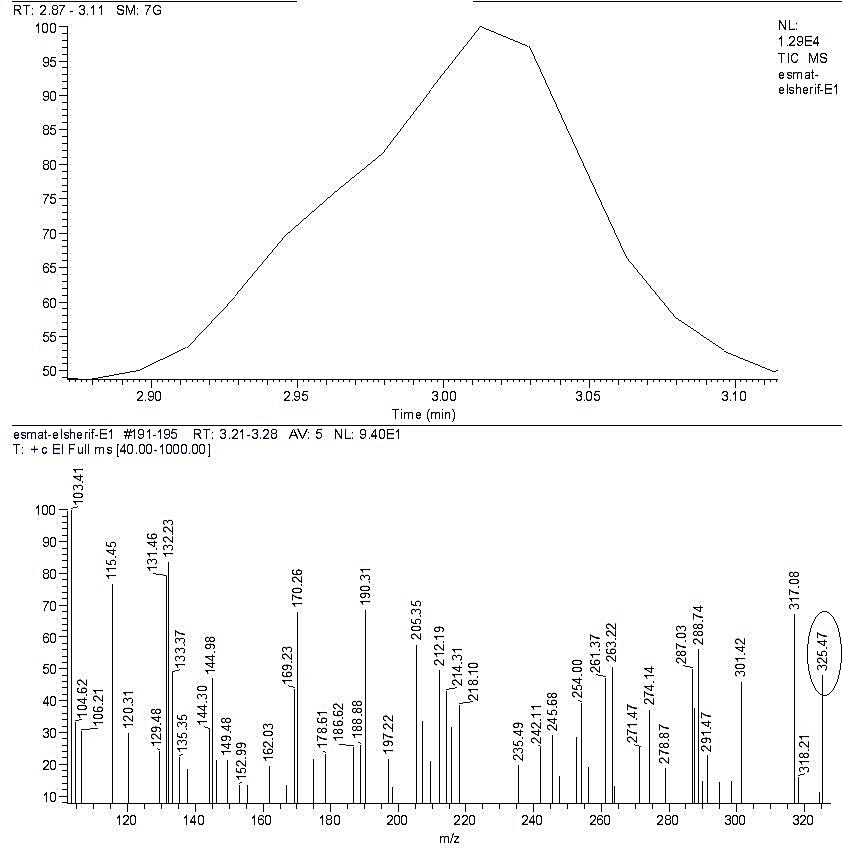
**

**Figure 33.** Mass spectrometry for compound **4g.**

**Spectral data for compound 4h.**

**
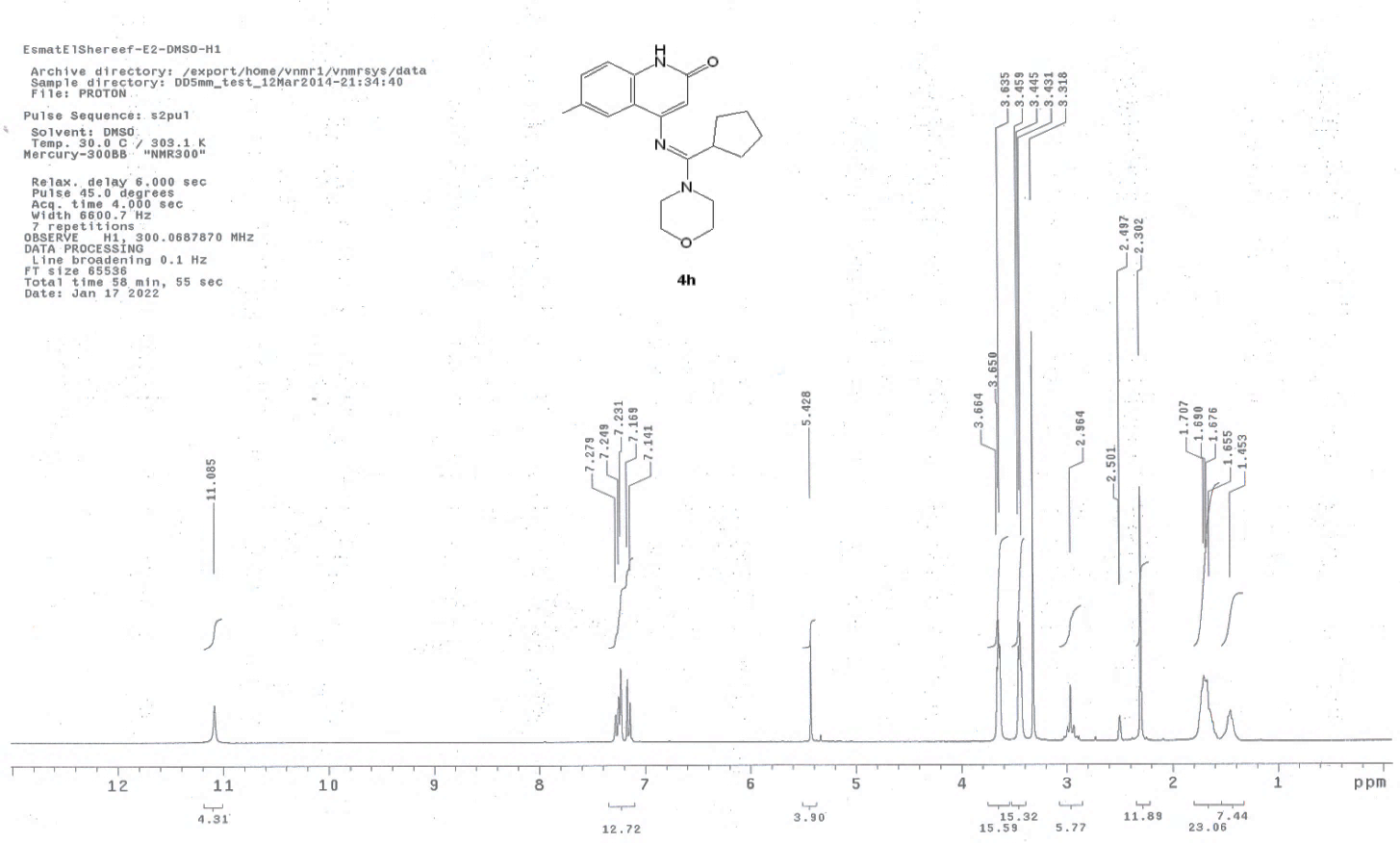
**

**Figure 34.** ^1^H-NMR spectrums for compound **4h.**

**
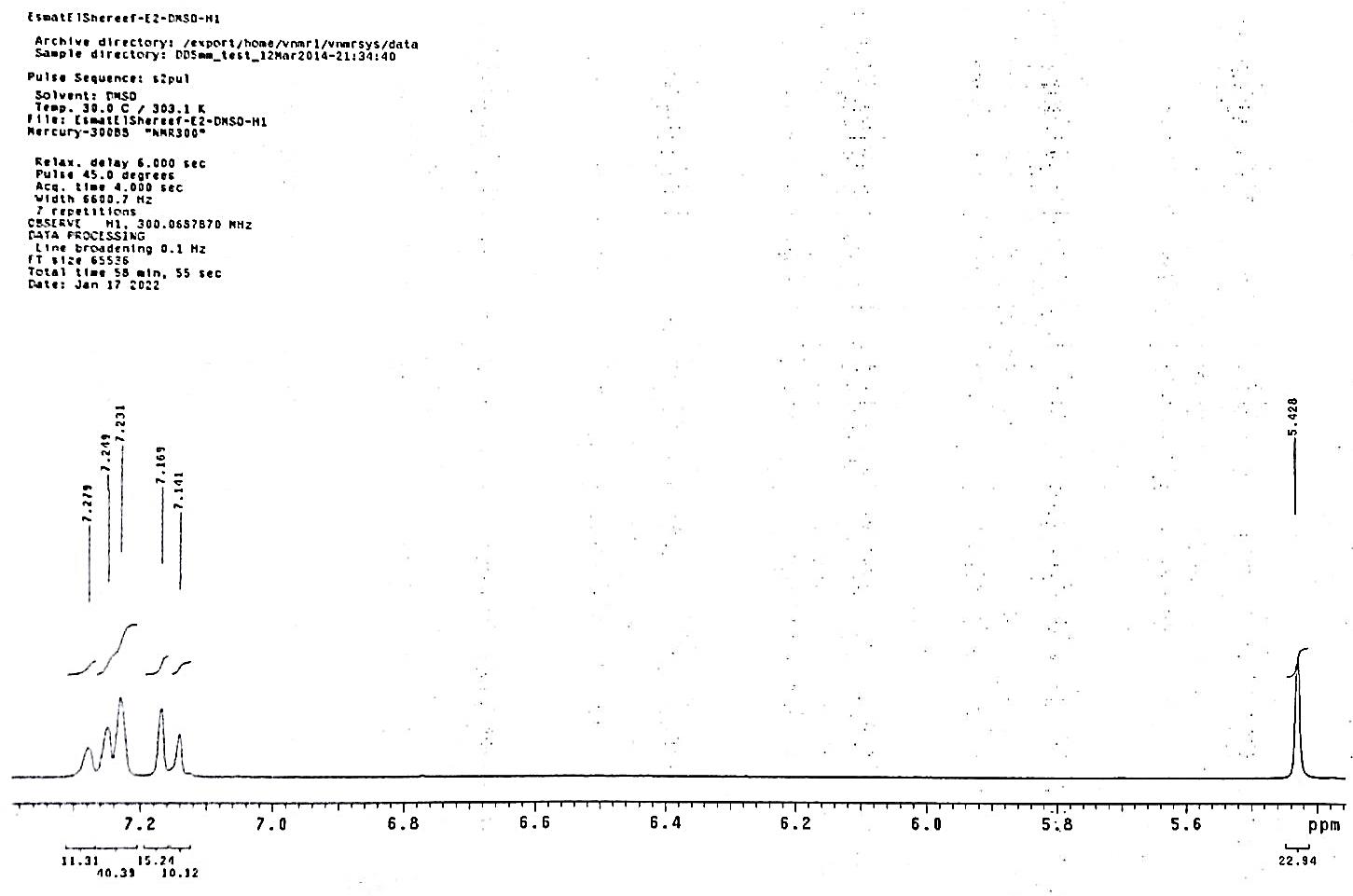
**

**Figure 35.** Part of the ^1^H-NMR spectrums for compound **4h.**

**
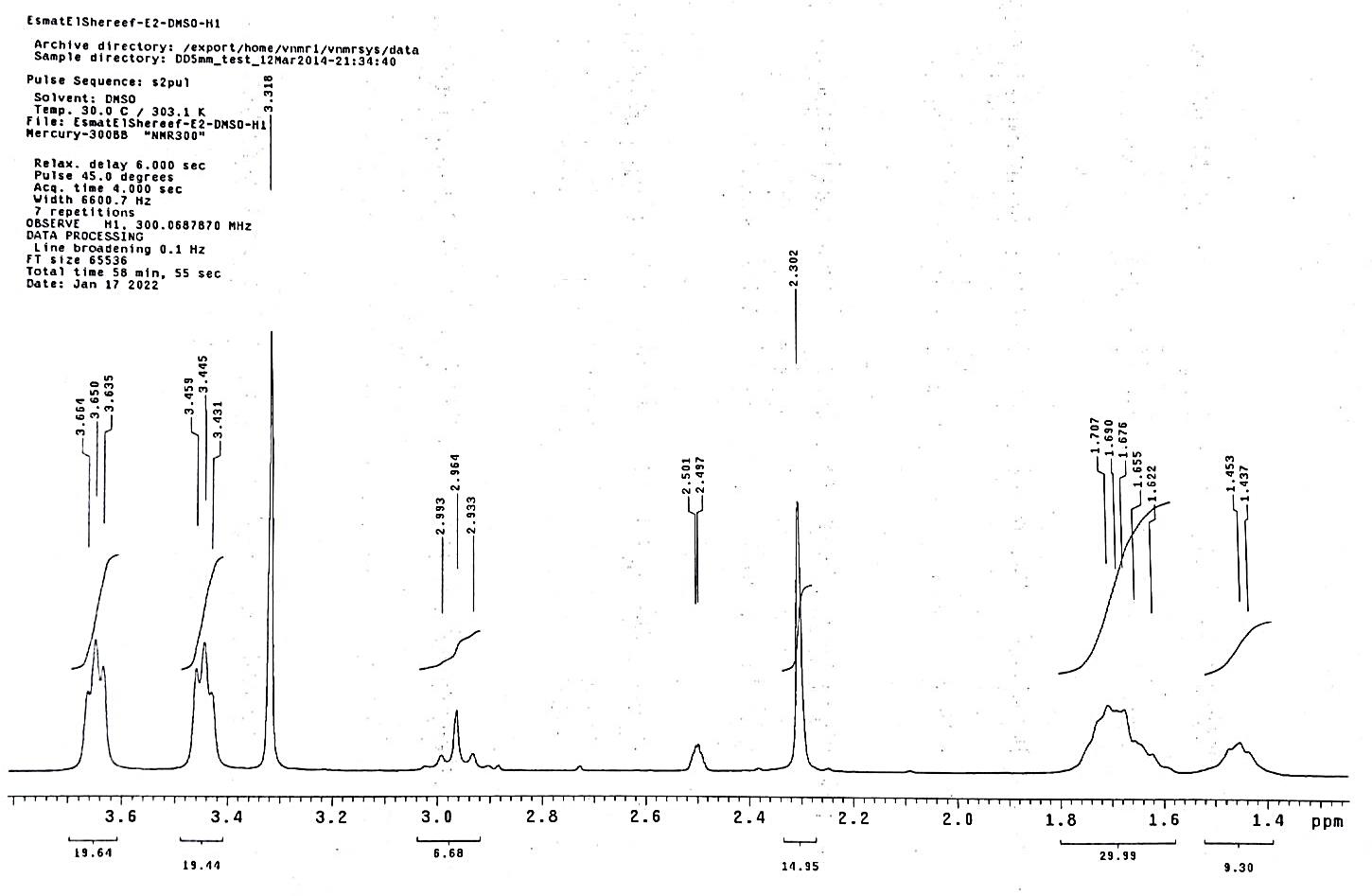
**

**Figure 36.** Part of the ^1^H-NMR spectrums for compound **4h.**

**
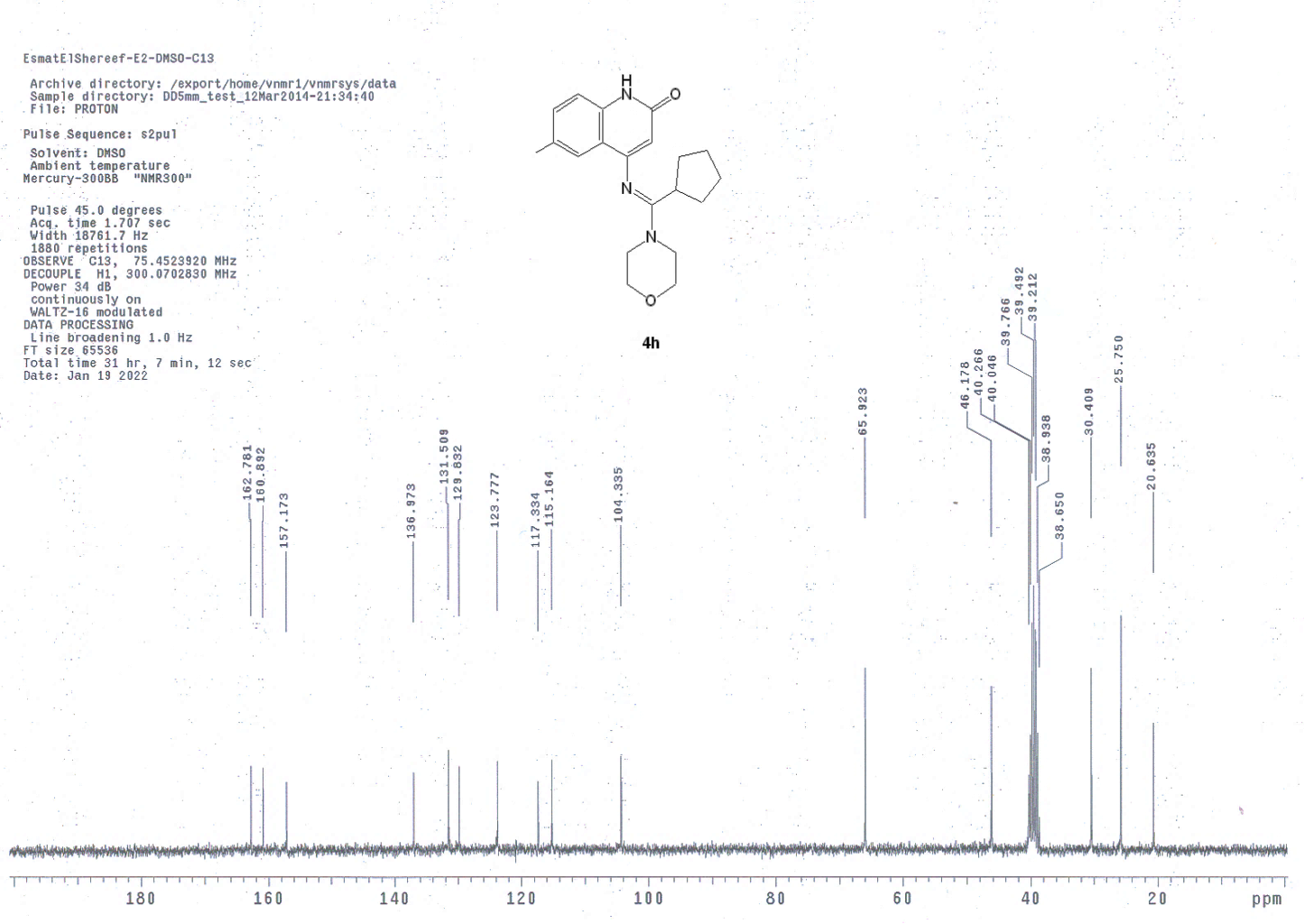
**

**Figure 37.** ^13^C-NMR spectrums for compound **4h.**

**Spectral data for compound 4i.**

**
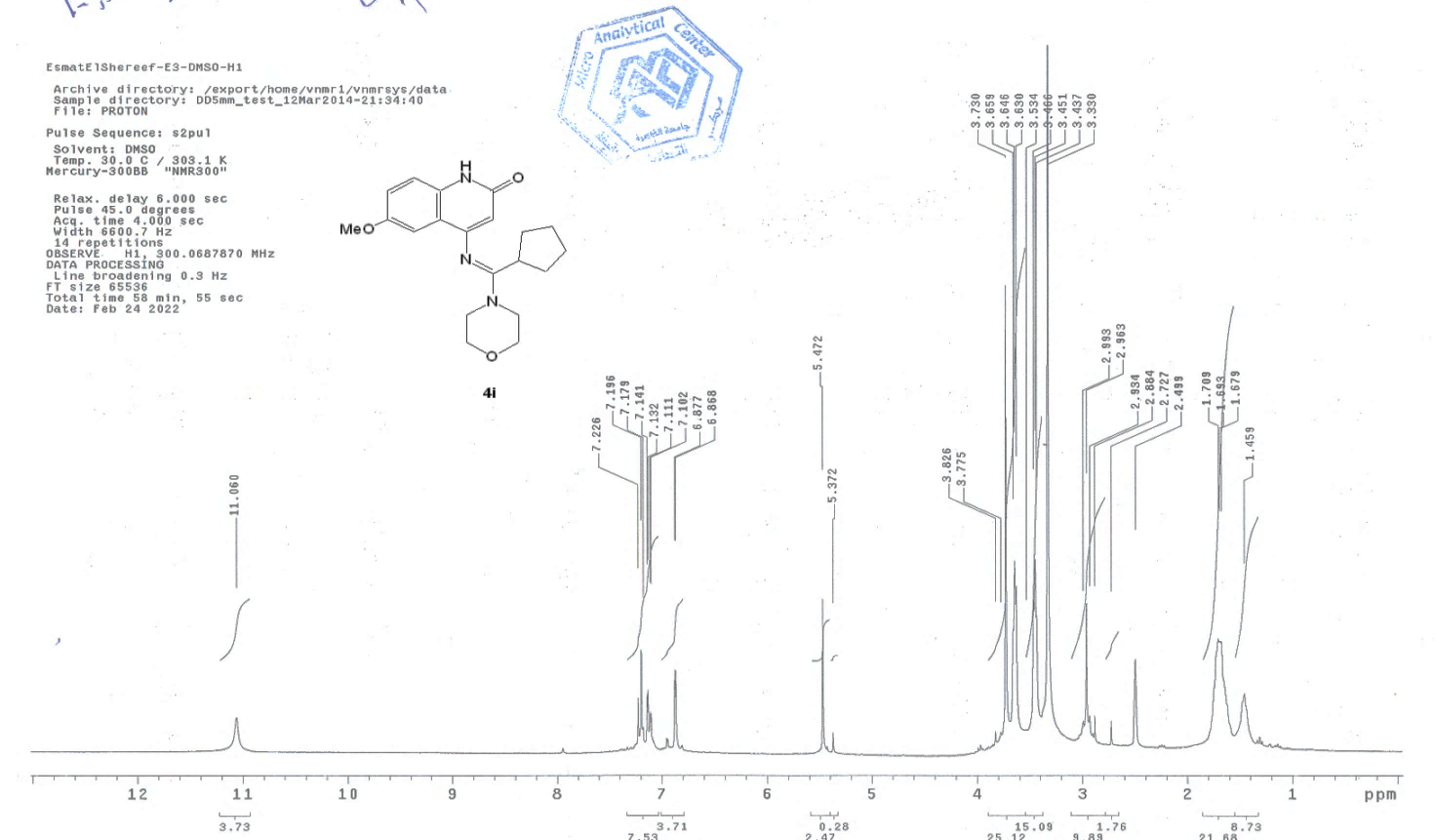
**

**Figure 38.** ^1^H-NMR spectrums for compound **4i.**

**
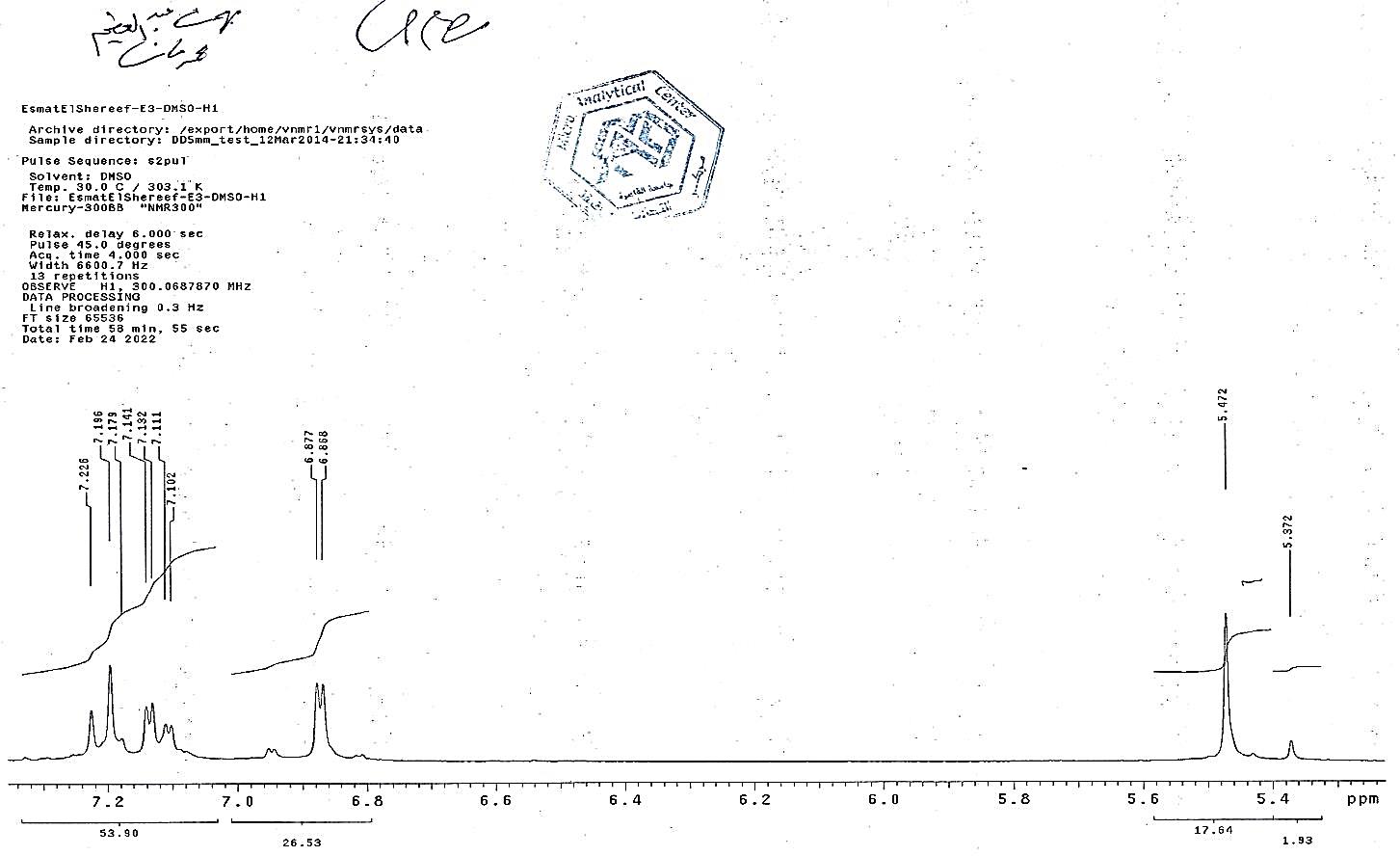
**

**Figure 39.** Part of the ^1^H-NMR spectrums for compound **4i.**

**
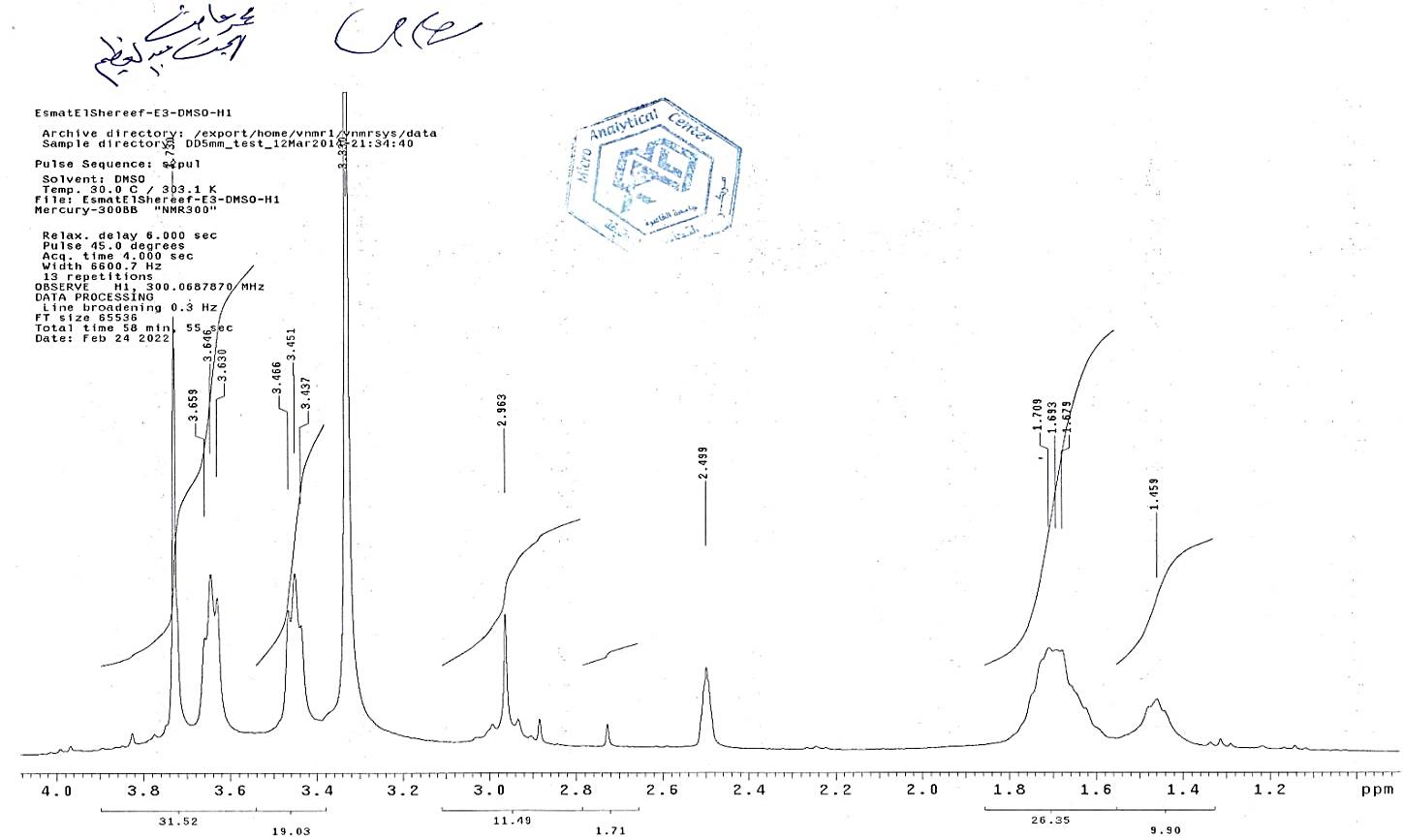
**

**Figure 40.** Part of the ^1^H-NMR spectrums for compound **4i.**

**Spectral data for compound 4j.**

**
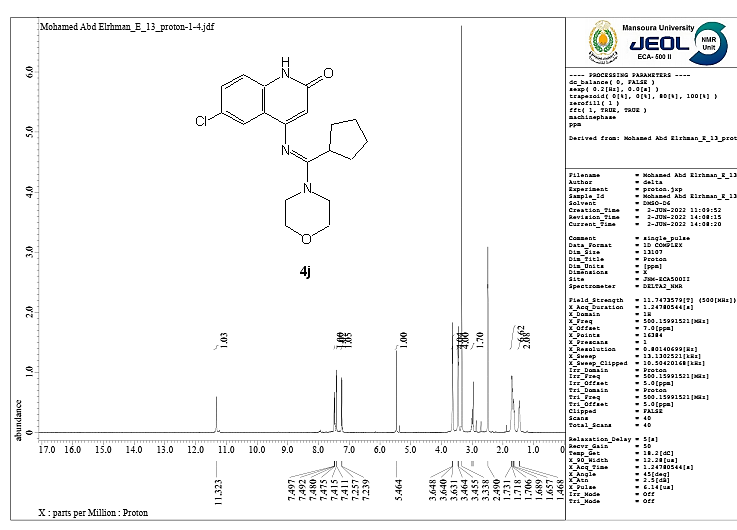
**

**Figure 41.** ^1^H-NMR spectrums for compound **4j.**

**
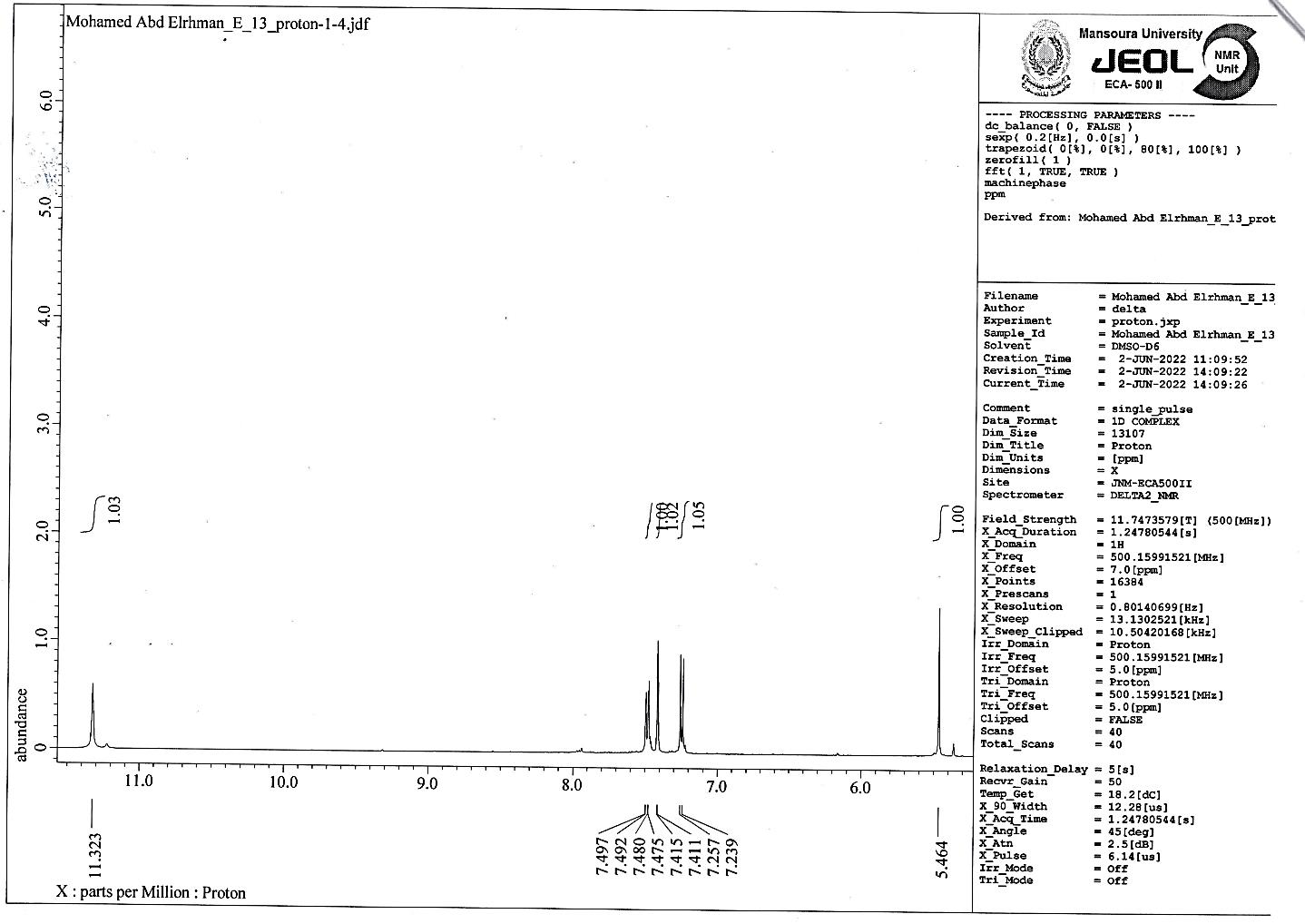
**

**Figure 42.** Part of the ^1^H-NMR spectrums for compound **4j.**

**`
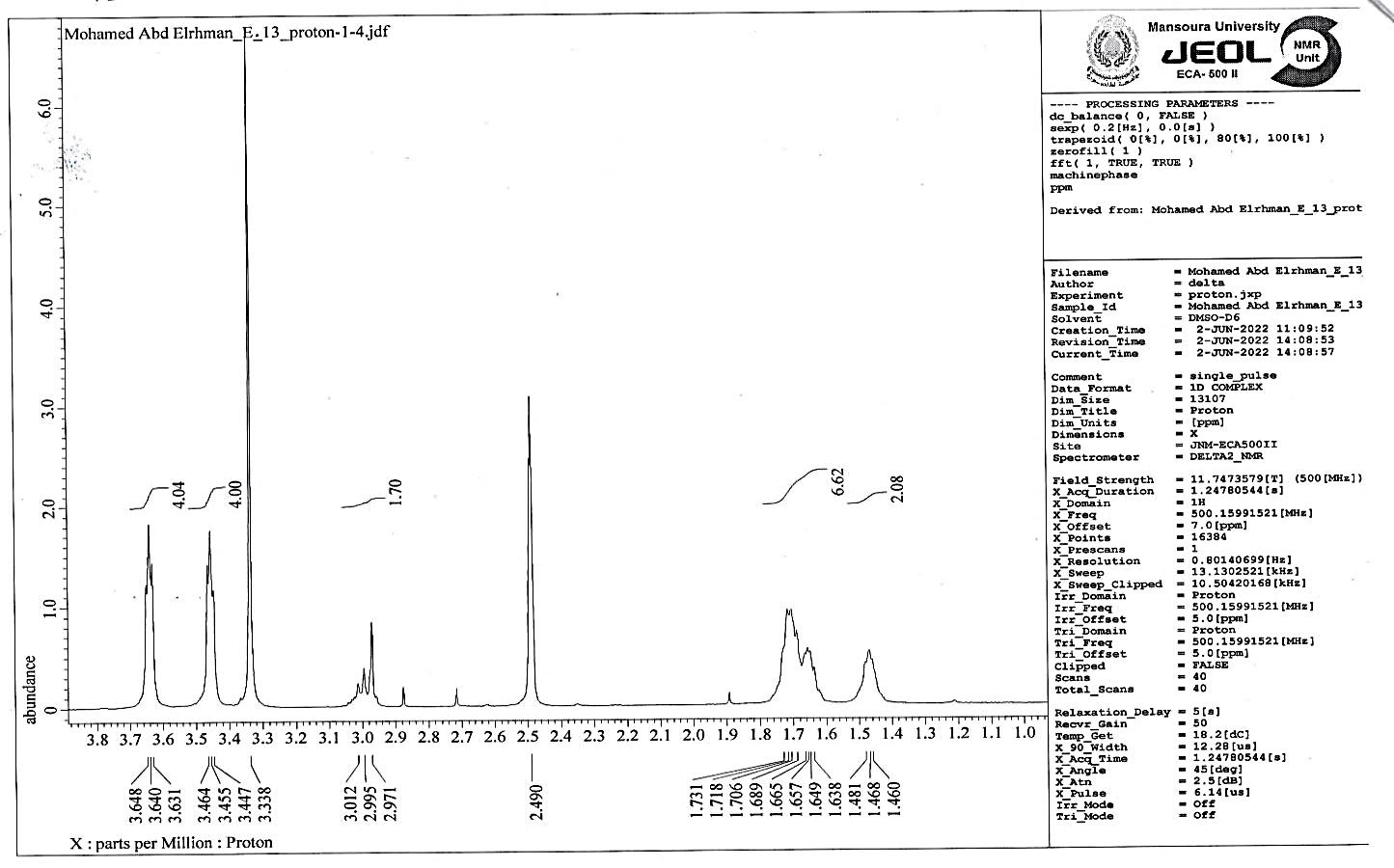
**

**Figure 43.** Part of the ^1^H-NMR spectrums for compound **4j.**


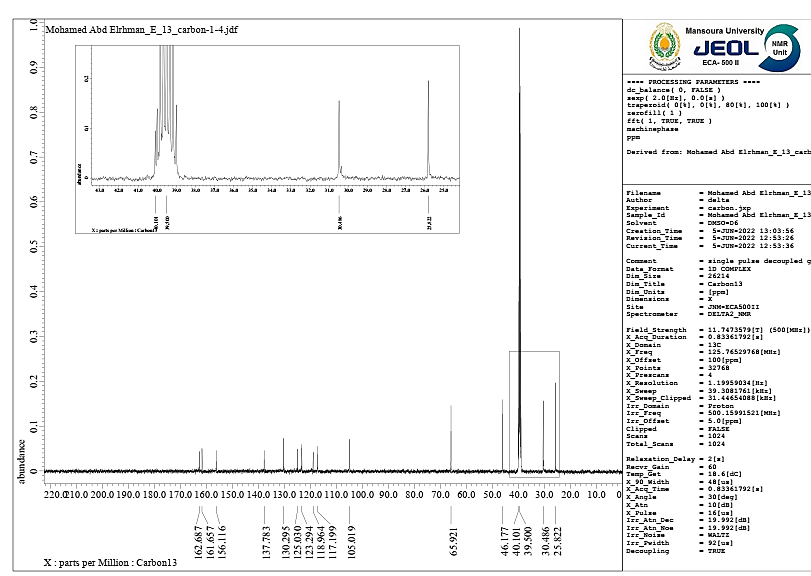


**Figure 44.** ^13^C-NMR spectrums for compound **4j.**

**Spectral data for compound 4k.**

**
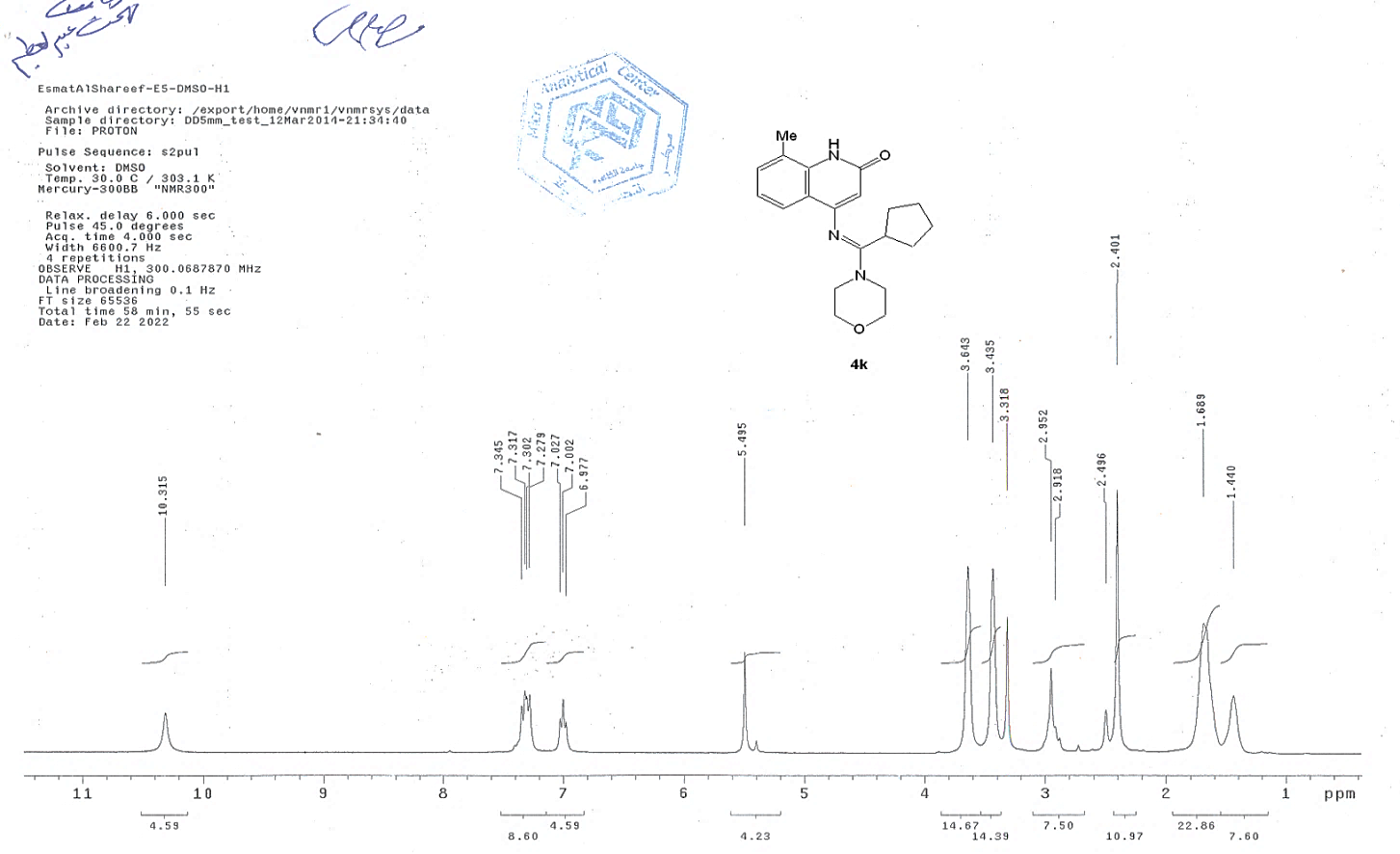
**

**Figure 45.** ^1^H-NMR spectrums for compound **4K.**

**
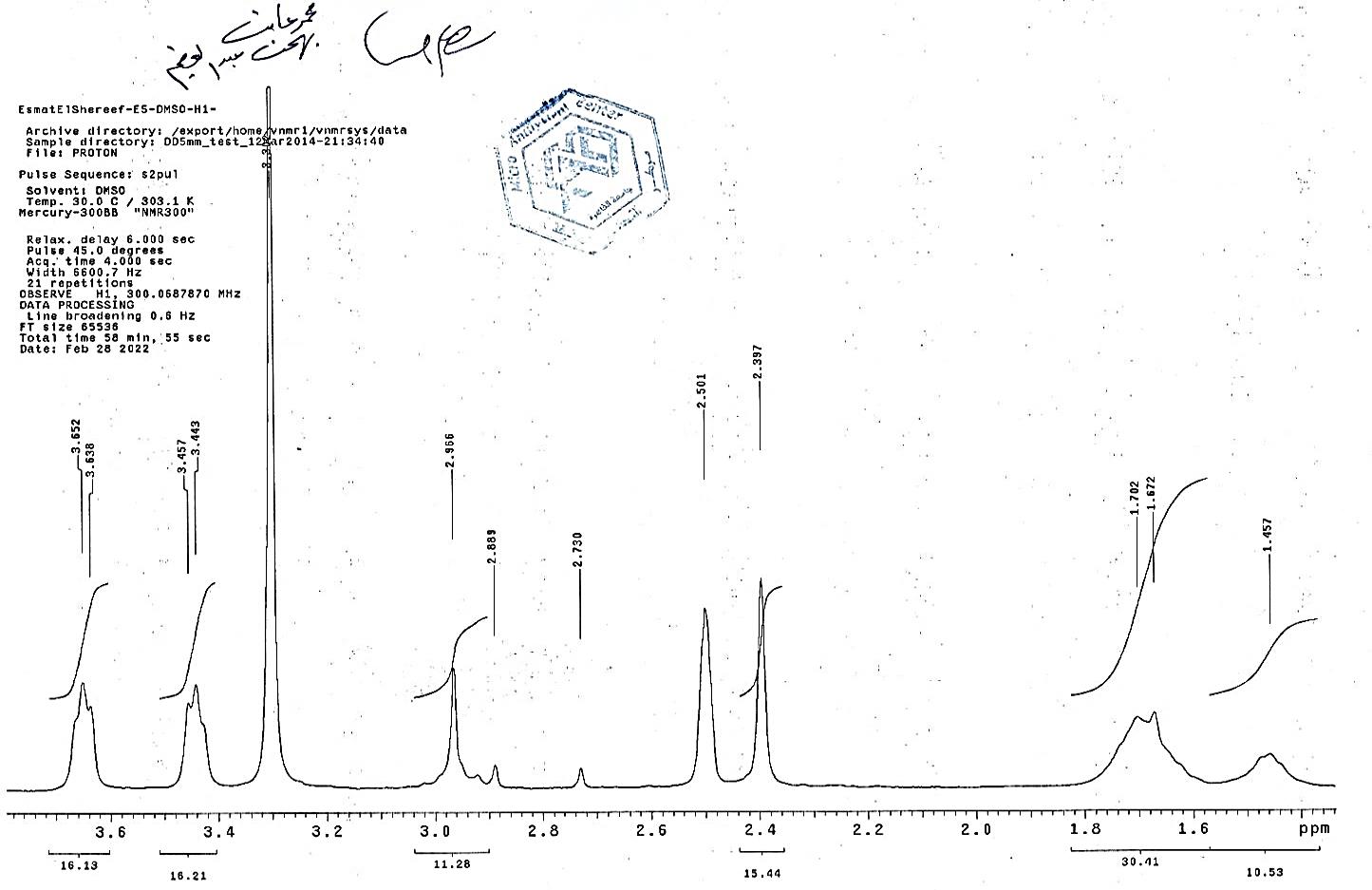
**

**Figure 46.** Part of the ^1^H-NMR spectrums for compound **4K.**

**
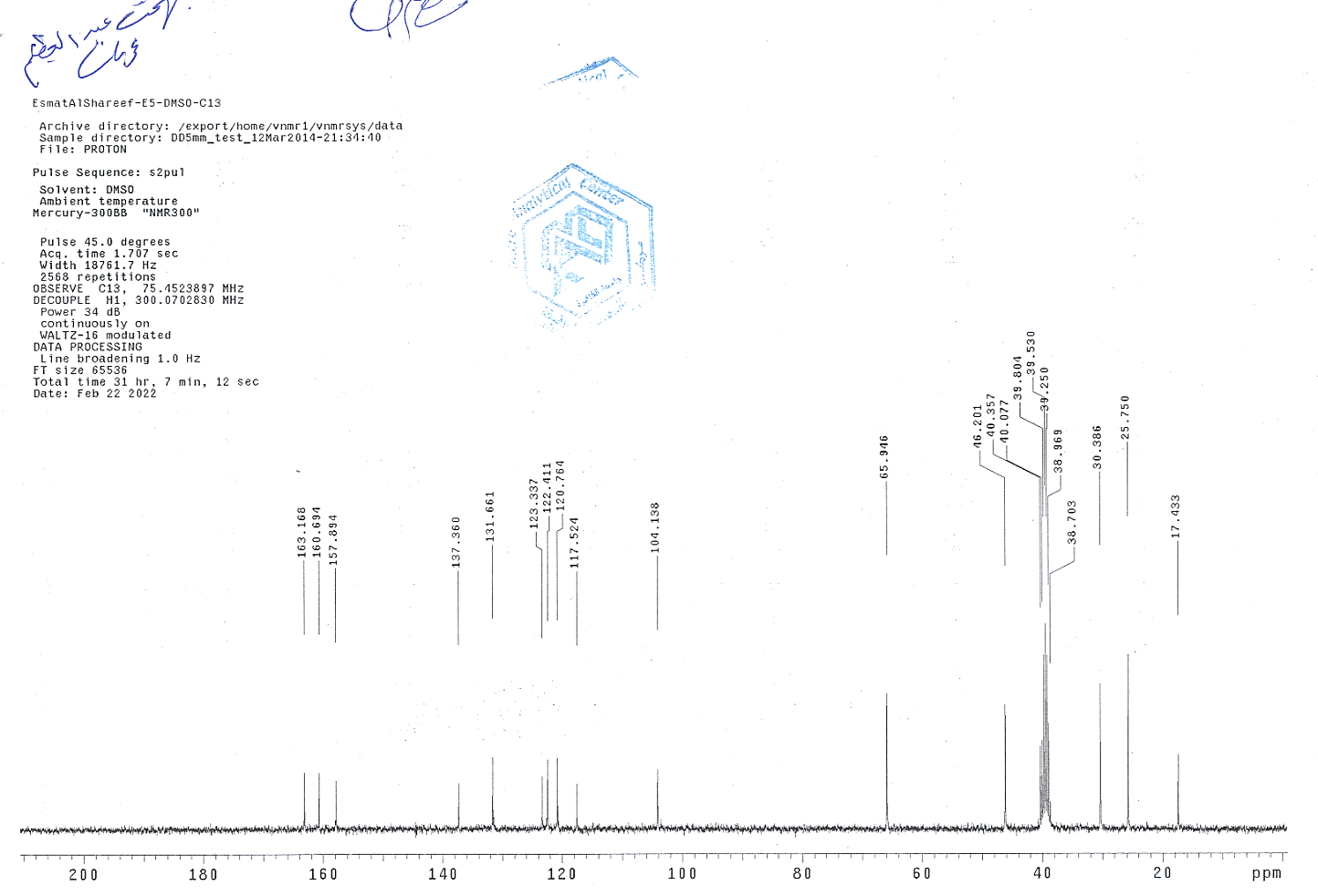
**

**Figure 47.** ^13^C-NMR spectrums for compound **4K.**

**Spectral data for compound 4l.**

**
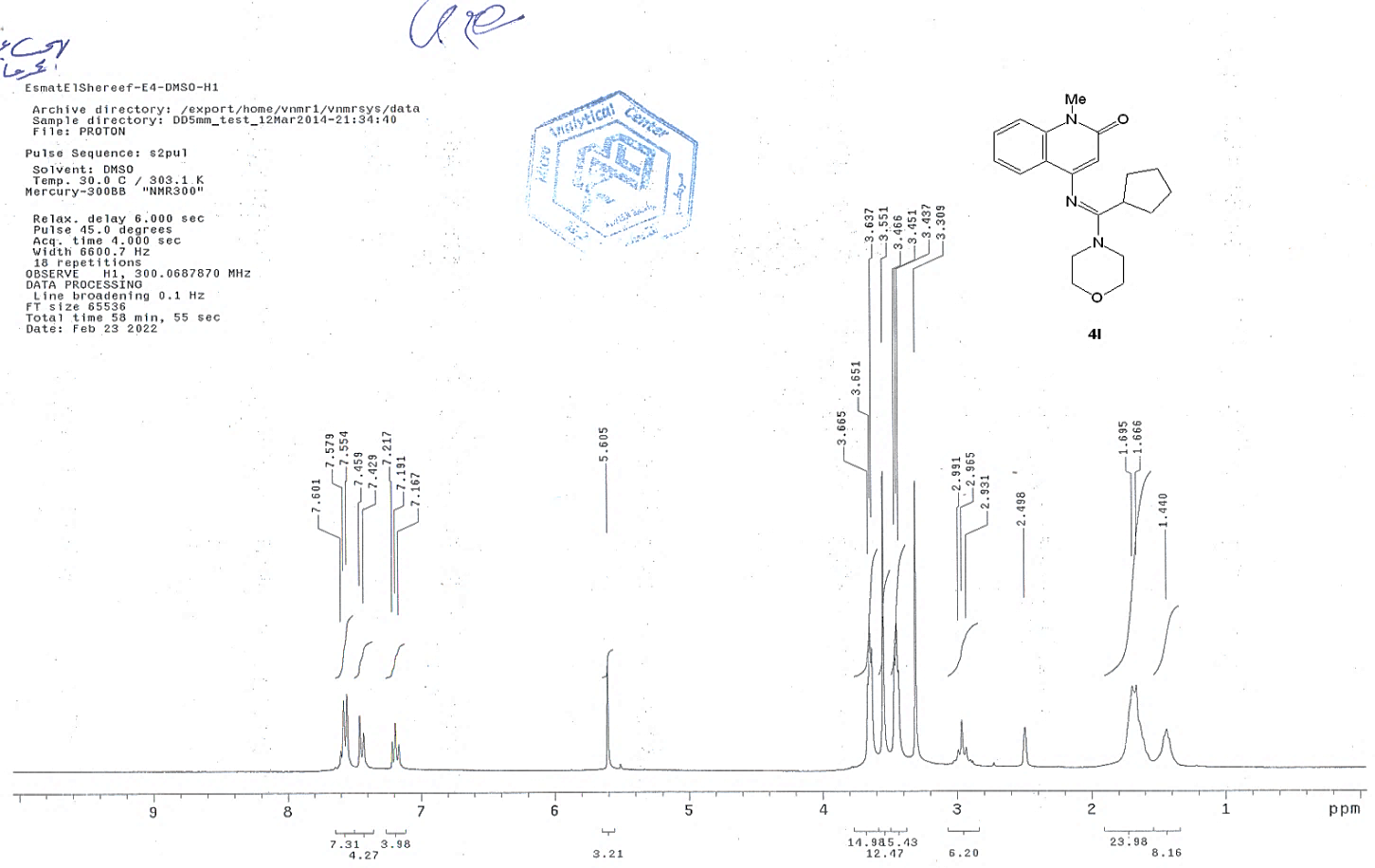
**

**Figure 48.** ^1^H-NMR spectrums for compound **4l.**

**
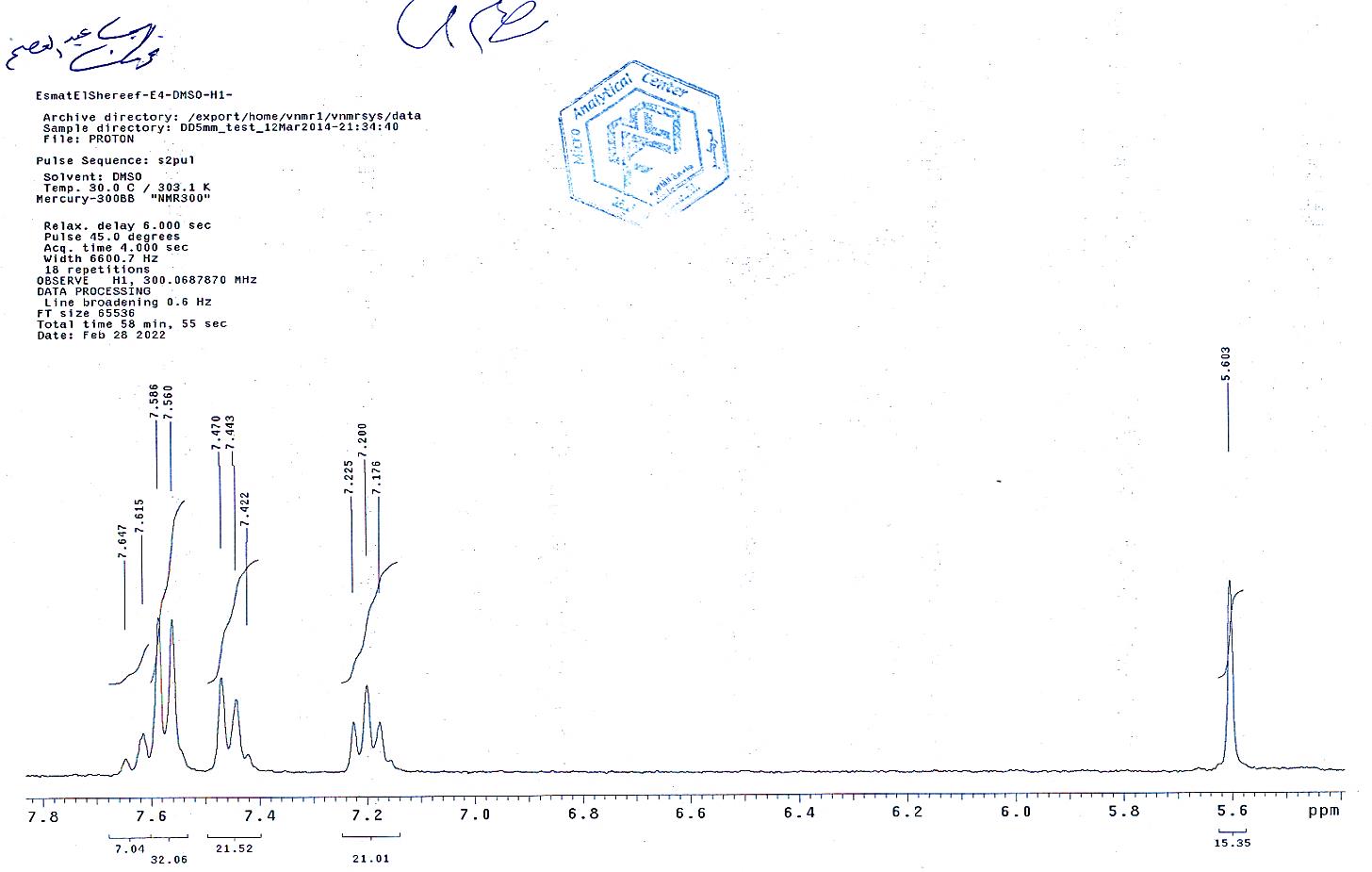
**

**Figure 49.** Part of the ^1^H-NMR spectrums for compound **4l.**

**
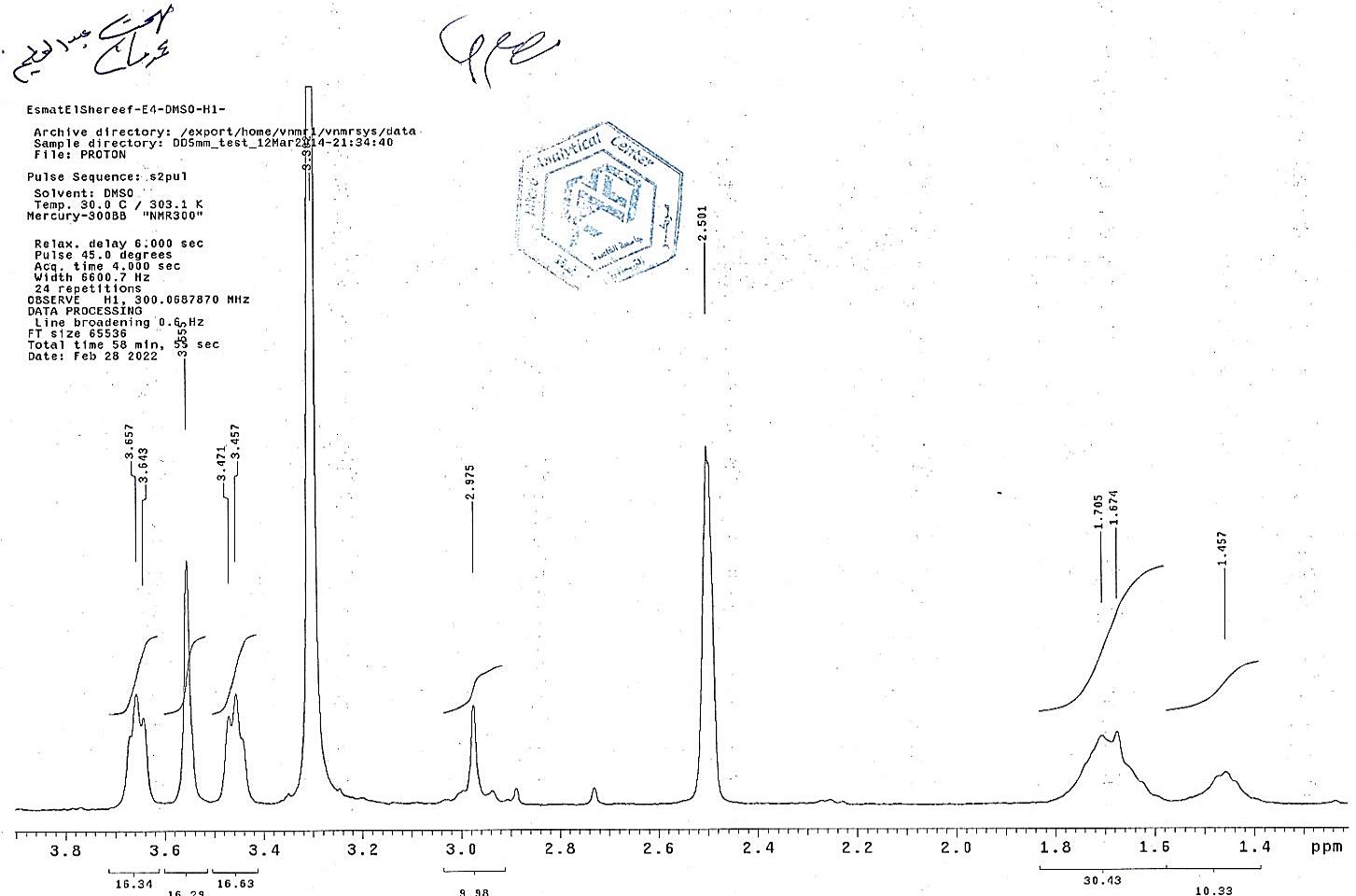
**

**Figure 50.** Part of the ^1^H-NMR spectrums for compound **4l.**

**
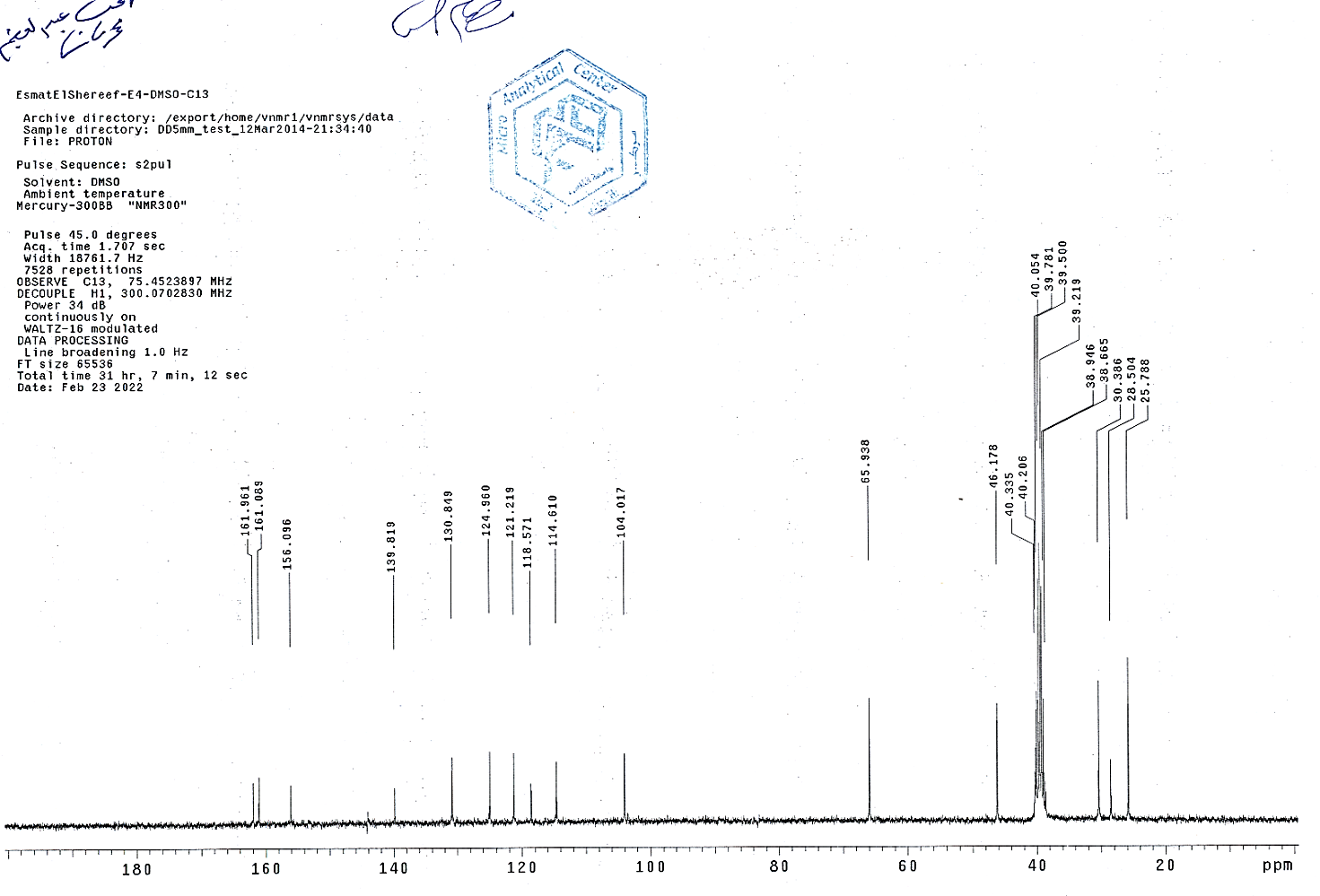
**

**Figure 51.** ^13^C-NMR spectrums for compound **4l.**

**Spectral data for compound 5a.**

**
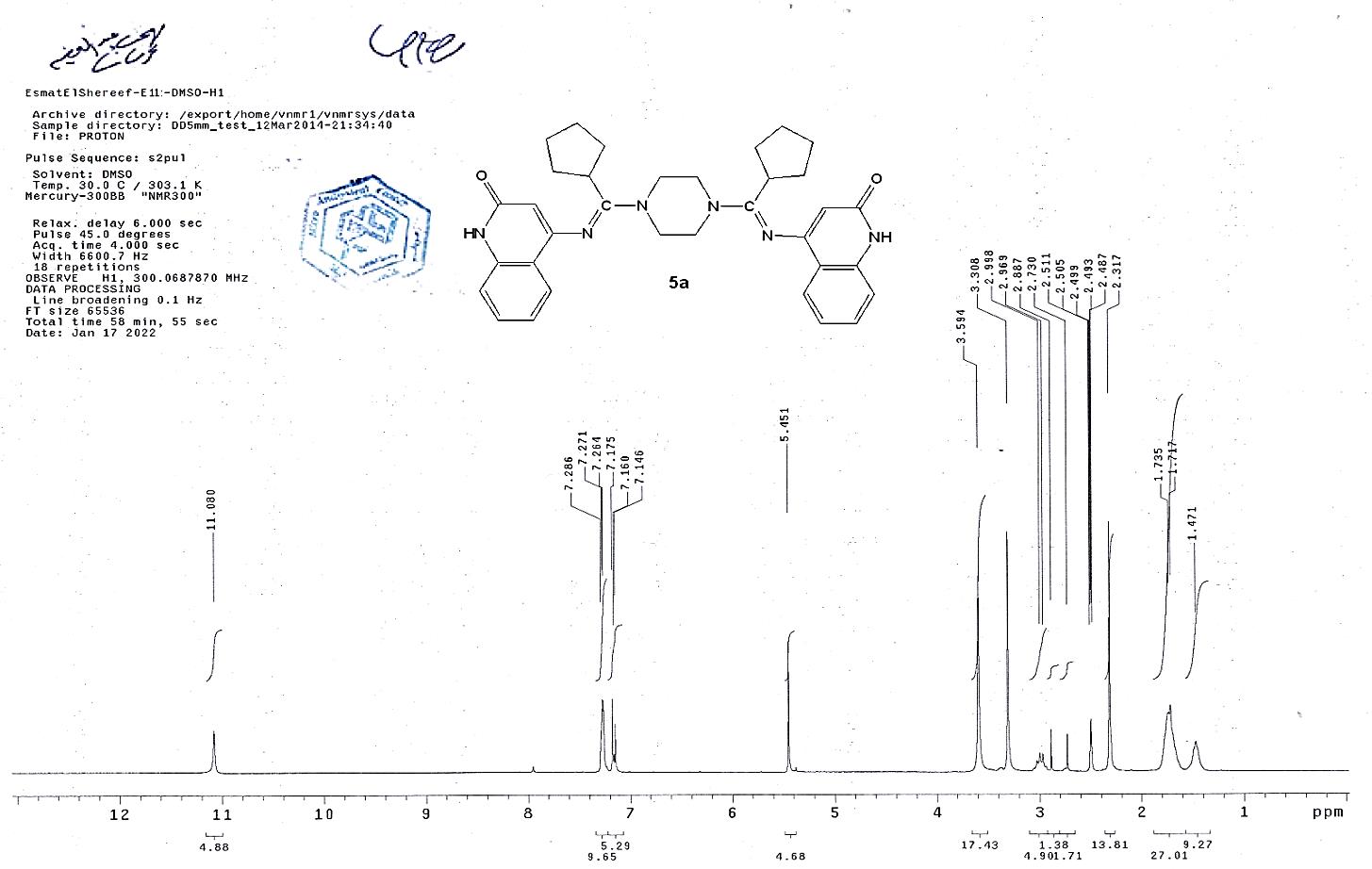
**

**Figure 52.** ^1^H-NMR spectrums for compound **5a.**

**
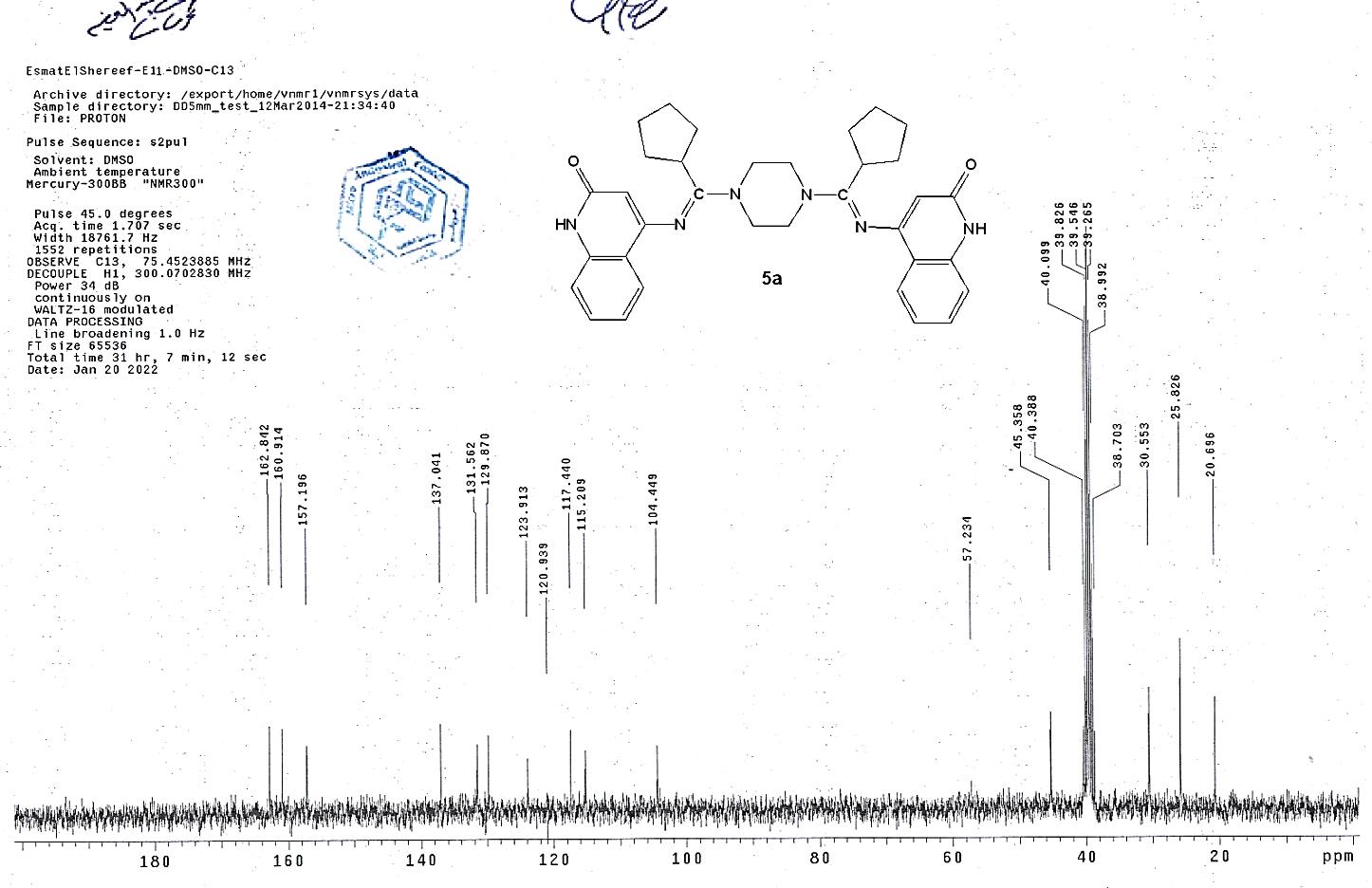
**

**Figure 53.** ^13^C-NMR spectrums for compound **5a.**

**
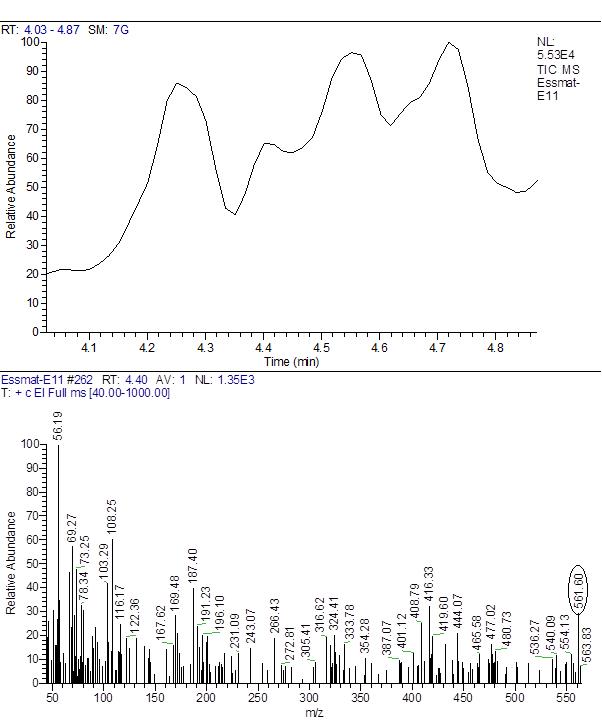
**

**Figure 54.** Mass spectrometry for compound **5a.**

**Spectral data for compound 5b.**

**
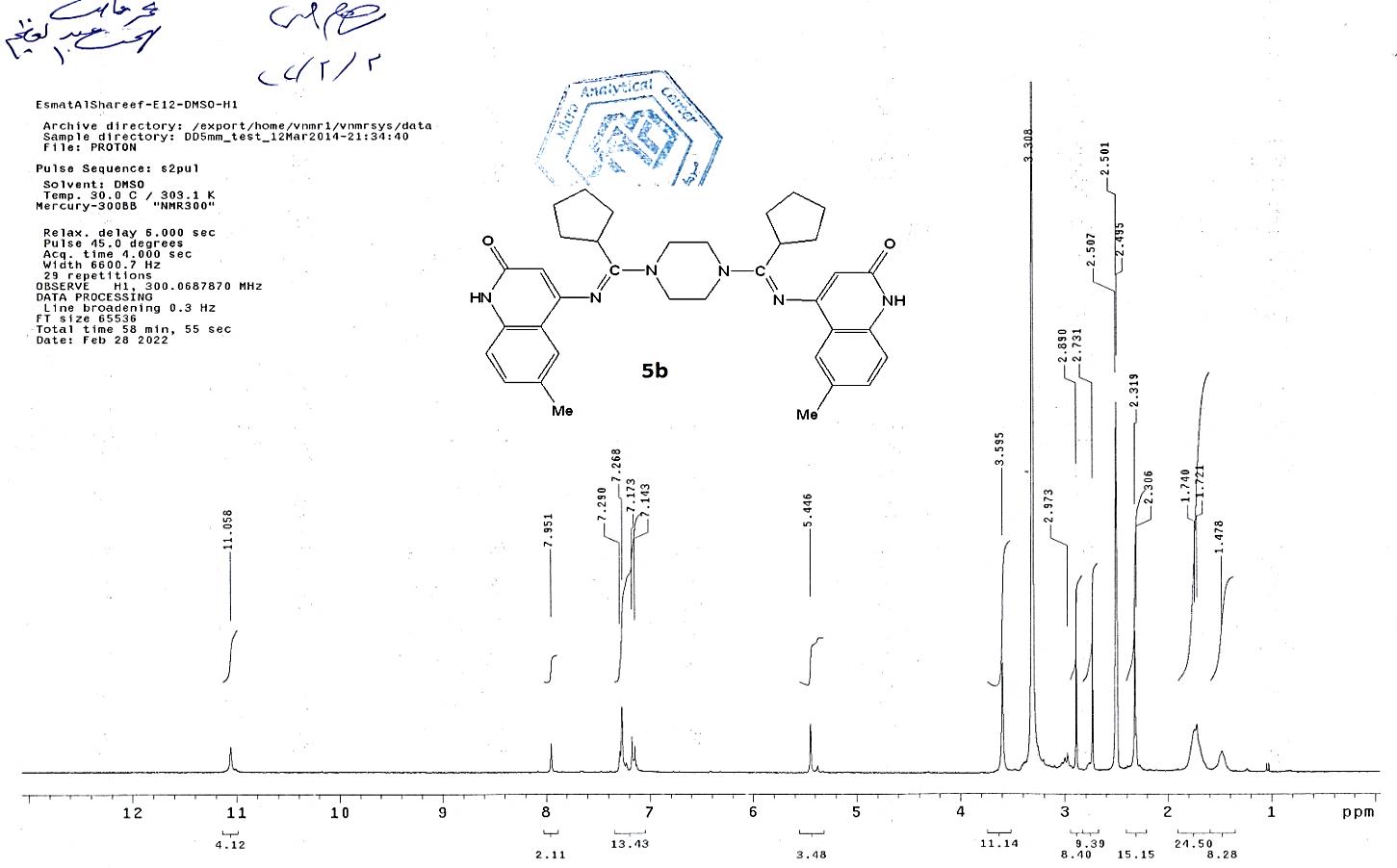
**

**Figure 55.** ^1^H-NMR spectrums for compound **5b.**

**
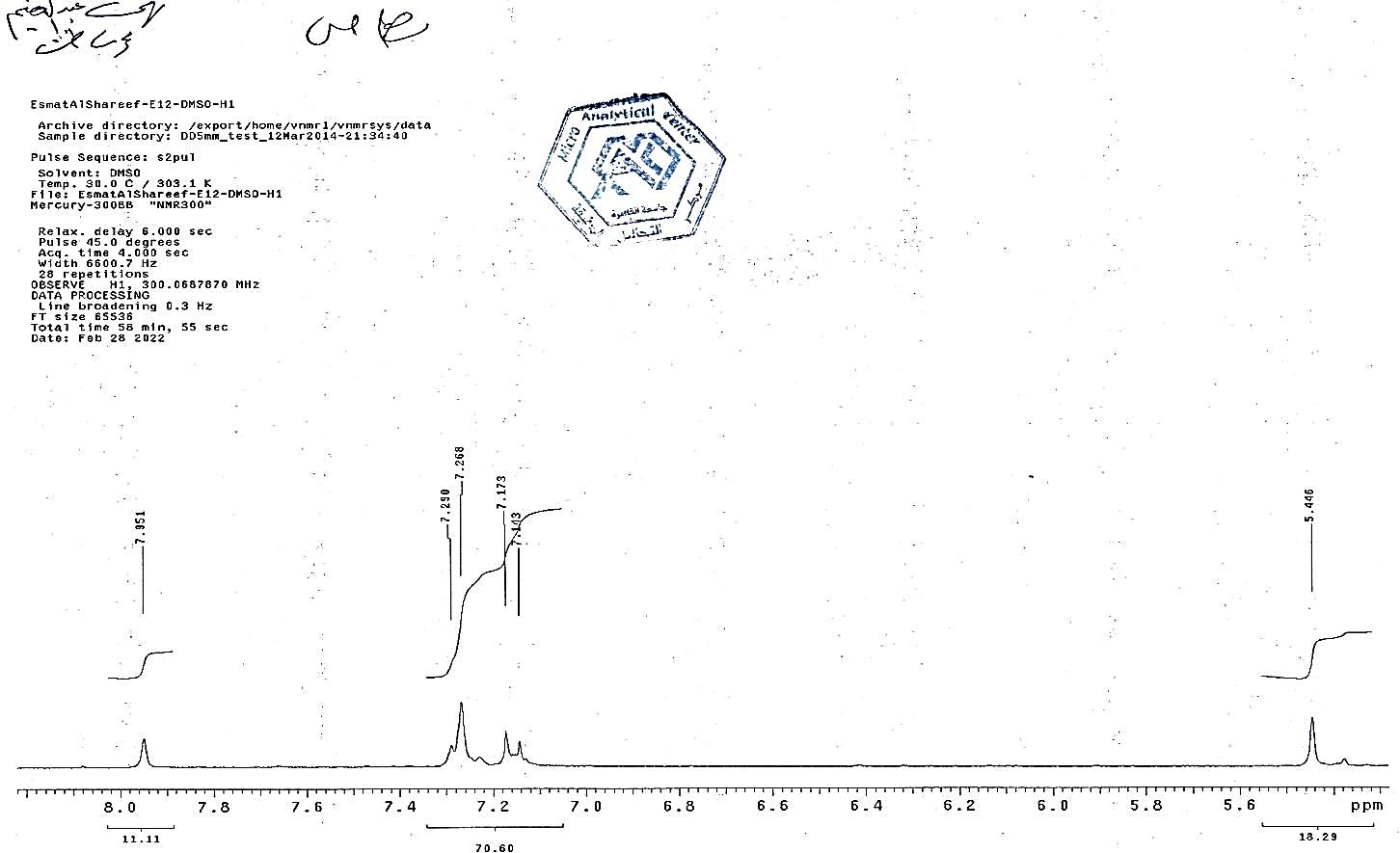
**

**Figure 56.** Part of the ^1^H-NMR spectrums for compound **5b.**

**
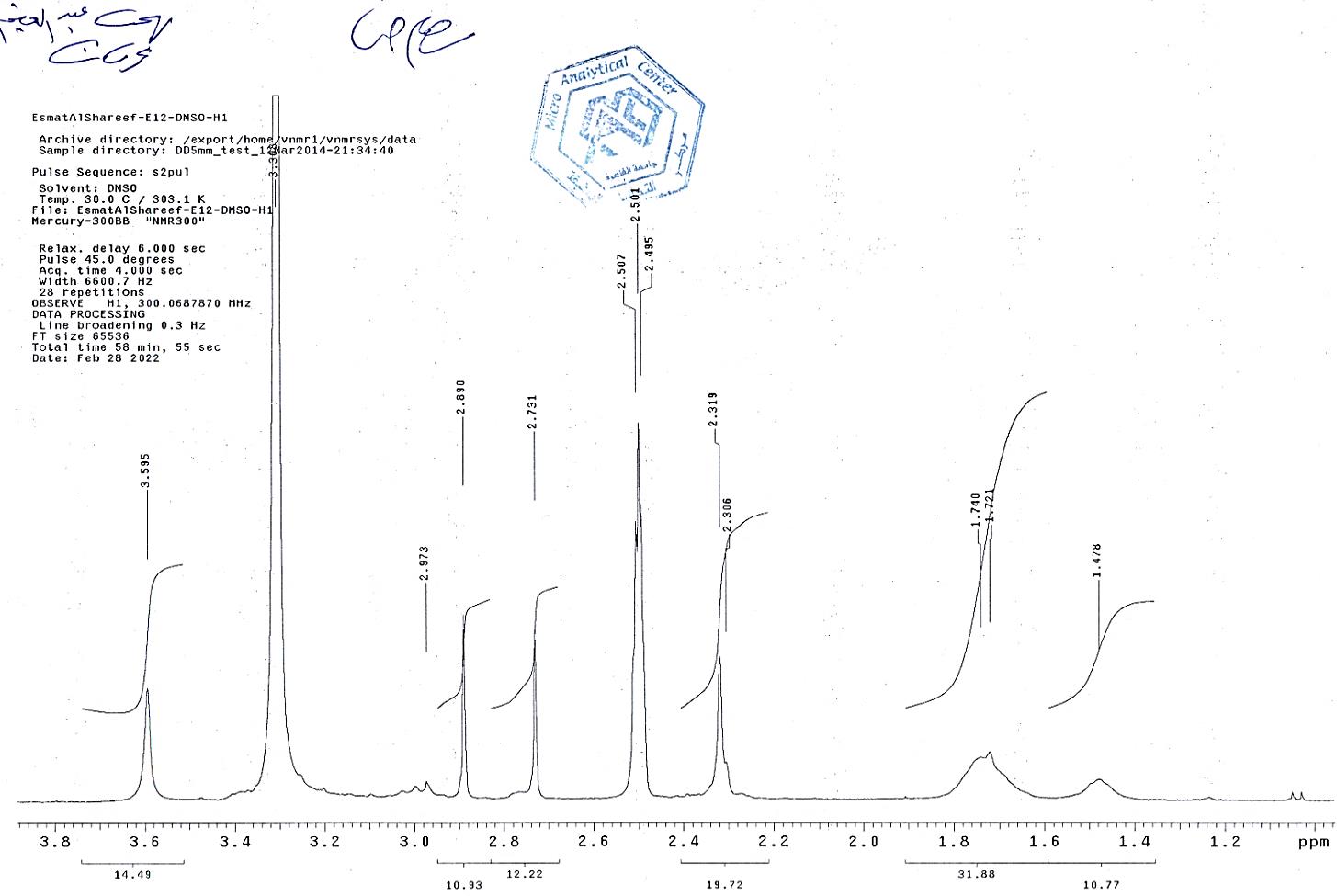
**

**Figure 57.** Part of the ^1^H-NMR spectrums for compound **5b.**

**
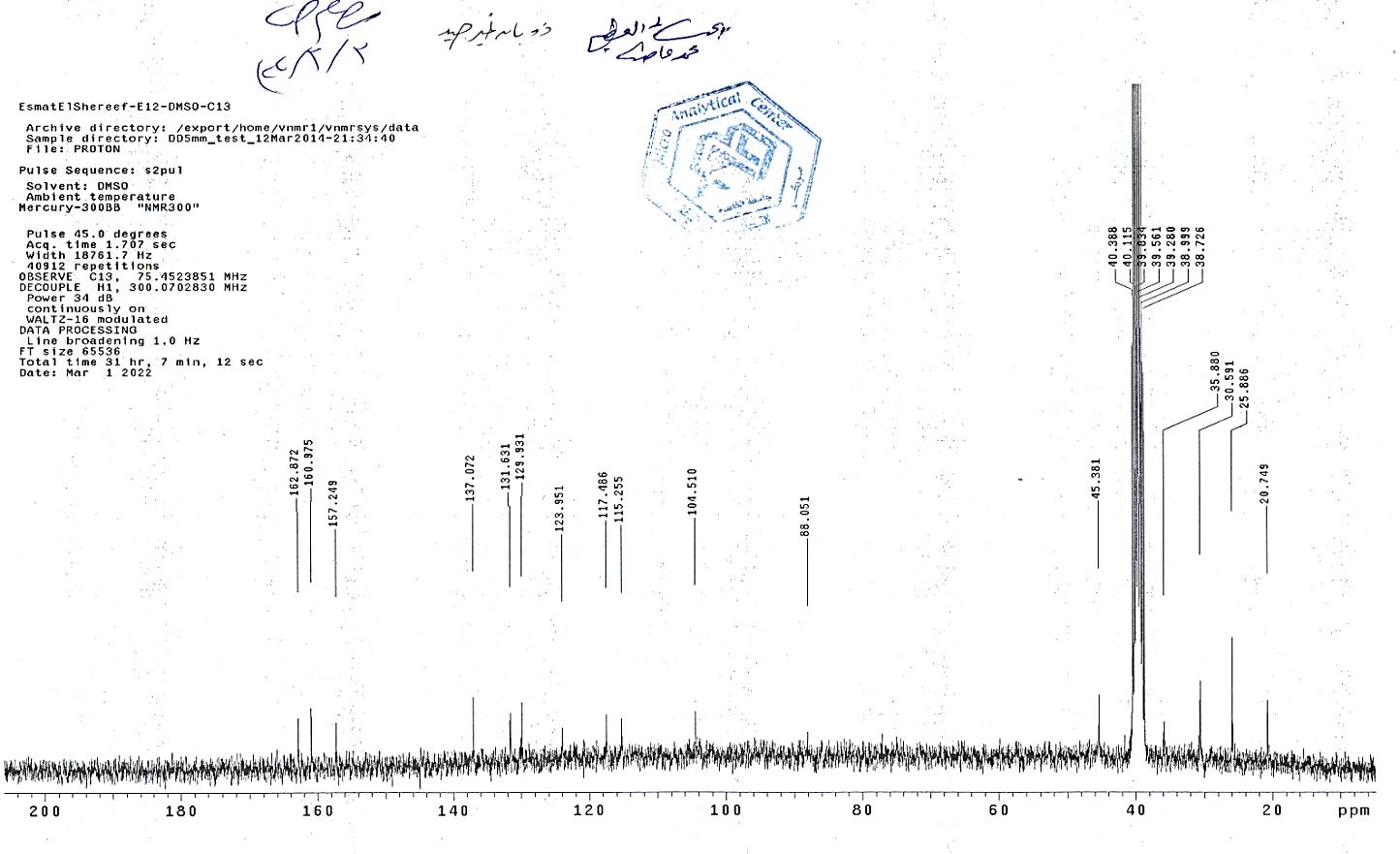
**

**Figure 58.** ^13^C-NMR spectrums for compound **5b.**

**Spectral data for compound 5c.**

**
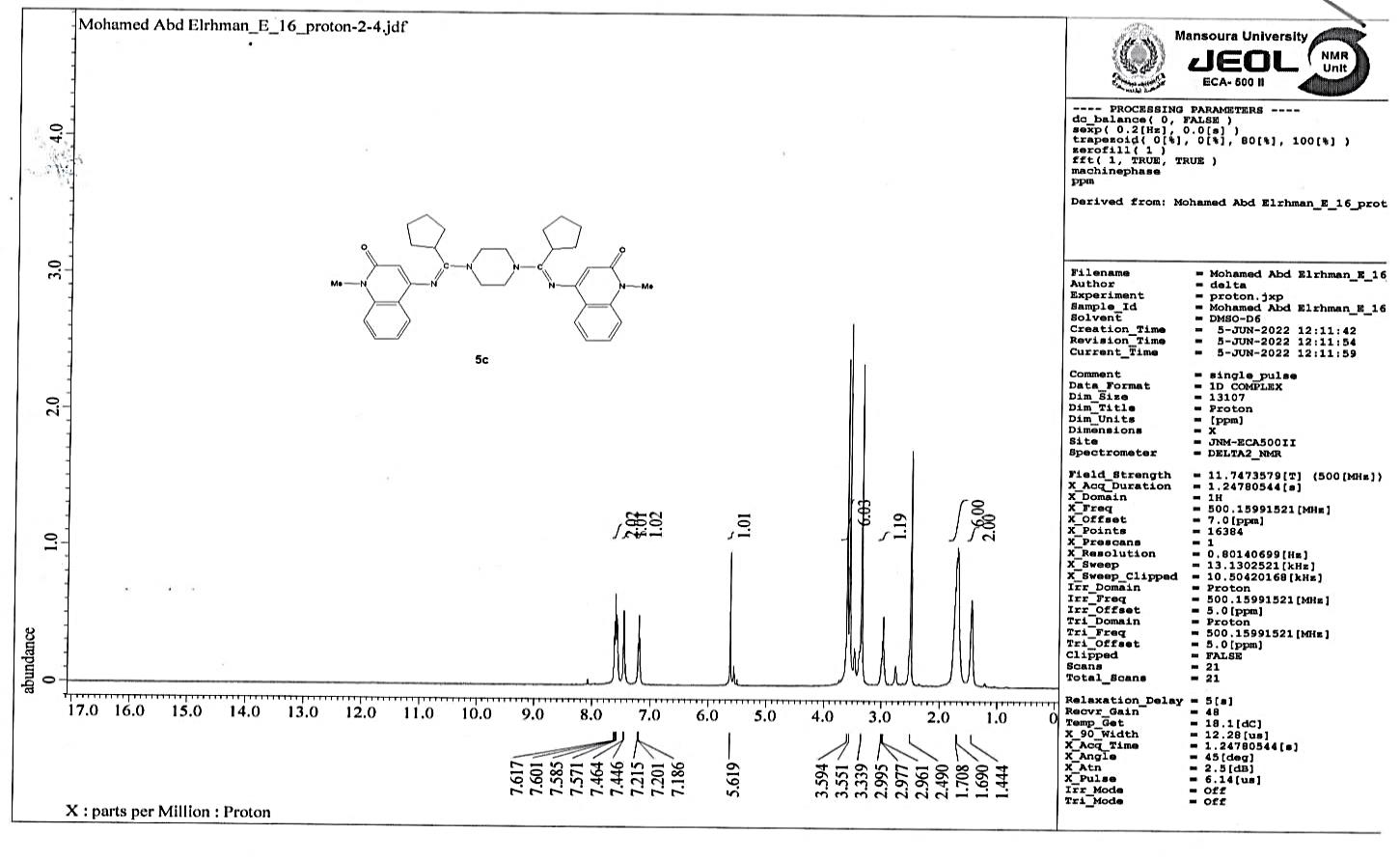
**

**Figure 59.** ^1^H-NMR spectrums for compound **5c.**

**
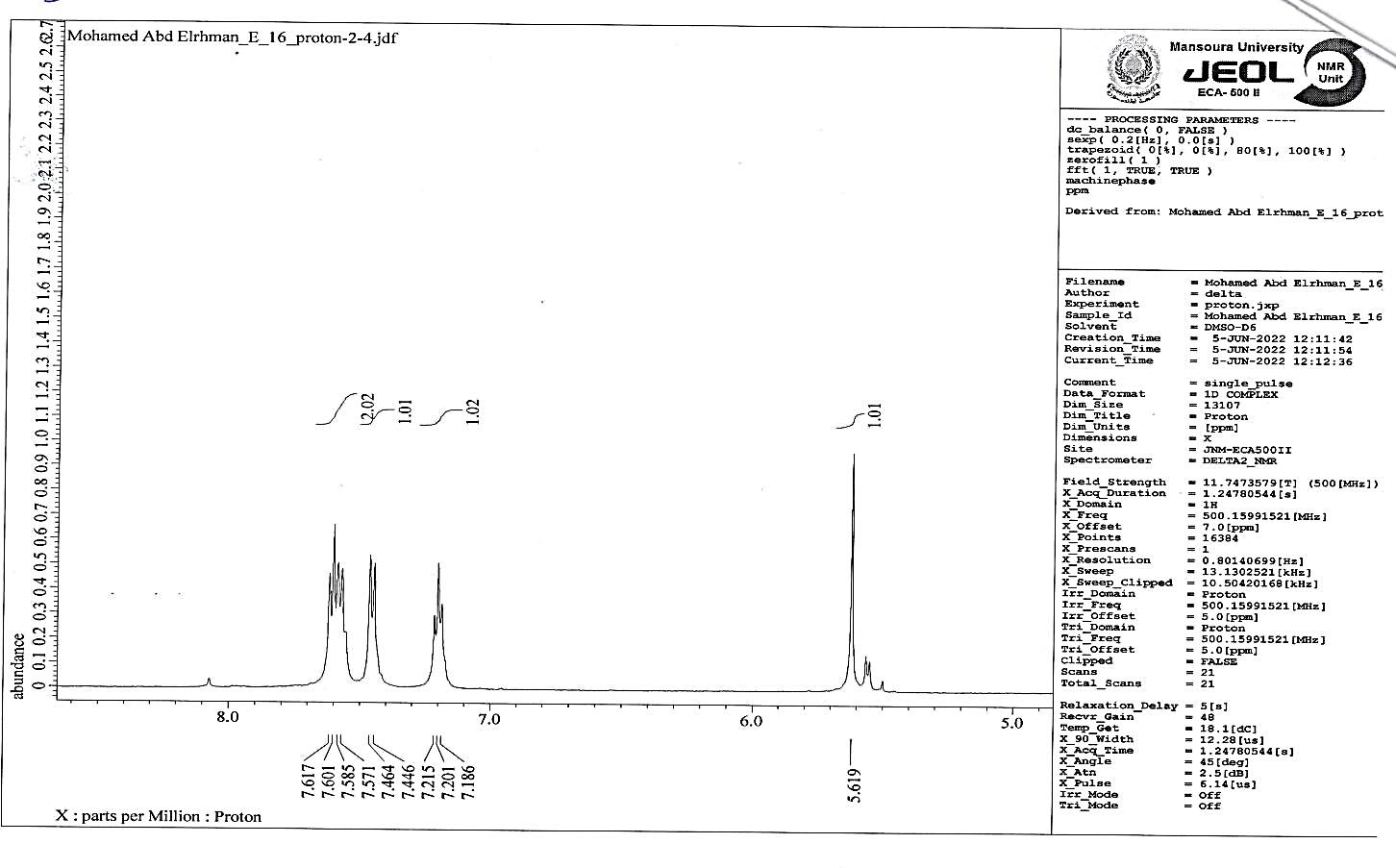
**

**Figure 60.** Part of the ^1^H-NMR spectrums for compound **5c.**

**
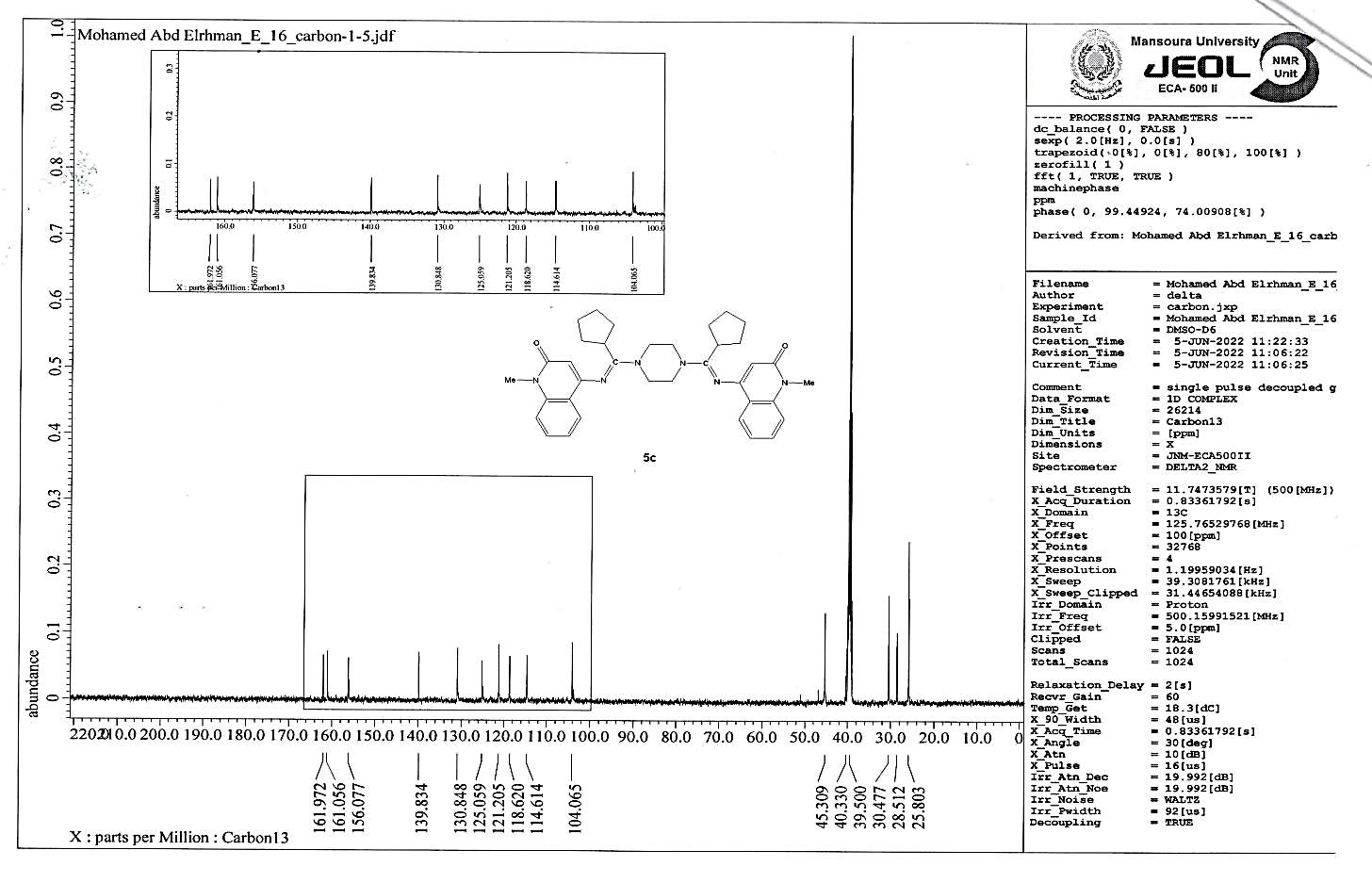
**

**Figure 61.** Part of the ^1^H-NMR spectrums for compound **5c.**

**
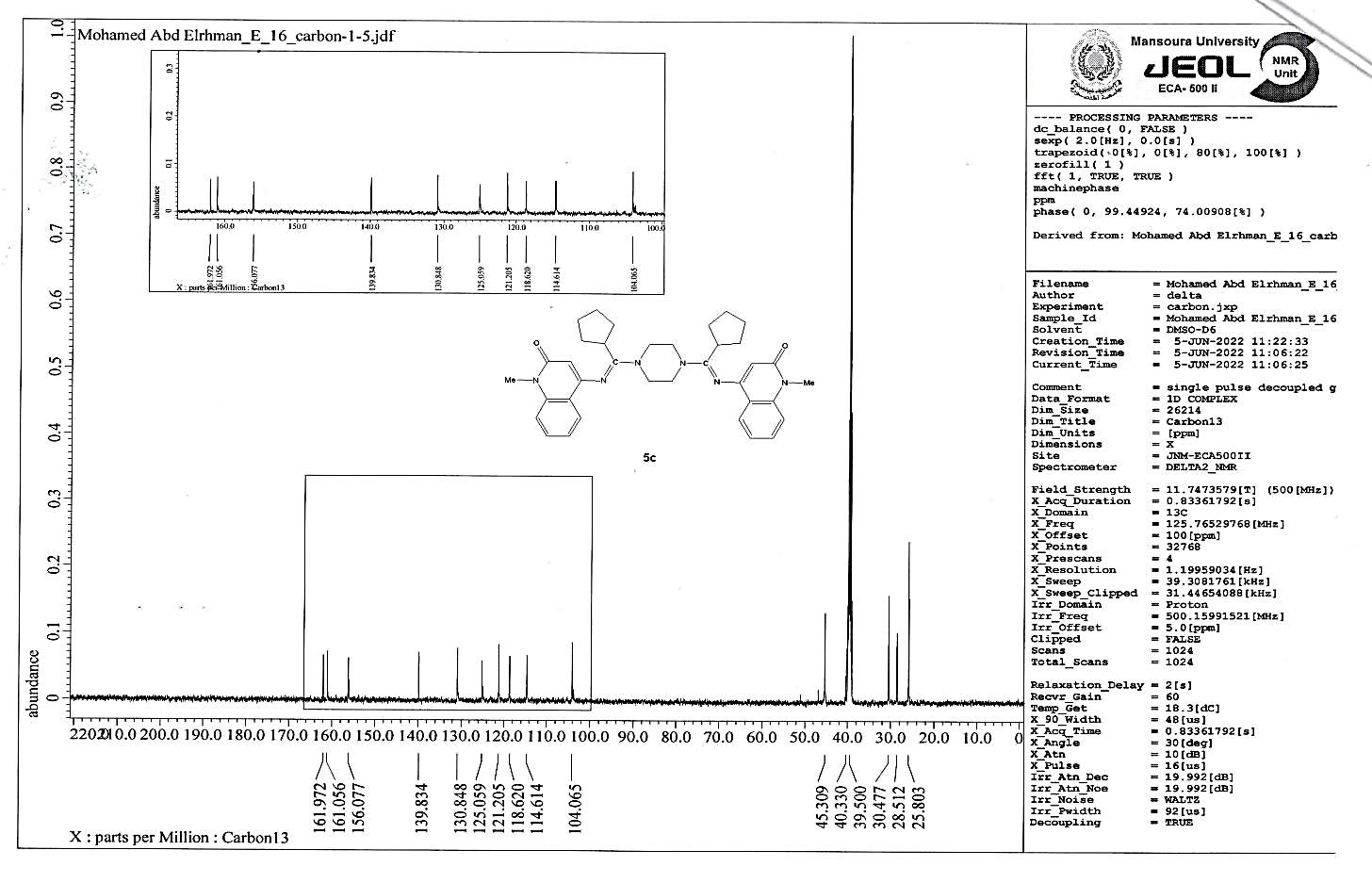
**

**Figure 62.** ^13^C-NMR spectrums for compound **5c.**

**Elemental analysis for new compounds 4a-l and 5a-c.**

**
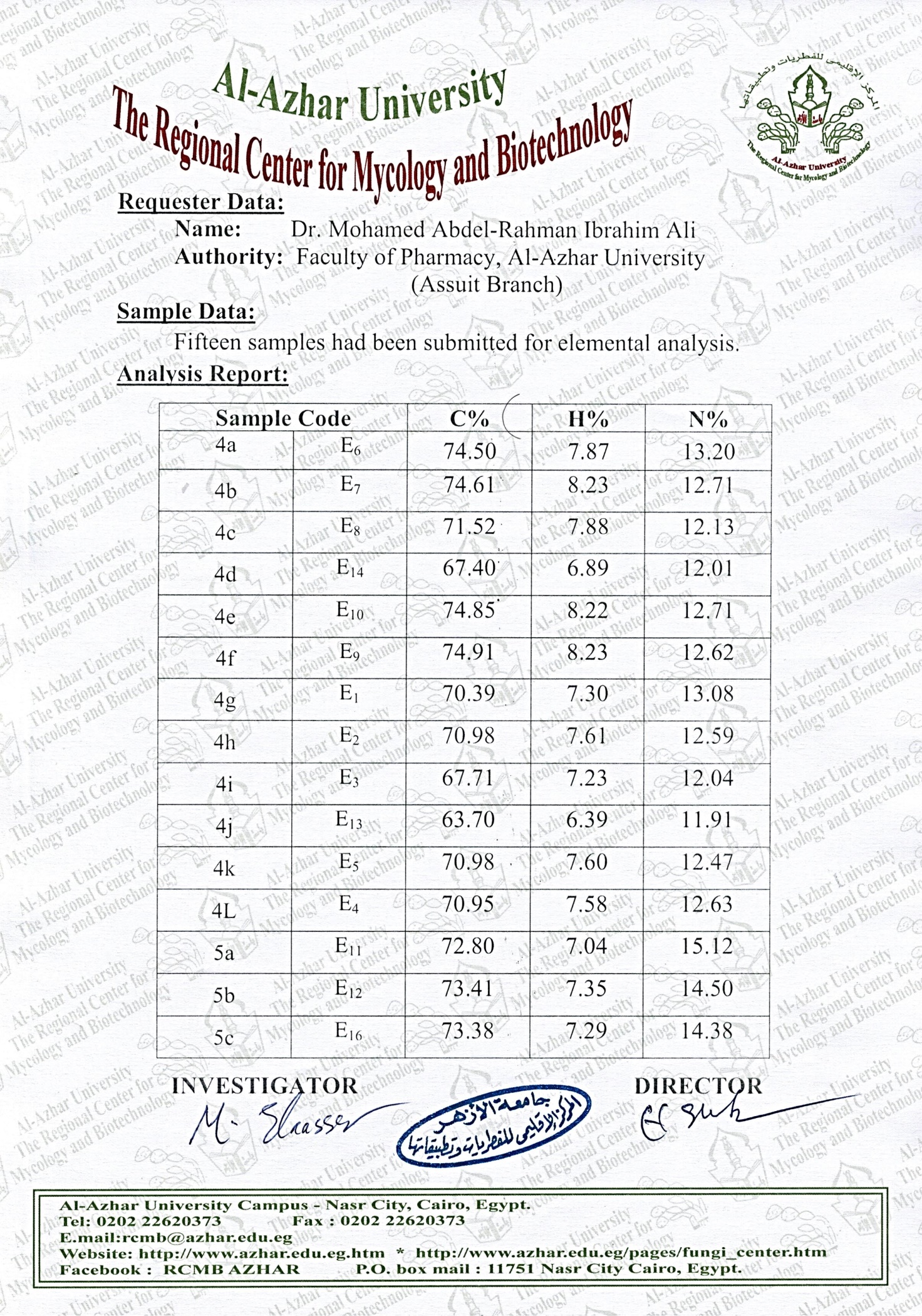
**

**Appendix A**

**4. Experimental**

**4.1.** Chemistry

The reactions were monitored with TLC (thin-layer chromatography) on Merck alumina-backed TLC plates Pf_254_ using UV light. NMR spectra were measured in DMSO-*d_6_* on a Bruker spectrometer (300 MHz for ^1^H and 75 MHz for ^13^C and 500 MHz) at MICROANALYICAL CENTER, Cairo University, and El Mansoura University, Egypt. Correlations were established using ^1^H-^1^H COSY, ^1^H-^13^C, and NOESY experiments, Faculty of Pharmacy, El Mansoura University, Egypt. Chemical shifts (δ) are reported in parts per million (ppm) relative to Tetramethylsilane (TMS) as an internal standard, Chemical shifts are expressed in δ (ppm) versus internal Tetramethylsilane (TMS) = 0 ppm for ^1^H and ^13^C. Coupling constants are stated in Hz. As an internal standard, chemical shifts (δ) are reported in parts per million (ppm) relative to TMS. Coupling constants (*J*) are reported in Hertz (Hz). Splitting patterns are denoted as follows, singlet (s), broad (b), doublet (d), multiplet (m), triplet (t), quartet (q), broad of singlet (bs), doublet of doublets (dd), doublet of triplets (dt), a triplet of doublets (td), and doublet of a quartet (dq). All melting points were determined on Stuart's electrothermal melting point apparatus and were uncorrected. Mass spectra were recorded on a Finnigan Fab 70 eV at Al-Azhar University, Egypt. Elemental analyses recorded were at Regional Center for Mycology and Biotechnology, Al-Azhar University, Egypt.

**4.2. Biological evaluation**

**4.2.1. Cytotoxic activity using MTT Assay and evaluation of IC_50_**

**4.2.1.1. MTT assay**

MTT assay was carried out to study the effect of compounds on mammary epithelial cells (MCF-10A). The medium in which cells were propagated contained Dulbecco's modified Eagle's medium (DMEM)/ Ham's F-12 medium (1:1) supplemented with epidermal growth factor (20 ng/mL), hydrocortisone (500 ng/mL), insulin (10 μg/mL), 2 mM glutamine and 10% fetal calf serum. After every 2-3 days, the cells were passaged using trypsin ethylenediamine tetra acetic acid (EDTA). The cells were seeded at a density of 10^4^ cells mL^-1^ in flat-bottomed culture plates containing 96 wells each. After 24 h, medium was removed from the plates and the compounds in (in 0.1% DMSO) were added (in 200 μL medium to yield a final concentration of 0.1% v/v) to the wells of plates. A single compound was designated with four wells followed by incubation of plates for 96h at 37°C. After incubation, medium was removed completely from the plates followed by addition of MTT (0.4 mg/mL in medium) to each well and subsequent incubation of plates for 3h. MTT (along with the medium) was removed and DMSO (150μL) was added to each well of the culture plates, followed by vortexing and subsequent measurement of absorbance (at 540 nm) using microplate reader. The data are shown as percentage inhibition of proliferation in comparison with controls containing 0.1% DMSO.

**4.2.1.2. Assay for antiproliferative effect**

To explore the antiproliferative potential of compounds MTT assay was performed according to previously reported procedure using different cell lines to explore the antiproliferative potential of compounds propidium iodide fluorescence assay was performed using different cell lines. To calculate the total nuclear DNA, a fluorescent dye (propidium iodide, PI) is used which can attach to the DNA, thus offering a quick and precise technique. PI cannot pass through the cell membrane and its signal intensity can be considered as directly proportional to quantity of cellular DNA. Cells whose cell membranes are damaged or have changed permeability are counted as dead ones. The assay was performed by seeding the cells of different cell lines at a density of 3000-7500 cells/well (in 200µl medium) in culture plates followed by incubation for 24h at 37 °C in humidified 5% CO_2_/95% air atmospheric conditions. The medium was removed; the compounds were added to the plates at 10 µM concentrations (in 0.1% DMSO) in triplicates, followed by incubation for 48 h. DMSO (0.1%) was used as control. After incubation, medium was removed followed by the addition of PI (25 µl, 50µg/mL in water/medium) to each well of the plates. At -80 °C, the plates were allowed to freeze for 24 h, followed by thawing at 25^o^C. A fluorometer (Polar-Star BMG Tech) was used to record the readings at excitation and emission wavelengths of 530 and 620 nm for each well. The percentage cytotoxicity of compounds was calculated using the following formula:


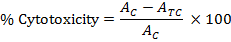


Where A*_TC_*= Absorbance of treated cells and A_C_= Absorbance of control. Erlotinib was used as positive control in the assay.

**4.2.2. Topo I and II inhibitory assay**

All the tested compounds were dissolved in DMSO and the DNA topoisomerase I and II inhibitory activity of each compound was measured as follows according to previously reported methods. Mixture comprising of 100 ng supercoiled pBR322 plasmid DNA (Thermo Scientific, USA) and 0.2 ~ 1 unit of recombinant human DNA topo I (Topo GEN INC., USA) or topo IIα (USB Corp., USA) was incubated with or without the prepared compounds in the assay buffer (For topo I, 10 mM Tris-HCl (pH 7.9), 150 mM NaCl and 0.1% BSA, 0.1 mM spermidine and 5% glycerol; for topo II, 10 mM Tris-HCl (pH 7.9), 50 mM NaCl, 50 mM KCl, 5 mM MgCl2, 1 mM EDTA, 1 mM ATP and 15 mg/mL BSA) for 30 min at 37 ◦C. The reaction with final volume of 10 μL was stopped by adding the topo stop buffer (For topo I, 10% SDS solution containing 0.2% bromophenol blue, 0.2% xylene cyanol and 30% glycerol; for topo II, 7 mM EDTA). The reaction products were electrophoresed on 0.8% agarose gel at 50 V for 1 h with TAE electrophoresis buffer. The gels were stained in an EtBr solution (0.5 mg/mL) and visualized by transillumination with UV light and were quantitated using Alpha Tech ImagerTM (Alpha Innotech Corp., USA).

**4.3. Statistical analysis**

Computerized Prism 5 program was used to statistically analyzed data using one-way ANOVA test followed by Tukey’s as post ANOVA for multiple comparison at P ≤.05. Data were presented as mean ± SEM.
